# Noncanonical Amino Acids: Bringing New-to-Nature Functionalities to Biocatalysis

**DOI:** 10.1021/acs.chemrev.4c00136

**Published:** 2024-09-27

**Authors:** Bart Brouwer, Franco Della-Felice, Jan Hendrik Illies, Emilia Iglesias-Moncayo, Gerard Roelfes, Ivana Drienovská

**Affiliations:** § Stratingh Institute for Chemistry, University of Groningen, Nijenborgh 4, 9747 AG, Groningen, The Netherlands; ‡ Department of Chemistry and Pharmaceutical Sciences, Vrije Universiteit Amsterdam, De Boelelaan 1105, 1081 HV, Amsterdam, The Netherlands

## Abstract

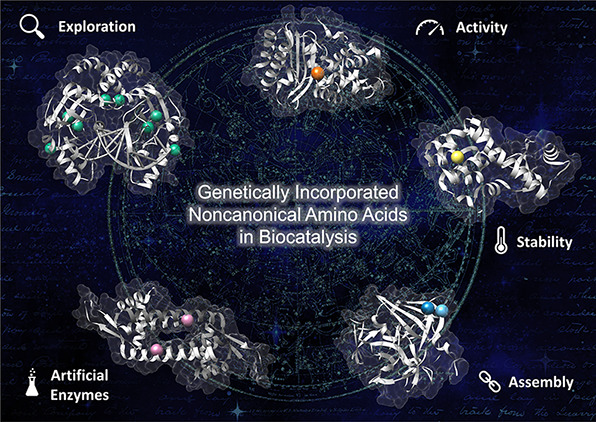

Biocatalysis has become an important component of modern organic chemistry, presenting an efficient and environmentally friendly approach to synthetic transformations. Advances in molecular biology, computational modeling, and protein engineering have unlocked the full potential of enzymes in various industrial applications. However, the inherent limitations of the natural building blocks have sparked a revolutionary shift. *In vivo* genetic incorporation of noncanonical amino acids exceeds the conventional 20 amino acids, opening new avenues for innovation. This review provides a comprehensive overview of applications of noncanonical amino acids in biocatalysis. We aim to examine the field from multiple perspectives, ranging from their impact on enzymatic reactions to the creation of novel active sites, and subsequent catalysis of new-to-nature reactions. Finally, we discuss the challenges, limitations, and promising opportunities within this dynamic research domain.

## 1 Introduction

Biocatalysis is becoming increasingly accepted within the organic chemistry community as an efficient and convenient method to achieve challenging transformations.^1−5^ Advances in molecular biology techniques, computational modeling, and protein engineering strategies have had a massive impact on the improvement of biocatalytic synthetic strategies under mild conditions and with novel modes of action.^6−8^ Of these, directed evolution has revolutionized the application of biocatalysis in a great number of areas,^9−11^ including the development of new-to-nature reactions when using protein hosts with or without promiscuous reactivity.^12−14^ This powerful feature to fit an enzymatic activity to our needs, combined with the “green credentials” that biocatalysis provides, is slowly transforming industrial process developments.^15−18^

However, enzymes are not miracle workers. Even though they are highly versatile and can achieve extremely complex transformations, they still fall behind compared to the chemical transformation repertoire that “conventional” organic chemistry has to offer. The use of both cofactors and post-translational modifications has been the way that nature filled some of this blank space of reactivity.^19^ In recent years there has been a lot of activity in combining bio- and chemocatalysis by the design of so-called artificial enzymes (ArEs), that is, biological hosts engineered to portrait an abiological substructure. A variety of approaches have been developed for the design of such ArEs, including covalent modification of proteins or DNA with synthetic scaffolds,^20,21^ protein ligation,^22^ cofactor replacement,^21,23^ supramolecular anchoring,^21,24^ and genetic incorporation of noncanonical amino acids (ncAAs),^25,26^ among others. Although all these strategies have brought great contributions to the field of biocatalysis, the latter stands out for its inherent mechanistic complexity and great diversity of functionalities that can be efficiently installed for a myriad of purposes.^27−29^

The genetic incorporation of ncAAs in proteins has been typically accomplished by following either an *in vitro* or *in vivo* approach. Both exploit the ribosome acyl-tRNA recognition and C-N bond formation versatility. The *in vitro* strategies concentrate on the synthesis of the desired acyl-tRNA, based on chemical-,^30−32^ aminoacyl-tRNA-synthetase- (aaRS-),^32,33^ or flexizyme-mediated^34,35^ acylation methodologies with the desired ncAA, to be used in a cell-free protein expression system. The *in vivo* methodologies, on the other hand, focus on the exploitation of the aaRS/tRNA pairs present in the biological host (usually *E. coli* or yeast), by following two strategies: selective pressure incorporation (SPI) and stop codon suppression (SCS).^36−42^ SPI relies on the promiscuity of the natural translation machinery to accept amino acids other than the canonical ones when these are absent from the growth media, or on the engineering of auxotrophic organisms. This methodology exploits the natural coding of one canonical amino acid (cAA) for the introduction of a structurally related ncAA throughout the entire sequence of the protein, hence achieving global substitution. The use of SPI is particularly advantageous when trying to study the global properties of certain proteins, such as conformational stability and folding properties.^43^ However, if the incorporation efficiency is not perfect, a statistical distribution of ArEs with varying degrees of ncAA incorporation will be obtained.

SCS complements this approach by using the natural coding for protein sequence conclusion, namely amber, opal and ochre nonsense codons, as the position for the ncAA introduction. To achieve this, a specific aaRS and tRNA system for the desired synthetic amino acid needs to be developed, requiring orthogonality with the other twenty cAAs and including the stop codon recognition. To date, more than ten types of aaRS/tRNA pairs (known as orthogonal translation systems, OTSs) have been developed, among which the tyrosyl-aaRS/^Tyr^tRNA_CUA_ pair from *Methanocaldococcus jannaschi* (*M*jTyr OTS) and the pyrrolysyl-RS/^Pyl^tRNA_CUA_ pairs from *Methanosarcina mazei* and *Methanosarcina barkeri* (*Mm*Pyl and *Mb*Pyl OTS, respectively) are the most popular.^27−29^ Within these systems, a plethora of ncAAs have been successfully incorporated into a vast array of proteins and enzymes. Excellent reviews have been published about the development of OTSs in the past few years, and the reader is referred to these for more detailed information.^25,26,44−53^ Next to that, an extensive repository was recently established by Icking et al., listing useful information on ncAAs and their method of incorporation into proteins.^54^ Compared to SPI, SCS allows for more precision, as it relies on a stop codon and, hence, is orthogonal to the sense codons used for cAAs. However, incorporation efficiency, in terms of both misincorporation of cAAs and premature termination, can sometimes be an issue. That means that the success of the method is both ncAA and protein dependent and that incorporation of more than one ncAA is not readily achieved. However, continuous improvements are reported, such as the development of improved OTSs, of special release factor 1 knockout bacterial strains that were developed to reduce the problem with termination processes,^55−57^ and of new strategies for the multiple incorporation of ncAAs.^37,58,59^ Notably, both strategies of ncAA incorporation are not exclusive to each other and can be combined, offering an attractive alternative for simultaneous heterogeneous substitutions with ncAAs.^55^

**Figure 1:**
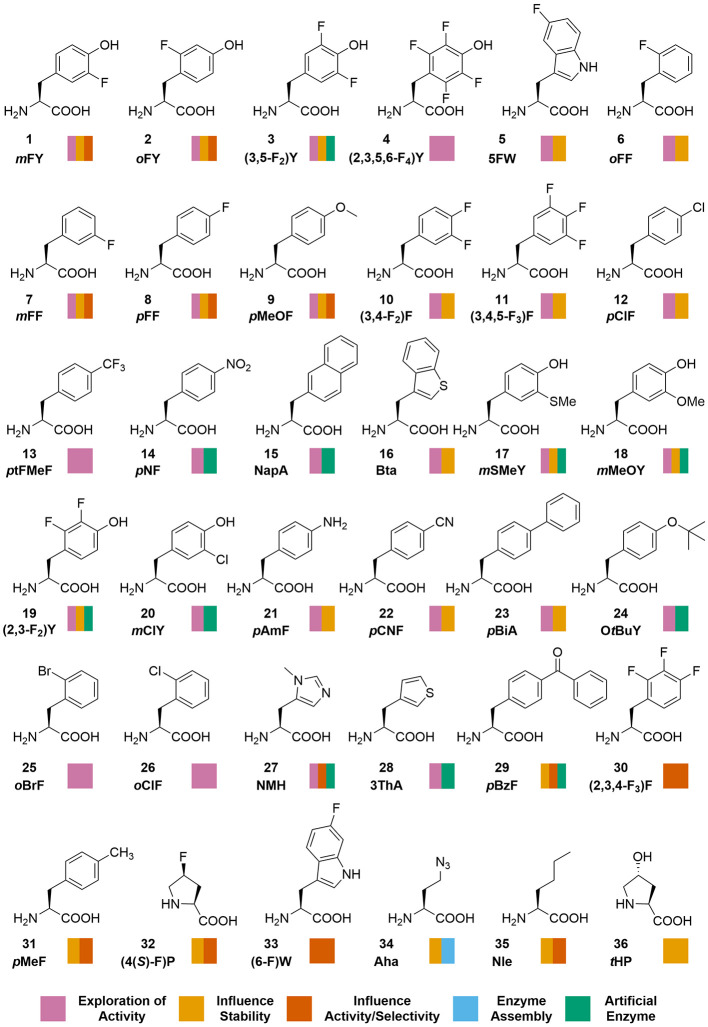

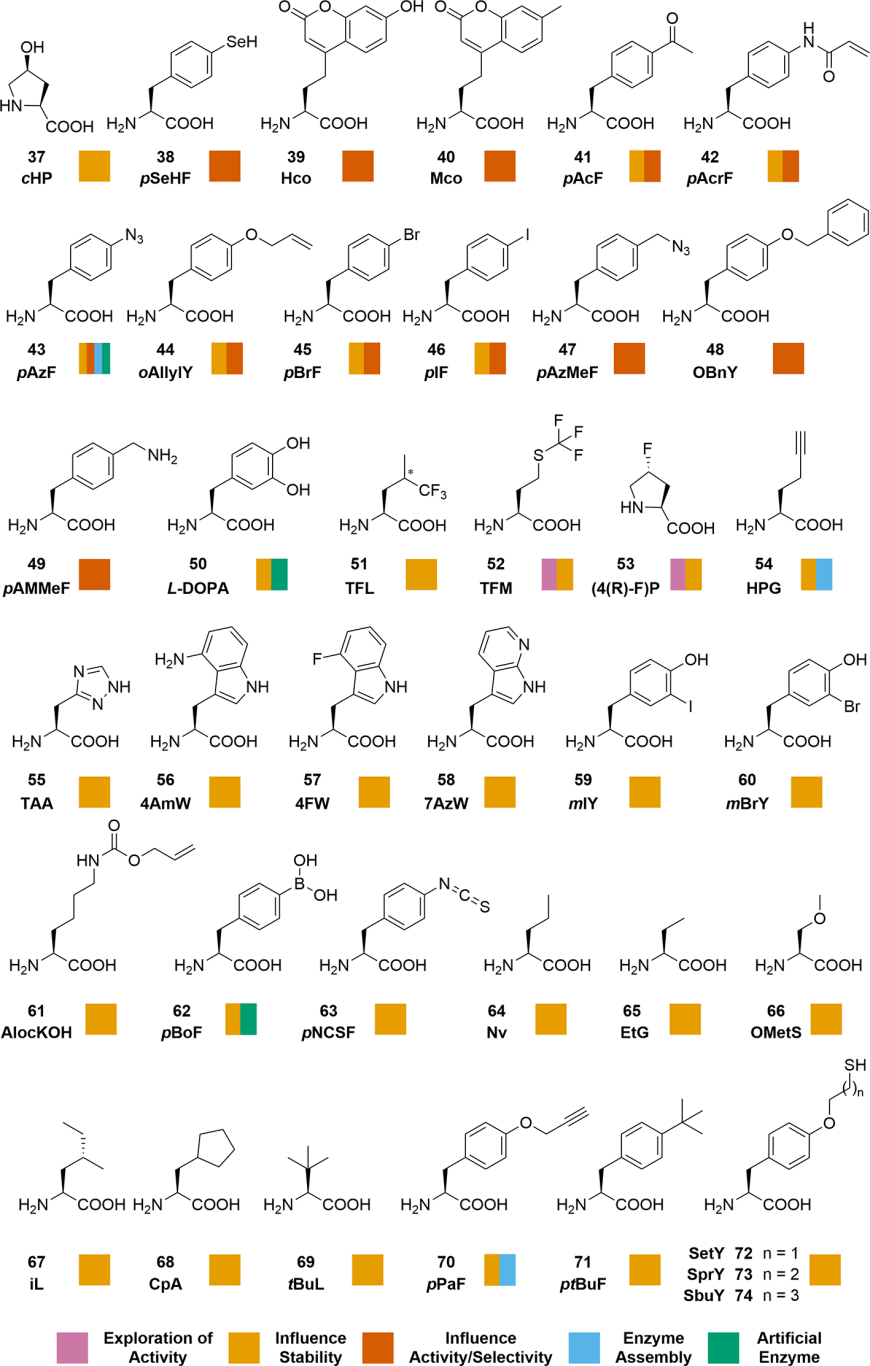

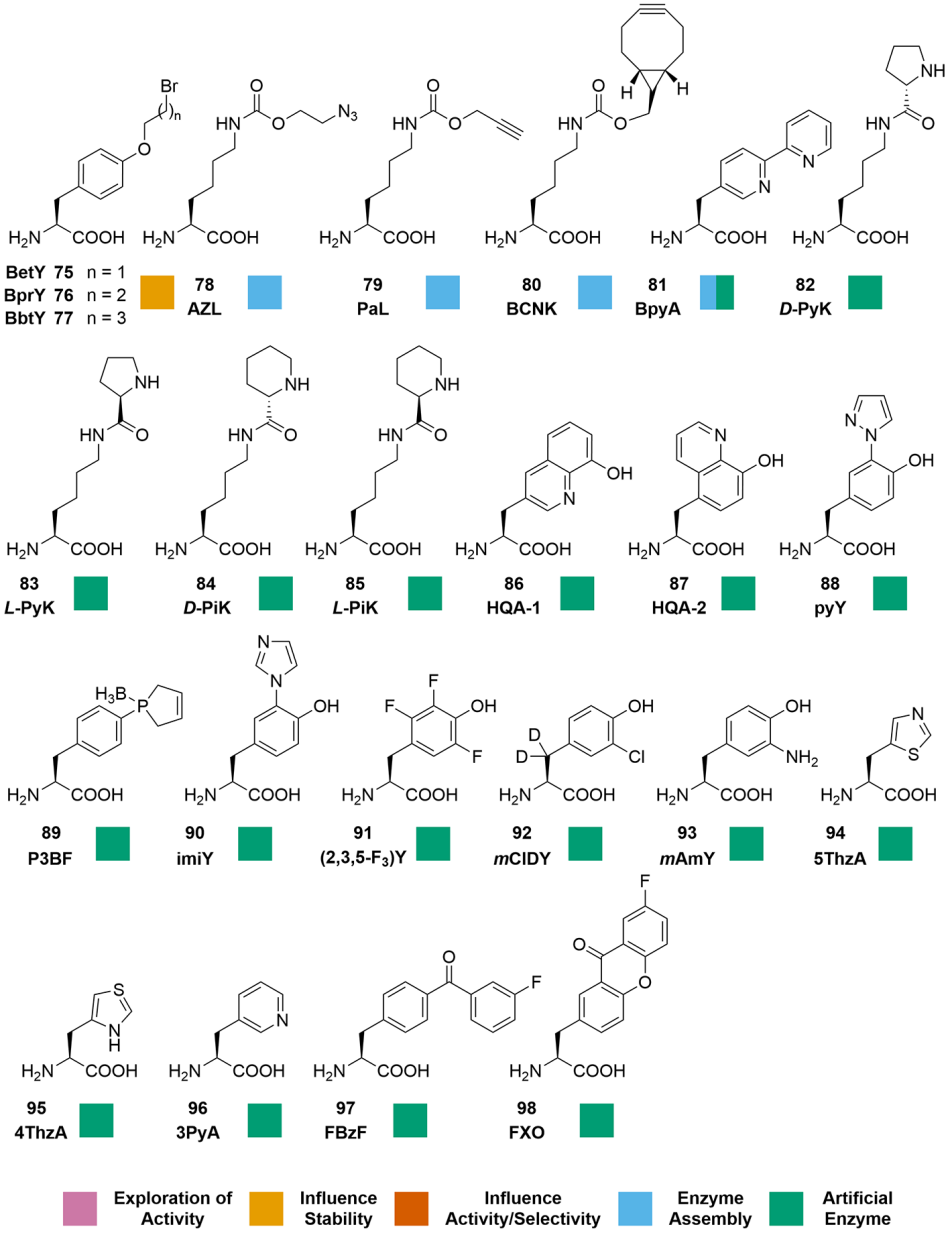
Structures of ncAAs incorporated in enzymes and their application in biocatalysis.

While there are many applications for ncAAs, including the investigation and mimicking of natural post-translational modifications^60,61^ or the thorough study of previously unknown mechanistic pathways in enzymes,^47,48,62^ we aim to provide a comprehensive account of the literature describing the use of *in vivo* genetically encoded ncAAs for advancing the field of biocatalysis. This review is organized in four sections: A) exploration of enzymatic activity; B) enhancement of enzymatic activity and stability; C) development of enzymatic assemblies; and D) design of artificial enzymes. Studies using pyrrolysine and selenocysteine have not been included, as these amino acids have naturally evolved OTSs.^63,64^Figure 1 gives an overview of the ncAAs’ structures incorporated in enzymes and employed in biocatalysis. In addition, a collection of tables summarizing the application and insights gained by the ncAAs into an enzyme or protein host can be found in the Supporting Information (Tables S3–S6).

## 2 Exploring Enzymatic Activity with Noncanonical Amino Acids

Site-directed mutagenesis has frequently been employed *en route* to unveil the role of key residues within the catalytic active site. Utilizing this technique in combination with ncAAs can lead to modifications in the reactivity of an enzyme by tuning the active site properties, such as p*K*_a_, redox potentials, and stabilization features. This section will describe those examples in which the activity of an enzyme was explored by the incorporation of a ncAA and discuss the insights gained by doing so, emphasizing their potential for biocatalysis. This section is organized based on enzyme classes.^47,48,62,65^

### 2.1 Transferases

Transferases are a class of enzymes that play an essential role in biocatalysis. They are known for transferring functional groups from one molecule to another. This ability makes them particularly useful for synthesizing complex molecules, as they can carry out reactions that would be difficult or impossible to achieve using conventional chemical methods.^66^

Glutathione S-transferases (GSTs) are a family of multigene isoenzymes involved in the addition of glutathione (GSH) to electrophilic substrates as a detoxification strategy,^67,68^ being particularly active in tumor processes.^69^ In vertebrate GSTs, the hydroxyl group of a conserved tyrosyl residue located near the N-terminus (domain I) is believed to stabilize the thiolate anion of GSH by H-bond interaction (TyrOH···SG^–^). Parsons and Armstrong verified this hypothesis by expressing tetradeca(*m*-fluorotyrosyl) GST, a variant with a ncAA showcasing a lower p*K*_a_ at the phenol group.^70^ The mutant was expressed in *E. coli* by SPI when growing in minimal media containing *m*-fluorotyrosine (**1**, ***m*FY**) instead of tyrosine. The expected increase in acidity, especially for the reactive residue 6, was evident from a 10-fold loss in activity compared to the native enzyme when using 1-chloro-2,4-dinitrobenzene (CDNB) as the substrate at pH > 8. The ***m*FY** residue would predominate as the thiolate species at high pH, hence losing its proton-shuttle ability that would allow a fine control for GSH stabilization. Observed was a moderate inverse kinetic solvent deuterium isotope effect for tetradeca(*m*-fluorotyrosyl) GST but no apparent effect for GST (0.5 ± 0.1 vs 0.9 ± 0.1, respectively), consistent with this hypothesis. Similar conclusions were obtained by Thorson et al. by selectively introducing *o*-fluorotyrosine (**2**, ***o*FY**), ***m*FY**, 3,5-difluorotyrosine (**3**, **(3,5-F**_**2**_**)Y**), and 2,3,5,6-tetrafluorotyrosine (**4**, **(2,3,5,6-F**_**4**_**)Y**) in human GST A1-1 *via in vitro* TAG codon suppression with a chemically aminoacylated suppressor tRNA_CUA_ strategy at the equivalent residue Y9.^71^

Parsons et al. focused as well on the influence of the H-bond formation with tryptophan residues located at domain I of the same enzyme and expressed tetra(5-fluorotryptophane) (**5**, **5FW**) GST by a similar SPI strategy.^72,73^ In this case, the enzyme showed unchanged turnover numbers for phenanthrene 9,10-oxide and 4-phenyl-3-buten-2-one, but a ∼4-fold increase for CDNB. This observation, together with the fact that the product release for the latter substrate is the rate-limiting step, indicated that the presence of fluorine in the mutant variant altered the kinetic properties of the enzyme primarily by enhancing the rate of product release. Further X-ray analysis^74,75^ showed that, in fact, whereas domains I (**5FW**)7 and (**5FW**)45 had little structural changes, domains II (**5FW**)146 and (**5FW**)214 seem to disrupt the H-bond between S209 and Y115, an interaction suggested to limit product release. These works demonstrate how the “hydrogen-to-fluorine” substitution can greatly alter the reactivity of an enzyme in terms of modification of electrostatics and steric properties.

Histone acetyltransferases (HATs) are a group of enzymes which acetylate conserved lysine amino acids in the *N*-terminal tails of nucleosomal histones as a regulation strategy of gene expression.^76^ The p300/CBP associated factor (PCAF) is a promiscuous HAT capable of acetylating both target histone H3 and the nonhistone p53 proteins, with a specific ratio (*k*_rel_) of 270 in favor of the former substrate. Montclare and collaborators were able to modulate the selectivity of this enzyme by using artificial variants containing *o-*, *m*- or *p-*-fluorophenylalanine (**6**, ***o*FF**; **7**, ***m*FF**; **8**, *****p***FF**) instead of the respective cAA.^77^ A global substitution of the 10 phenylalanine residues present in PCAF was achieved through SPI by using an *E. coli* auxotrophic strain, reporting incorporation levels of 73–88%. While variant deca(*****p***FF**) PCAF presented a ∼14-fold loss in *k*_rel_, variant deca(***m*FF**) PCAF showcased complete selectivity toward histone H3 with a comparable activity as the native enzyme. The third variant deca(***o*FF**) PCAF, on the other hand, had a complete loss of activity toward either substrate. Although the mutated residues do not directly interact with the substrate, the authors suggested that the interactions with neighboring residues are probably influencing the overall packing and stability of the protein. In a related work, the same research group reported a general loss of overall structure when a similar global substitution strategy was performed on the HAT *Tetrahymena* general control nonderepressor 5 with its native substrate histone H3.^78^

### 2.2 Lyases

Lyases facilitate the cleavage of various chemical bonds. They are central to many biological activities and are found in a wide range of organisms.^79,80^ In biotechnology, lyases have shown considerable potential. Practical applications have been found in industries such as textiles, paper production, juice purification, and oil extraction.^81^

Terpenoid cyclases are known to catalyze polycyclization reactions of linear polyenes *via* carbocation formation. The success of cyclases relies on their ability to preorganize their substrates in their binding pocket to achieve effective stereoelectronic interactions and transient carbocation stabilization.^82^ X-ray crystal structures of several terpene cyclases show aromatic residues lined up through the active pocket, thus providing a perfect electron density-rich environment for carbocation stabilization.^83^

Morikubo et al. studied the contribution of the cation−π interaction on the stabilization of cationic squalene intermediates by mutating the active site residues F365 and F605 in *Alicyclobacillus acidocaldarius* squalene-hopene cyclase (AaSHC; technically classified as an isomerase) with cAAs and ncAAs.^84^ Replacing F605 with ***p***-methoxyphenylalanine (**9**, *****p***MeOF**) (*via* SCS), tyrosine and tryptophan, presenting comparable or higher cation-π binding energies than phenylalanine (tyrosine: 26.9, tryptophan: 32.6, phenylalanine: 27.1 kcal/mol),^85^ enhanced the reaction rates below 40 °C compared to the wild type (WT) AaSHC but decreased the activity at higher temperatures. Position F365, on the other hand, showed a similar trend only with the tyrosine mutation, where F365*****p***MeOF** and F365W variants proved detrimental to activity. These unexpected results were proposed to be correlated to structural changes within the mutants. The active-site region near position 365 would be more compact than the region surrounding position 605; hence, it is expected to be more susceptible to steric changes. In effect, F365*****p***MeOF** and F365W variants presented the highest *K*_M_ values among the tested mutants. Furthermore, these two variants exhibited a decreased Cotton effect, suggesting disorganization within the protein architecture. Switching the residues with electrodeficient *****p***FF**, 3,4-difluorophenylalanine (**10**, **(3,4-F**_**2**_**)F**), and 3,4,5-trifluorophenylalanine (**11**, **(3,4,5-F**_**3**_**)F**), all three incorporated in a cell-free translation system at both F605 and F365 positions, not only decreased the specific activity but promoted the formation of prematurely cyclized products.

**Scheme 1:**
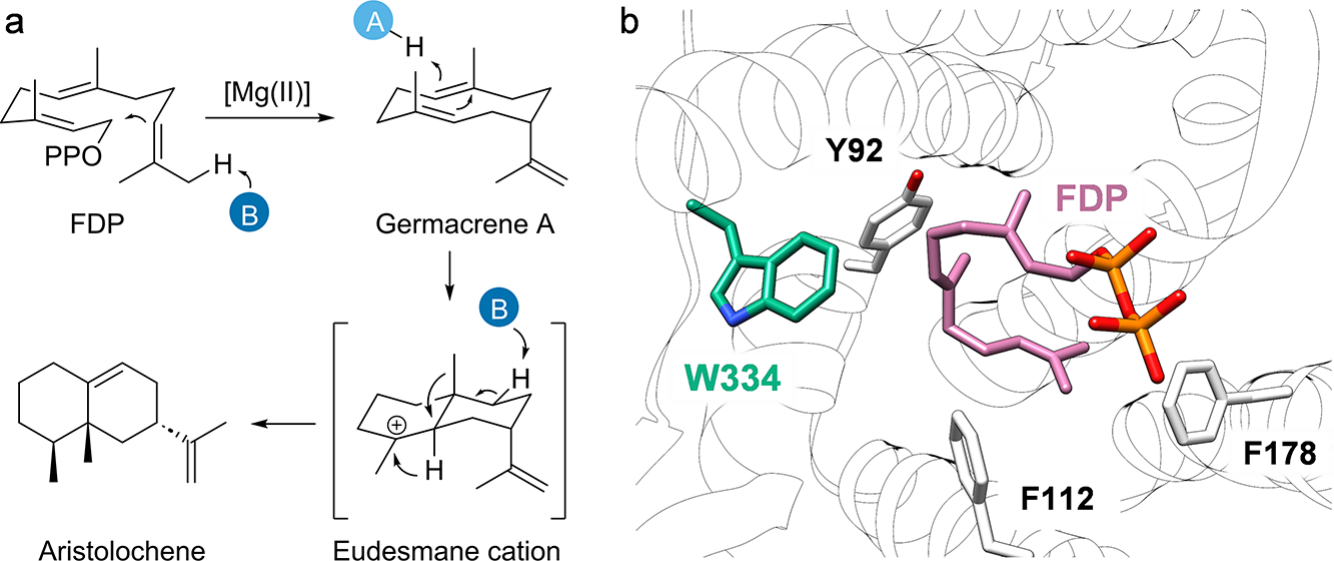
(a) Biosynthesis of Aristolochene: A, acid; B, base. (b) Position 334 in the active site of PrAS with FDP bound (derived from PDB: 1F1P).

**Figure 2:**
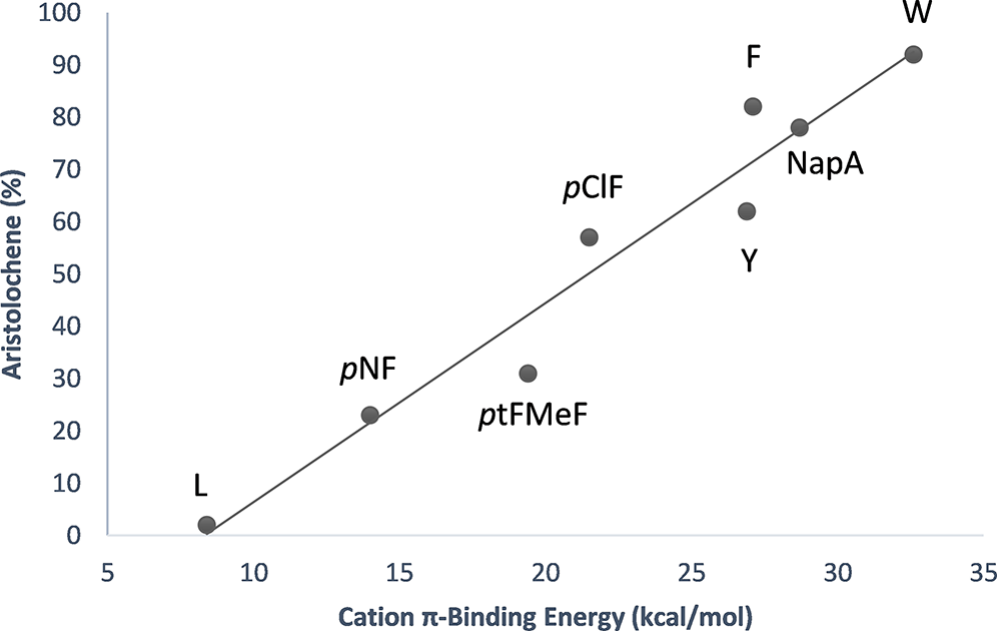
Relationship between tabulated cation−π binding energies and aristolochene produced (R^2^ = 0.95) by the different variants of PrAs_W334**ncAA** conducted by Faraldos et al.^86^

A similar study was conducted by Faraldos et al. with aristolochene synthase from *Penicillium roqueforti* (PrAS).^86^ The enzyme catalyzes the cyclization of farnesyl diphosphate (FDP) to macrolide germacrene A, followed by the formation of transient cationic intermediates, as eudesmane cation, to produce aristolochene (Scheme 1). The replacement of W334 with leucine and tyrosine, and with ncAAs *p*-chlorophenylalanine (**12**, *****p***ClF**), *p*-trifluoromethylphenylalanine (**13**, *****p***tFMeF**), *p*-nitrophenylalanine (**14**, *****p***NF**), and 2-napthylalanine (**15**, **NapA**) *via* SCS led to the accumulation of either aristolochene or germacrene depending on the electron-withdrawing properties of each amino acid. Additionally, the experimental product distribution was in good accordance with tabulated cation−π binding energies (Figure 2). All together, these examples strongly demonstrate that cation−π interaction occupies a key position in the catalytic mechanism by terpene cyclases.

### 2.3 Oxidoreductases

Oxidoreductases are a diverse category of enzymes that enable electron exchange, hydrogen extraction, hydride transfer, and oxygen integration within living organisms. Typically, redox reactions involve at least two substrates - one oxidative and one reductive. Most oxidoreductases are nicotinamide cofactor-dependent enzymes with a strong affinity for NAD or NADP.^87^

Thioredoxin (Trx) is a dimeric flavoprotein ubiquitous to all organisms and is engaged in antioxidant processes. It is involved in the reduction of disulfide bonds on target proteins by using highly conserved thiols groups, C29 and C32, in a thiol-disulfide exchange mechanism.^88^ Englert, Nakamura, Wang, et al. developed a *Mm*Pyl OTS for the incorporation of (*S*)-2-amino-3-(benzothiophen-3-yl)propanoic acid (**16**, **Bta**) at position 28 of Trx from *Staphylococcus aureus*, a residue that was found to shield the active site of the enzyme from solvent as well as to interact *via* hydrogen bonds with D58 in the active pocket.^89^**Bta** can participate in hydrogen bond formation processes only as an acceptor, which would result in a higher electron density at position 28, while maintaining a similar bulky aromatic site as tryptophan. When embedding both Trx_WT and Trx_W28**Bta** in the reduction cascade of oxidized ArsC, Trx, TrxR and NADPH/H^+^, the latter variant exhibited a 1.3-fold increase in activity. In contrast, mutant Trx_W28A exhibited a 5-fold decrease of catalytic efficiency. These results suggest that position 28 is important for both the steric and the electrostatic properties that can contribute to the active pocket of Trx. A higher π aromatic electron density offered by Bta28 seems to promote better C29 thiolate formation, resulting in an increased redox efficiency.

**Scheme 2:**
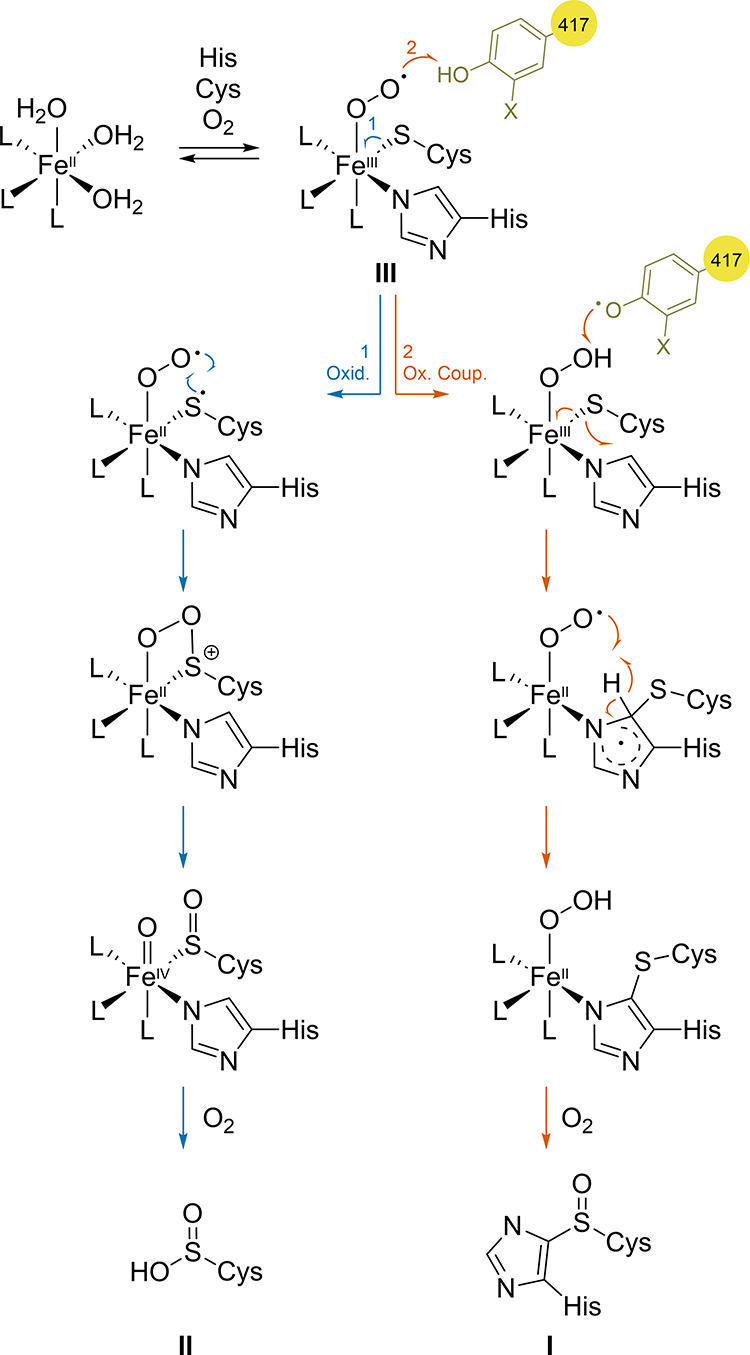
Proposed OvoA Mechanism for the Formation of **I** and **II** by Zhao, Liu and Coworkers^90^ Oxid.: oxidation; Ox. Coup.: oxidative coupling. X = H (Y), SMe (**17**), OMe (**18**).

OvoA from *Erwinia tasmaniensis* is a non-heme iron enzyme responsible for the first step in ovothiol biosynthesis. OvoA naturally presents two types of reactivities: oxidative C-S coupling between cysteine and histidine, which leads to **I**, and cysteine dioxygenase activity, producing cysteine sulfinic acid (**II**) (Scheme 2). Zhao, Liu, and co-workers were able to identify Y417 as an active site residue of OvoA_WT and introduced ncAAs (*S*)-2-amino-3-(4-hydroxy-3-(methylthio)phenyl)propanoic acid (**17**, ***m*SMeY**)^90^ and (*S*)-2-amino-3-(4-hydroxy-3-methoxyphenyl)propanoic acid (**18**, ***m*MeOY**)^91^ at this position *via* amber-codon suppression.^92^ Both ncAAs present relatively similar p*K*_a_ values compared to that of tyrosine but lower reduction potentials. The new variants, OvoA_Y417[***m*SMeY/*m*MeOY**] were able to fine-tune this dual activity, in both cases changing the parent product ratio from 9:1 in favor of **I** to ∼7:3. The solvent kinetic isotope effect (KIE) with the OvoA_Y417***m*SMeY** variant on *k*_*ox-coup*_ was found to be 2.09 ± 0.02, providing evidence that the cysteine sulfinic acid and oxidative coupling products are produced from a common intermediate. In addition, OvoA_Y417***m*MeOY** displayed an inverse deuterium KIE when deuterium-labeled histidine was used as a substrate, suggesting that the C-S bond formation present in **I** precedes the sulfoxidation reaction. These findings provided support for a mechanism where products **I** and **II** would come from the same superoxo species **III**, with Y417 acting as a crucial redox modulator that controls the enzyme activity (Scheme 2).

**Scheme 3:**
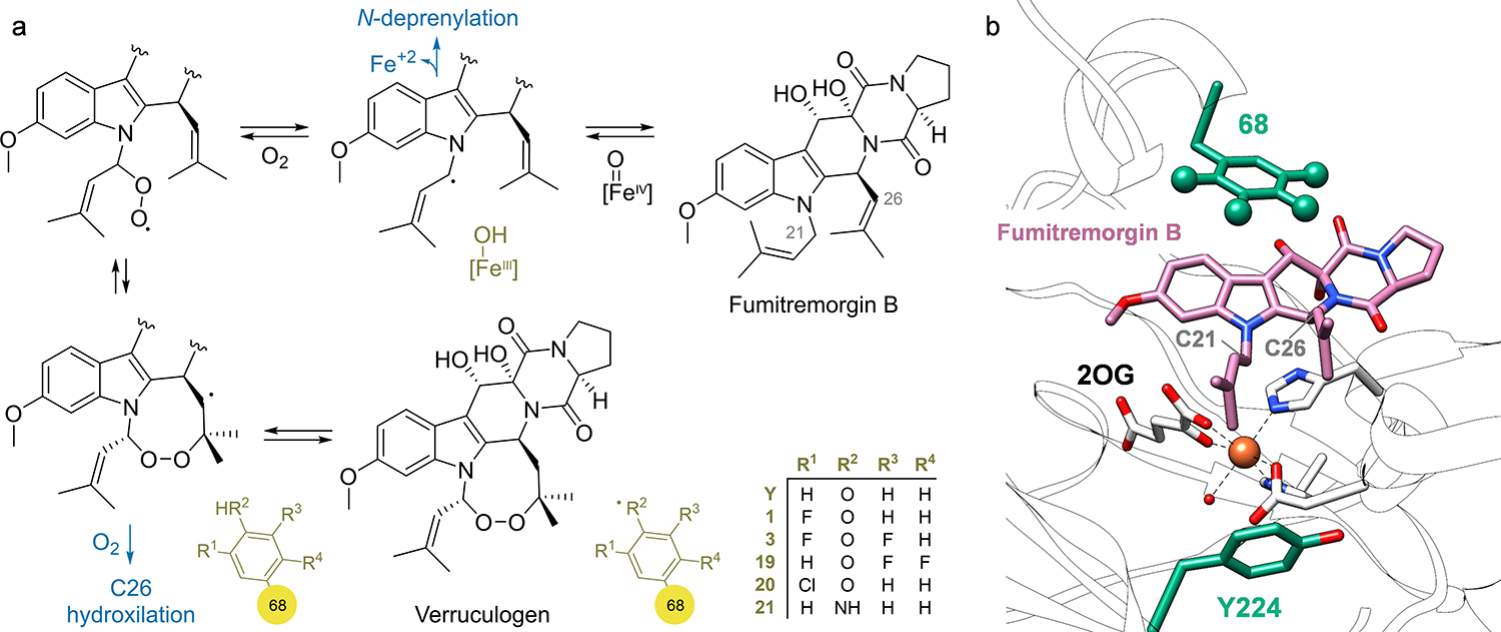
(a) Proposed Role of Residue 68 in the Biosynthesis of Verruculogen by Lin, Silakov, Krebs, Boals, Bollinger, and coworkers. (b) Position 68 in the Active Site of FtmOx1 Bound with Fumitremorgin B (from PDB: 7ETK)^93^

Verruculogen synthase, also known as fumitremorgin B endoperoxidase, FtmOx1, from *Aspergillus fumigatus* is a non-heme enzyme capable of capturing O_2_ and installing a cyclic endoperoxide between carbons 21 and 27 of fumitremorgin B and produce verruculogen. Lin, Silakov, Krebs, Boals, Bollinger, and co-workers employed a variety of genetically incorporated cAAs and ncAAs, including ***m*FY**, 2,3-fluorotyrosine (**19, (2,3-F**_**2**_**)Y**), **(3,5-F**_**2**_**)Y**, *m*-chlorotyrosine (**20**, ***m*ClY**) and *p*-aminophenylalanine (**21**, *****p***AmF**), through SCS, to investigate the biocatalytic pathway of verruculogen.^93^ Analysis of the oxidized product distribution of fumitremorgin B with the different variants highlighted that Y68 participated directly in the mechanism of the reaction as an electron shuttle, but Y224 had no essential role. The fine-tuning of the redox potential of Y68 with (n)cAAs as well as performing experiments in deuterated buffer revealed a delicate single electron mechanism that FtmOx1 must follow for the effective production of verruculogen (Scheme 3).

**Scheme 4:**
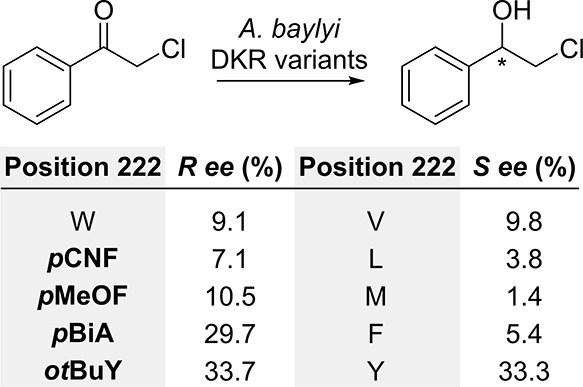
Enantioselective Reduction of 2-Chloro-1-phenylethanone by *A. baylyi* DKR Variants

Diketoreductase (DKR) from *Acinetobacter baylyi* ATCC 33305 is a homodimeric enzyme capable of enantioselective reduction of mono- and diketones to chiral alcohols.^94^ Crystal structure analysis^95^ showed tryptophan residues at positions 149 and 222 to be important for binding, with the latter positioned at the hydrophobic dimeric interface and proposed to influence the entrance direction and binding orientation of substrates to the pocket. Ma, Yang, et al. studied the steric influence of the amino acid at position 222 on the enantiomeric selectivity when 2-chloro-1-phenylethanone was used as substrate.^96^ For this, a set of variants was produced by using cAAs and ncAAs (*via* SCS) with different side chain sizes. Those mutants portraying residues with a smaller molecular volume than tryptophan (valine, leucine, methionine, phenylalanine, tyrosine and *p*-cyanophenylalanine (**22**, *****p***CNF**) showed a change of enantioselectivity favoring the isomer *S*, while those variants with a larger steric volume (*****p***MeOF**, *p*-biphenylalanine (**23*p*BiA**), *o*-*tert*-butyltyrosine (**24**, ***ot*BuY**)) retained preference for the *R* product (Scheme 4). This inversion in enantiomeric preference could be caused by a binding pocket shape change. Docking studies suggested that mutants containing smaller residues at position 222 would create a large entrance into the hydrophobic pocket. This wider form would allow substrates to enter the active center with a flexible orientation without steric hindrance, where hydrophobic interactions would be favored. In contrast, substrate orientation would fall into a more steric-guided binding pattern for the four mutants having bulky amino acid substitutions. In this scenario, the authors argued that when the *S*-forming mode predominates, the substrate would preferentially bind with its phenyl group pointing toward the active pocket. Interestingly, the *S*-favoring mutants showed between 1.3- to 5.9-fold reduced activity, whereas the *R*-favoring enzymes presented a 64.6- to 70.2-fold increase, except for DKR_F222***p*OMeF** (∼5-fold decrease). The observed differences in catalytic efficiency were mainly related to changes in *K*_M_ rather than *k*_cat_, highlighting the importance of position 222 in substrate binding.

### 2.4 Hydrolases

Hydrolases are a diverse group of enzymes that use water to catalyze chemical bond cleavage efficiently. They play an essential role in the metabolism of many natural and synthetic compounds.^97^ Lipases are a subclass of serine hydrolases that catalyze the hydrolysis of triglycerides to fatty acids and glycerol. In industrial biocatalysis, lipases are considered one of the most important enzymes.^98,99^

**Scheme 5:**
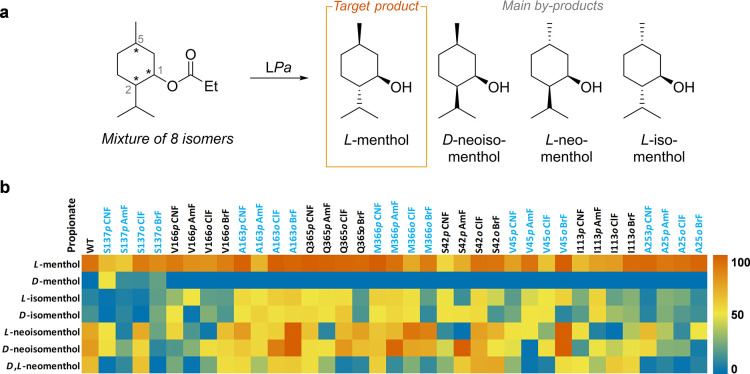
(a) Hydrolysis of a Mixture of Menthol Propionate by L*Pa* and the Main Products Obtained. (b) Yield Heatmap of the Eight Isomers Hydrolyzed for Each L*Pa*_ncAA Variant^101^

A lipase produced by *Pseudomonas alcaligenes* (L*Pa*) has been reported to selectively hydrolyze racemic diastereomeric mixtures of menthyl propionate (8 isomers) to obtain *L*-menthol in moderate diastereoisomeric excess (*de* = 50%) at high conversions (87%) (Scheme 5).^100^ Yu et al. aimed to increase the enzyme selectivity by systematically incorporating sterically hindered *o*-bromophenylalanine (**25**, ***o*BrF**) and *o*-chlorophenylalanine **(26**, ***o*ClF)** and polar ***p*AmF** and ***p*CNF** by stop codon suppression in 9 different positions at the binding pocket: S137 (near substrate’s C-1), A163, V166, G365, M366 (near substrate’s C-2), S42, V45, I113 and A253 (near substrate’s C-5).^101^ From this library, more than half of the variants increased the *de* of the starting material by >30% (Scheme 5b). Molecular dynamics (MD) simulations showed a linear correlation between the *de*, the pocket solvent excluded volume, and the average B-factor (an indicator of backbone flexibility/rigidity)^102^ of region 250–260. Variant L*Pa*_A253***o*BrF** was the variant that experimentally gave higher *de* (95%) at high conversions (95%), in line with the MD predictions, albeit with only 7.4% of enzymatic activity when compared to L*Pa*_WT. Considering that residue A253 is positioned next to catalytic triad member H252, responsible for the proton transfer during hydrolysis, further calculations on dihedral angles and hydrogen bond formation indicated that an increased bulkiness at position 253 would induce a ‘locked’ proton-transmitting H252. Thus, a stronger recognition of *L*-menthol propionate would develop, clarifying the observed high *de* and reduced activity from the mixture of isomers.

Endonucleases are enzymes that catalyze the cleavage of phosphodiester bonds within a polynucleotide chain, such as DNA or RNA. The *Pvu*II restriction endonuclease from *Proteus vulgaris* is a type II restriction endonuclease that cleaves DNA between the central GC base pair of its recognition sequence (5′-CAGCTG-3′) in a Mg^II^-dependent reaction, resulting in blunt-ended products.^103^ The catalytic activity of restriction enzymes is related to their conformation, and subtle side-chain substitution may lead to substantial changes. Using *Pvu*II endonuclease as a model system, Dominguez et al. investigated the effects of globally incorporating ncAAs ***o*FF**, ***m*FF**, and ***p*FF** through SPI in the enzyme.^104^ Each *Pvu*II endonuclease subunit features four phenylalanine residues located far from the active site. Expression in *E. coli* by using minimal media loaded with the respective ncAA resulted in incorporation efficiencies in the range of 7–17%. Judged by the formation of λ-DNA cleavage patterns, the artificial variants showed no change in specificity compared to the native *Pvu*II, but an ∼2-fold increase and ∼0.5-fold decrease in specific activity was observed for the ***m*FF** and ***p*FF** mutants, respectively. Additionally, an ∼0.8-fold loss in conformational stability was noted for the latter variant. Thus, it was highlighted how subtle changes in side-chain structures at locations far from the active site can affect activity and stability.

### 2.5 Isomerases

Isomerases are a distinct group of enzymes that carry out a variety of chemical transformations within the molecule itself.^105^ Glucose isomerase, for example, is one of the most important industrially produced enzymes. This particular enzyme is used on a large scale in the production of high fructose corn syrup.^106^

**Scheme 6:**
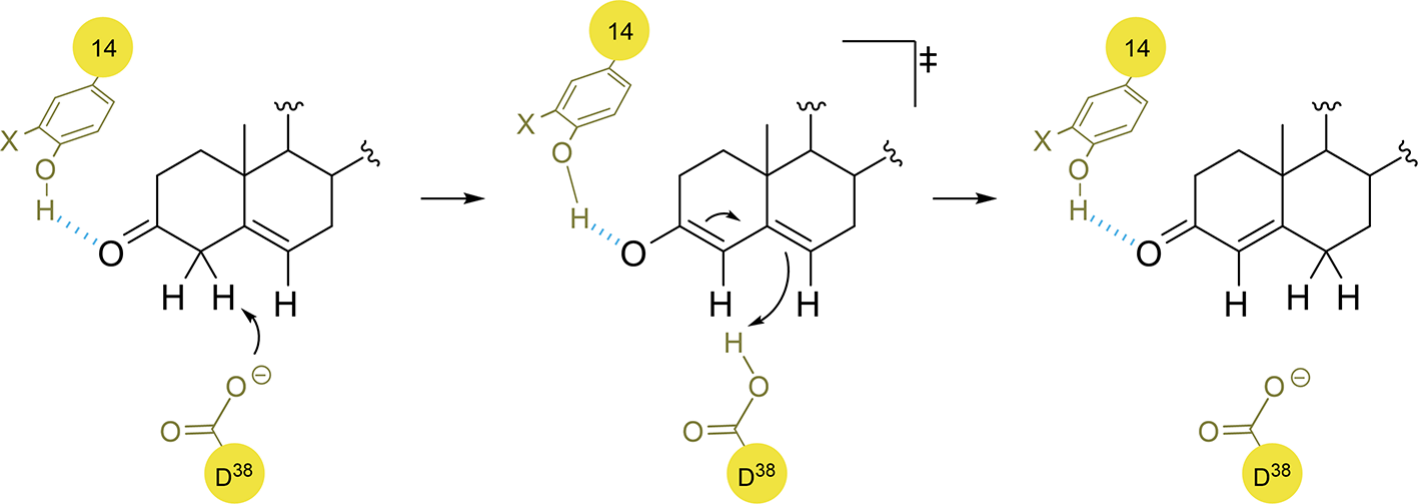
Reaction Mechanism for KSI-Catalyzed Isomerization from *C. testosteroni* The strength of the H-bond between residue 14 and the C-3 carbonyl group, modulated if X = H (Y) or F (**7**), influences the dienolate- or dienol-like transition state character.

Ketosteroid isomerase (KSI) can catalyze a proton transfer from C4β to the C6β position of a variety of Δ^5^-3-ketosteroids (Scheme 6).^107,108^ Brook and Benisek studied the role of Y14 of KSI from *Comamonas testosteroni* (CotKSI) as a Brønsted acid activator of the carbonyl group of 5-androstene-3,17-dione (5-AND) by replacing this residue with the more acidic ncAA ***m*FY** (p*K*_a_ difference of 1.5 log units) *via* SPI.^109^ For that, a variant where the other two tyrosine residues in the enzyme at positions 55 and 88 were mutated to phenylalanine was produced (CotKSI*) and used to express the mutant enzyme. Variant CotKSI*_Y14***m*FY** showed a 4-fold decrease in *k*_cat_ while maintaining the *K*_M_ when compared to the parent enzyme CotKSI*, implying that the acidity of the phenolic moiety at position 14 is highly important for the enzyme activation mode. This residue actively establishes a H-bond with the C3-carbonyl group of the substrate and would promote a dienol-like transition state when the acidity of the phenol group is dropped.

In the reaction catalyzed by KSI from *Pseudomonas putida* (PpKSI), the keto-enolization by the heterolytic C-H bond cleavage of the C4 proton is promoted by D40, followed by the γ-attack. In the intermediate state, the negative charge of the dienolate is stabilized by an oxyanion hole between a tyrosine triad and D103. Electrostatic interactions are often suggested to play an important role in stabilization of reactive intermediates and have been appointed as a great contributor for KSIs’ mode of action.^110^ To explore the electric field exerted in the pocket of PpKSI by the vibrational Stark effect (VSE), Boxer and collaborators genetically incorporated ***m*ClY***via* SCS at different positions of the active site. As a result, a systematic decrease in *k*_cat_/*K*_M_ for substrates 5(10)-estrene-3,17-dione^111^ and 5-AND was observed,^112^ providing ideal models for electric field/catalytic proficiency correlations. A direct relationship between activity and the electric field/H-bond network was established, suggesting that the stabilizing effect of KSIs’ extended H-bond network is of electrostatic origin.

Völler, Budisa, et al. have also reported the study of the electrostatic component in the pocket of cytochrome c through the genetic incorporation of ***p*CNF** and VSE analysis of the -CN reporter motif, revealing a redox-linked long-range modulation of local electric fields.^113^

**Scheme 7:**
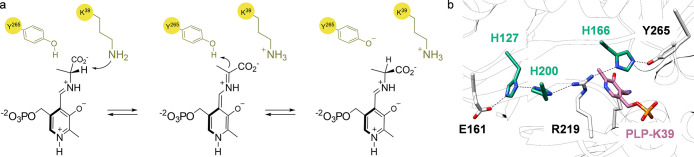
(a) Equilibrium between the Aldimides and Quinonoid Intermediates in the *B. stearothermophilus* Alanine Racemase Proposed Mechanism. (b) Proposed Extended H-Bond System for the Activation of Y265 (PDB: 1SFT)

Alanine racemases are pyridoxal 5′-phosphate-dependent enzymes that can interconvert *L*- to *D*-alanine, and vice versa, at high speed. They have attracted increasing attention, as they are involved in the formation of peptidoglycans in cell bacterial walls, making them promising drug targets.^114^ The alanine racemase from *Bacillus stearothermophilus* employs a two-base mechanism in which K39 and Y265 residues act as catalytic residues during the equilibrium between the aldimides and quinonoid intermediates (Scheme 7a).^115^ Sharma and collaborators identified an extended charge-relay and hydrogen bonding network involving residues E161, H127, H200, R219 and H166 that could potentially alter the p*K*_a_ of Y265, hence affecting the catalytic activity of the enzyme (Scheme 7b).^115^ To confirm this structural activation, the authors employed the SCS technique to change the histidine residues located at the proximal 166 and at the distal 127 and 200 positions with *N*-δ-methylhistidine (**27**, **NMH)** and (*S*)-2-amino-3-(thiophen-3-yl)propanoic acid (**28**, **3ThA)**, two amino acids presenting a similar shape as histidine but whose side chains cannot act as H-bond donor. Circular dichroism analysis indicated that the six mutant enzymes produced, three for each ncAA, folded similarly to the wild-type enzyme. Their performance, however, was greatly affected. Between 150- and 600-fold decrease in *k*_*cat*_ was observed for all the mutants, hence confirming the importance of the H-bond network for catalytic activity. This study represents a convincing example of how the fine-tuning of the hydrogen bonding properties of a residue can lead to the understanding of the structural and mechanistic activation present in an enzyme beyond their active pocket.

Throughout the analyzed examples, a frequent approach stands out: the replacement of a particular residue (or set of residues) important for catalysis with ncAAs with specific properties that could affect the mechanism or outcome of the reaction carried out by the enzyme, yielding significant insights into enzymatic functions. There is still potential for further utilization of ncAAs in this direction by developing and introducing new ncAAs that more closely mimic natural interactions or introduce new ones, thereby expanding our understanding of enzyme reactivity even further.

## 3 Improving Enzymes with Noncanonical Amino Acids

In line with the extensive and ever-increasing applications of enzymes in the biotechnological industry, the need to enhance enzymatic properties has remained a constant in meeting industrial demands.^116^ The most popular approach for enhancing the properties of biocatalysts is enzyme engineering, e.g. directed evolution, which involves iterative rounds of mutation with cAAs and selection to evolve enzymes with desired properties.^117^ Upon the initial exploration of ncAAs in biocatalysis, it became evident that these molecules can unlock changes to enzymatic properties beyond the reach of the cAAs alone. This section focuses on applications of ncAAs that have led to improved activity, selectivity, or stability of natural enzymes.

### 3.1 Influencing Activity and Selectivity

#### 3.1.1 Transferases

Transketolases (TKs) are thiamine pyrophosphate-dependent enzymes that catalyze the transfer of a glycolaldehyde from a ketose donor to an aldose as acceptor, and vice versa.^118−120^ Evolved variants of TKs from *E. coli* increasingly accept aliphatic, cyclic, and aromatic substrates.^121^ One of these variants (S385Y/D469T/R520Q) was engineered to convert aromatic aldehydes not accepted by the WT. Tyrosine position 385 was found to be particularly important for aromatic substrate binding. To vary the ring electron density, Wilkinson and Dalby incorporated the ncAAs ***p*AmF**, ***p*CNF**, and ***p*NF** at position Y385 *via* SCS. A 43-fold increase in specific activity was measured for the 385***p*CNF** variant. For 385***p*AmF** and 385***p*NF**, a 13-fold and 4-fold increase in activity, respectively, was determined. Furthermore, ***p*AmF** increased the catalytic efficiency, *k*_cat_/*K*_M_, by 240% and ***p*CNF** by 110%. For ***p*CNF**, a 100% increase in *k*_cat_, and for ***p*NF**, a 290% increase in *K*_M_ were found compared to Y385. In addition, the ***p*AmF** variant demonstrated reduced substrate inhibition compared to Y385.^122^

**Figure 3:**
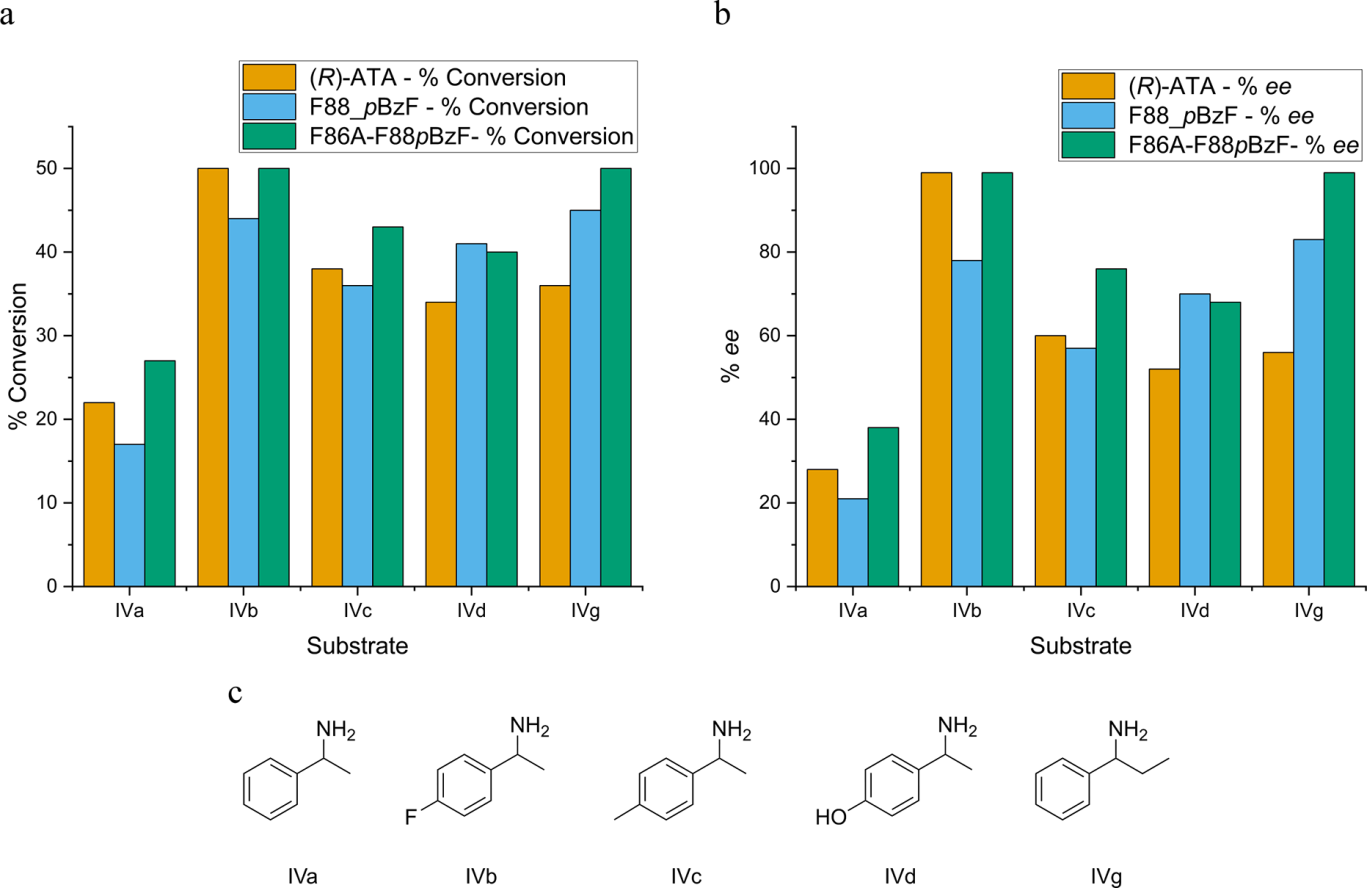
Graphical representation of conversions and % *ee* for R-ATA and variants. This figure presents the conversion (a) and % *ee* (b) of R-ATA and its variants, F88_***p*BzF** and F86A-F88***p*BzF**, toward several amino donors. The results for R-ATA, F88_***p*BzF**, and F86A-F88***p*BzF** are represented in orange, blue, and green, respectively. The *y*-axis in (a) shows the conversion, while in (b) it shows the % *ee*. The *x*-axis in both (a) and (b) displays the tested amino donors, which are depicted in (c) as follows: IVa, 1-phenylethan-1-amine; IVb, *p*-fluorophenylethan-1-amine; IVc, 1-(*p*-tolyl)ethan-1-amine; IVd, 4-(1-aminoethyl)phenol; IVg, 1-phenylpropan-1-amine. The data presented is based on the research conducted by Pagar et al.^125^

Another class of transferases are transaminases, which allow the reversible transfer of an amino group from an amino donor, such as an amine or amino acid, to an amino acceptor. This acceptor is typically a ketone, aldehyde, or keto acid.^123,124^ Pagar et al. aimed to modulate the hydrophobicity of the active center of a (*R*)-amine transaminase (R-ATA) by incorporating *p*-benzoylphenylalanine (**29**, ***p*BzF**), 2,3,4-trifluorophenylalanine (**30**, **(2,3,4-F**_**3**_**)F**), ***p*tFMeF** and *p*-methylphenylalanine (**31**, ***p*MeF**). Incorporation was performed at positions F31, F86, and F88 *via* SCS. No activity was detected for F31 mutants. Incorporation at F86 had an apparent adverse effect with ***p*MeF** and ***p*tFMeF** (reduction of activity by 50% and 61%, respectively) and no effect on the enzyme activity when using ***p*BzF**. However, incorporation at position F88 increased activity for each of the three incorporated ncAAs up to a factor of 3 for F88***p*BzF**, albeit with a limited substrate scope. Further engineering resulted in the variant F86A/F88pBzF, which showed an activity like the WT transaminase. Substrate specificity for various commercially available amino donors and acceptors was tested for R-ATA, F88***p*BzF**, and F86A/F88***p*BzF**, respectively. The constant or increased conversion was observed for all amino donors in the case of F86A/F88***p*BzF**, and an *ee* of 99% was achieved in half of the cases, demonstrating a positive influence on conversion and *ee* due to ncAA incorporation (Figure 3). Additionally, relative activity was increased for several amino acceptors; for example, the relative activity with benzaldehyde as the acceptor was increased by approximately 8- and 5-fold for F88***p*BzF** and F86A/F88***p*BzF**, respectively.^125^

#### 3.1.2 Hydrolases

Merkel et al. demonstrated the parallel, global incorporation of the three fluorinated amino acids 4(*S*)-fluoroproline (**32**, **(4(*S*)-F)P**), ***p*FF**, and 6-fluorotryptophan (**33**, **(6-F)W**) into the lipase from *Thermoanaerobacter thermohydrosulfuricus* (TTL) *via* SPI. These amino acids were targeted for substitution with ncAAs because they all play a fundamental role in lipase activity. TTL natively contains two tryptophan, six proline, and 16 phenylalanine amino acids, representing approximately 10% of the primary sequence of TTL. Contrary to the assumption that this changes the secondary structure of TTL, only small, local perturbations were detected by circular dichroism spectroscopy. While the native lipase had its maximum activity at 70 °C, an optimum at 60 °C was found for the fluorinated lipase. However, the maximum activity at 60 °C was only 60% of the maximum activity of the native enzyme. Considering the number of amino acids exchanged, the measured activity is still high, but it shows that there is still room for improvement in activity.^126^ In another study, Hoesl et al. also looked at TTL. They screened several ncAAs for improving enzyme properties. Therefore, they used azidohomoalanine **(34**, **Aha**), norleucine (**35**, **Nle**), *trans*-4-hydroxyproline (**36**, ***t*****HP**), *cis*-4-hydroxyproline (**37**, ***c*****HP**), and the fluorinated amino acids ***m*FF**, ***p*FF**, ***m*FY**, and ***o*FY** for the global incorporation in different auxotrophic *E. coli* strains using SPI. Thermal stability assays showed reduced activity compared to the WT for all mutants except TTL_***m*FF**. This mutation also exhibited a specific activity after thermal activation that was approximately 25% higher than the native activity. In contrast, the ***p*FF** variant of the same enzyme showed only 40% of the specific activity of the WT. Remarkably, incorporating **Nle** instead of methionine showed activity without thermal activation, whereas the WT was almost inactive. Hoesl et al. attributed this to global conformational changes in the lipase due to the incorporation of **Nle**. Regarding substrate tolerance, native TTL showed the highest activity for tricaproin (C6) and tricaprylin (C8). The above-mentioned mutant TTL_***m*FF** showed a broader spectrum of tricaporins, even converting those with a shorter chain length.^127^ In a further study on TTL, Haernvall et al. investigated the activity of TTL_**Nle** on synthetic polyesters to optimize the efficiency of recycling processes. In this approach, **Nle** was incorporated in TTL *via* SPI. The bis-(benzoyloxyethyl) terephthalate substrate model was used for determining activity. TTL_**Nle** showed an apparent positive influence and achieved approximately 30% higher amounts of hydrolyzed products than the native enzyme. However, the modification did not result in a different pattern of hydrolyzed products. Benzoic acid was the most abundant product, followed by mono-(2-hydroxymethyl) terephthalic acid and hydroxyethyl benzoate. Further analysis of the hydrolysis behavior of TTL_**Nle** toward TTL was carried out using ionic phthalic acid polymers and model substrates containing ether diol. C5, C8, and C12 alkyl diols were tested for the former. Concerning C5 substrates, TTL_**Nle** showed an increased activity of about 5% compared to TTL. Polyesters consisting of ethylene glycols (EG) EG2, EG3, and EG4 were used as model substrates to further test the degradation of plastics. TTL_**Nle** showed an increase in activity of about 40% toward the EGs. The research of Haernvall et al. thus represents an essential step toward recycling synthetic polymers using ncAA.^128^

In an exciting approach, the individual and simultaneous use of SPI and SCS for incorporating the amino acids ***p*BzF** and **Nle** into TTL was evaluated. An activity-based assay demonstrated the highest activity for the variant TTL_**Nle**, which was more than twice as high as the activity of TTL. The construct TTL_D221***p*BzF**_**Nle** showed the second most increased activity, which was significantly reduced but higher than that of WT, TTL_D221***p*BzF**, with the third highest activity. This demonstrated the strong positive impact of the global incorporation of **Nle** into TTL and the moderately positive effect on activity when ***p*BzF** was incorporated at position D221. Despite the reduced activity of the TTL_D221***p*BzF**_**Nle** variant compared to the one with global **Nle** incorporation, it offered the additional advantage of enabling photocrosslinking due to the properties of ***p*BzF**. Hoesl and Budisa were thus able to demonstrate the positive effects of the combination of SCS and SPI.^55^

Phosphotriesterases (PTEs) are membrane-associated metal-dependent enzymes that catalyze the hydrolysis of a wide array of phosphotriesters, phosphodiesters, and phosphonates.^129^ Therefore, they are used in the remediation of plasticizers, petroleum derivatives, and other pesticides.^130,131^ Mechanistically, PTEs execute the P-O bond hydrolysis by using metal-activated water.^132^ Yet, the rate-limiting step is related to the product release stage rather than the bond cleavage.^133^ In order to further improve the *A. radiobacter* PTE activity, Han, Wang and collaborators^134^ focused on the use of *p*-selenolphenylalanine (**38**, ***p*SeHF**) to promote the release of the product by electrostatic repulsion at neutral pH, as this residue bears a low p*K*_a_ side chain (p*K*_a_ = 5.9). Incorporation was performed at Y309 using SCS. The hydrolase activity of the constructed enzyme, *ar*PTE_Y309***p*SeHF**, when using paraoxon at pH 7, was 12-fold higher than that of the parent enzyme. Additionally, the *k*_cat_/*K*_M_ increased by 3.2-fold, suggesting that the presence of ***p*SeHF** strongly facilitates the product-release step. Computational studies indicated that the Y309***p*SeHF** mutation significantly opens the product release gate of the pocket because residue ***p*SeHF** 309 can swing around in a hydrogen-bond network. These results are in line with the previous work from Ugwumba et al., who reached similar conclusions for the hydrolysis of paraoxon at pH 8.5, with *ar*PTE displaying the ncAAs L-(7-hydroxycoumarin-4-yl)ethylglycine (**39**, **Hco**) and L-(7-methylcoumarin-4-yl)ethylglycine (**40**, **Mco**) at position Y309, obtained through SCS.^135^

β-Lactamases play a crucial role in conferring resistance to beta-lactam antibiotics, such as penicillin and cephalosporins, commonly used to treat bacterial infections. These enzymes act by hydrolyzing the β-lactam ring of these antibiotics, ultimately resulting in the antibiotic’s inactivation. This presents a significant challenge to the effective treatment of bacterial infections, as it can lead to the development of antibiotic-resistant strains of bacteria.^136^ Xiao et al. screened 144 of the 286 residues of TEM-1 β-lactamase, to investigate the effect on catalytic efficiency of incorporation of 10 different ncAAs. Their objective was to investigate whether the enhancement of these β-lactamases could provide a competitive edge over organisms that produce β-lactam antibiotics. The following ncAAs derived from phenylalanine and tyrosine were selected and were incorporated *via* SCS: *p*-acetyl-phenylalanine (**41**, ***p*AcF**), ***p*MeOF**, *p*-acrylamidophenylalanine (**42**, ***p*AcrF**), *p*-azido-phenylalanine (**43**, ***p*AzF**), *o*-allyltyrosine (**44**, ***o*AllylY**), *p*-bromophenylalanine (**45**, ***p*BrF**), *p*-iodophenylalanine (**46**, ***p*IF**), *p*-azidomethylphenylalanine (**47**, ***p*AzMeF**), ***p*BiA** and ***ot*BuY**. In a first screening against the β-lactam antibiotic ceftazidime, Xiao et al. found a minimum inhibitory concentration (MIC) of 14 μg mL^–1^ in the case of D179_***p*AzMeF** compared to 0.25 μg mL^–1^ for WT β-lactamase. However, since replacing D179 with cAAs also resulted in an increased MIC, Xiao et al. continued to search for a mutation whose improved catalytic activity could be enhanced by a ncAA alone. Further screening identified the position V216, where the ***p*AcrF** mutant significantly increased the MIC for cephalexin compared to WT (90 μg mL^–1^ compared to 10 μg mL^–1^). To exclude that this could also be achieved with a cAA, the position was screened with all other 19 cAAs, and a beneficial effect of the V216I mutant (20 μg mL^–1^) was detected. A significantly increased *k*_cat_ was found for V216***p*AcrF** (128 s^–1^) compared to WT (9.0 s^–1^) and V216I (40.7 s^–1^). An improved *K*_M_ compared to the best mutant with cAAs for V216***p*AcrF** versus V216I (3375 μM to 6547 μM) was determined. This results in an improvement in *k*_cat_/*K*_M_ of almost an order of magnitude (V216***p*AcrF**: 0.040 μM^–1^ s^–1^; V216I: 0.006 μM^–1^ s^–1^; WT: 0.005 μM^–1^ s^–1^). Thus, Xiao et al. developed a ncAA containing mutant that has an activity that is superior to all variants containing cAAs.^137^

**Figure 4:**
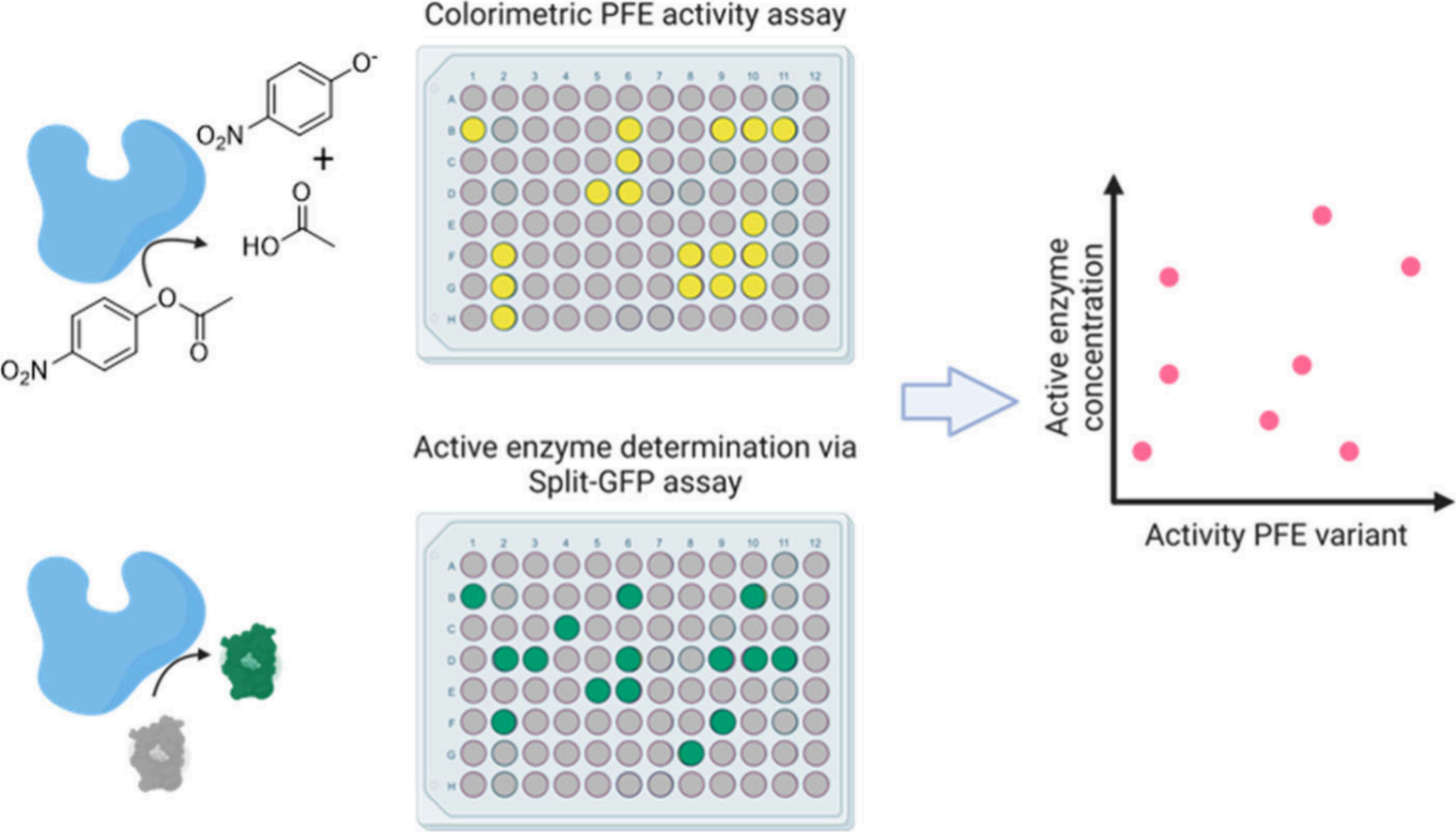
Scatter Plot of Combined Activity and Split-GFP Assay. For the split-GFP assay, PFE was expressed with a GFP11 Fusion Tag to determine active enzyme concentration. The activity was determined using PFE and the substrate *rac*-ethyl-3-phenylbutyrate in a colorimetric assay. The scatter plot represents the concentration (from the split-GFP assay) on the *y*-axis and the activity (from the colorimetric assay) on the *x*-axis. Part of the figure was created with BioRender.com. Reproduced with permission from ref (138). Copyright 2020 Wiley under CC BY 4.0. https://creativecommons.org/licenses/by/4.0/.

*Pseudomonas fluorescence* esterase (PFE) is an enzyme that catalyzes the hydrolysis of esters. In a screening approach, Drienovská et al. combined a split-GFP assay with a PFE activity test to exclude misfolded variants and variants emitting a fluorescent signal but showing low activity (Figure 4). The ncAAs ***p*BzF**, ***p*CNF**, ***p*AzF**, ***p*AmF**, and **NapA** were selected for the assay. This selection of residues for incorporation was structure-based. The screening showed good expression levels for the variants F198***p*AzF**, F198**NapA**, and I224***p*AzF** and, in the case of the ***p*AzF** variant, specific activities above WT. The other promiscuous variants, F158***p*AzF**, F158**NapA**, and F162**NapA**, were further analyzed for activity and enantioselectivity. A significant increase in optical purity in % *ee* compared to WT (27% *ee*) was achieved for the variants F198***p*AzF** (58% *ee*) and F162**NapA** (68% *ee*). These two variants also recorded the highest E values (4.4 and 5.8, respectively), compared to WT (E: 2.3). Also noteworthy is the switch in enantiopreference from (*R*) to (*S*) for the I224***p*AzF** variant. Thus, Drienovská et al. were able to not only increase the catalytic activity but also switch the enantioselectivity by screening and incorporating ncAAs.^138^

#### 3.1.3 Oxidoreductases

**Figure 5:**
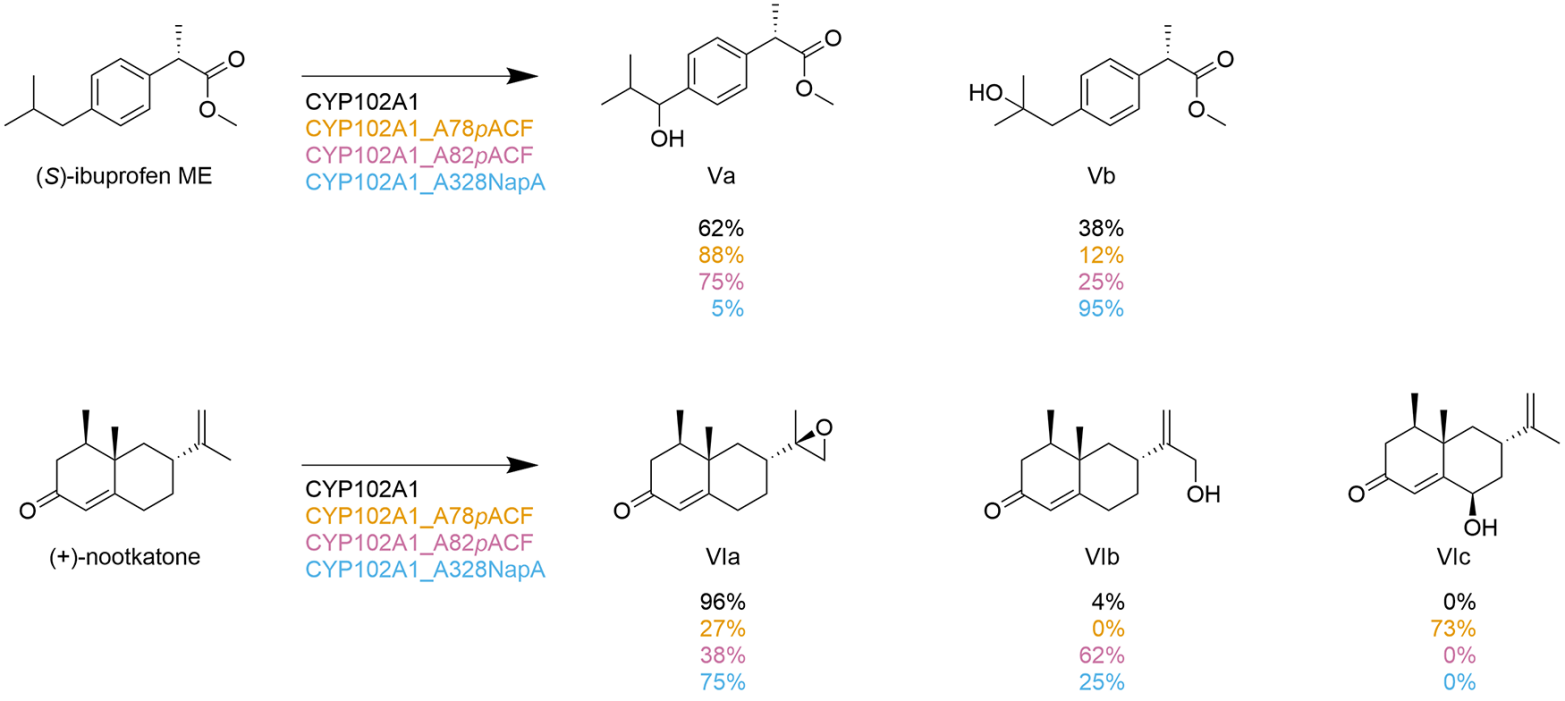
Altered regioselectivity for P40 BM3 and selected variants. This figure illustrates the oxidation of (*S*)-ibuprofen ME and (+)-nootkatone by CYP102A1 (black), Cyp102A1_A78***p*ACF** (yellow), Cyp102A1_A82***p*ACF** (purple), and Cyp102A1_A328**NapA** (blue), leading to the production of Va, Vb, VIa, VIb, and VIc. The variants and results are color-coded for clarity. The data presented is based on the research conducted by Kolev et al.^142^

P450 BM3 peroxygenases are a subclass of oxidoreductases that catalyze the monooxygenation of a wide range of organic molecules under mild conditions. Unlike most P450 enzymes, these enzymes use H_2_O_2_ to catalyze the hydroxylation (peroxygenation) of long-chain fatty acids.^139^ P450 BM3 peroxygenases have shown great potential in biotechnology. Among other exciting applications of P450 are the regioselective hydroxylation of androstenedione, dehydroepiandrosterone, and testosterone.^140^ Cirino et al. globally replaced 13 methionine residues in cytochrome P450 BM-3 TH-4 with the analogue **Nle***via* SPI. Peroxygenase activity assays showed an almost 2-fold higher activity of TH-4_**Nle** compared to TH-4. Partial (“mixed”) incorporation also showed a favorable influence but at a lower level than that of TH-4_**Nle**.^141^ Additional findings reveal altered regioselectivity for (*S*)-ibuprofen methyl ester (ME) and the natural product (+)-nootkatone for the long-chain fatty acid monooxygenase CYP102A1 (P450_BM3_ from *Bacillus megaterium*). Based on the crystal structure, eleven promising positions for ncAA substitution in the active site were identified. The ncAAs for incorporation were selected based on the change in the size of the aromatic side chain and the H-bonding properties of the aromatic ring functional group. The following library of ncAAs was incorporated *via* SCS: ***p*AmF, ***p***AcF**, (*S*)-2-amino-3-(4-(benzyloxy)phenyl)propanoic acid (**48**, **OBnY**), and **NapA** at the promising positions mentioned above. CYP102A1 natively catalyzes the reaction of (*S*)-ibuprofen ME to a benzylic alcohol and a tertiary alcohol (Va:Vb, Figure 5). For the natural product (+)-nootkatone, CYP102A1 has a high regioselectivity of 96% for an epoxide (VIa). Allylic alcohol (VIb) is also formed at 4%. In an initial screening, the most promising variants were A78***p*AcF**, A82***p*AcF**, and A328**NapA**, which were then characterized in more detail. Concerning the (*S*)-ibuprofen ME substrate, Ala328**NapA** demonstrated a regioselectivity of 95% for Vb and A78***p*AcF** with 88% for Va. For (+)-nootkatones, the Ala82***p*AcF** variant showed a significantly increased regioselectivity of 62% compared to 4% of the WT for VIb. The Ala78***p*AcF** variant showed 73% regioselectivity for an allylic alcohol (VIc) not formed by CYP102A1, demonstrating the possibility of obtaining new products when using ncAA containing enzymes (Figure 5).^142^

Reductases are a subclass of oxidoreductases that catalyze the reduction of various substrates, often using nicotinamide cofactors as reducing agents.^143^ In a pioneering study, Jackson et al. incorporated eight ncAAs into *E. coli* nitroreductase (NTR), an enzyme that can function as a prodrug activator for oncological treatment. In the first generation, ***p*AmF**, **NapA**, ***p*BzF**, and ***p*MeOF** were incorporated *via* SCS. Subsequently, in the second generation, *p*-aminomethylphenylalanine (**49**, ***p*AMMeF**), ***p*MeF**, ***pt*****FMeF**, and ***p*NF** were also incorporated *via* SCS. The researchers chose position F124 for incorporation due to its known crucial role in substrate binding. In the first generation, ***p*AmF** demonstrated an apparent positive influence on the catalytic efficiency (*k*_cat_*/K*_M_) concerning the prodrugs CB1954 and LH7. In the second generation, the incorporation of the *p*-nitro group of ***p*NF** led to a significant enhancement in catalytic efficiency. This resulted in a 30-fold increase in catalytic efficiency (NTR_***p*NF**) compared to WT and a 2.3-fold increase compared to the best mutant with a cAA.^144^ Zheng and Kwon focused on another reductase. They selectively reduced the binding affinity of murine dihydrofolate reductase (mDHFR) to the inhibitor methotrexate without reducing its binding affinity to the natural substrate dihydrofolate (DHF). Therefore, **NapA** and ***p*BrF** were incorporated at position F31 to shift the selectivity of the enzyme toward DHF. The incorporation was performed *via* SCS and additionally using an auxotrophic expression strain and minimal media.^145^ This method of incorporation was chosen to reduce the misincorporation with cAAs.^146^ mDHFR_***p*BrF** and mDHFR_**NapA** demonstrated an increased binding affinity for DHF over methotrexate, with a 4.0- and 5.8-fold increase, respectively. This enhanced selectivity was attributed to the decreased binding affinity to inhibitor methotrexate, which was found to be 2.1-fold and 4.3-fold lower, respectively.^145^ In a subsequent study, Zheng et al. manipulated the substrate specificity of mDHFR from DHF to folate by incorporating **NapA.** The incorporation was performed *via* SCS while also using an auxotrophic strain and minimal media. *RosettaLigand* and *RosettaDesign* were used to identify position F31 as optimal for incorporation. MDHFR_**NapA** demonstrated a significantly reduced *K*_M_ for FOL (9.5 μM) compared to WT (22.5 μM for FOL). However, *K*_M_ was lower for DHF in the case of WT and mutant (6.5 μM and 4.8 μM, respectively). Concerning *k*_cat_/*K*_M_, mDHFR_**NapA** showed 7.6-fold higher relative enzymatic activity than WT.^147^

Ascorbate peroxidase is a heme enzyme that scavenges reactive oxygen species (ROS) and catalyzes the decomposition of H_2_O_2_ to prevent oxidative damage.^148^ To elucidate the importance of the aspartate-histidine-hydrogen bond, Green et al. incorporated **NMH** into an engineered ascorbate peroxidase (APX2). The replacement of proximal H163 *via* SCS for **NMH** not only revealed the role of the conserved interaction between H163 and D208 but also resulted in a mutant with a 5-fold increase in total turnover number (TON). Differences in TON of APX2_**NMH** for the respective phenolic substrates were revealed, showing the highest increase in TON compared to APX2 for guaiacol (5-fold). Further, most of the tested substrates demonstrated a substantial increase in TON (2-methoxyaniline (2-fold), *o*-cresol (4-fold), phenol (1.9-fold), and 3,5-dimethylphenol (1-fold). The TON of APX2_**NMH** can be partially attributed to the fact that it undergoes less inactivation during catalysis, as evidenced by the analysis of kinetic parameters. Specifically, Green et al. found that APX2 had a *k*_cat_*/K*_M_ of 86 mM^–1^ s^–1^ and APX2_**NMH** had a *k*_cat_*/K*_M_ of 110 mM^–1^ s^–1^, demonstrating the improvement of the variant. The TON was increased from 6200 (APX2) to 31300 (APX2_**NMH**) respectively. They further identified the H163–D208 interaction and found that mutating D208 in APX2_**NMH** did not result in a significant TON loss, unlike for APX2. This was attributed to the fact that the tautomeric form of the imidazole ring was fixed, and the neutral charge of the proximal ligand was not affected during the catalytic cycle.^149^

Alcohol dehydrogenases reversibly convert alcohols into aldehydes or ketones. This is achieved by moving a C4-hydride from NAD(P)H to the carbonyl carbon present in an aldehyde or ketone substrate.^150^ In the case of *Zymomonas mobilis*, its alcohol dehydrogenases are dependent on metals: alcohol dehydrogenase I (ADH1) relies on zinc, while alcohol dehydrogenase II (ADH2) requires iron.^151^ Notably, ADH2 becomes inactive in the presence of oxygen, unlike ADH1. Bhagat et al. aimed to increase the enzyme’s tolerance to reactive oxygen species by switching the metal atom in the metal-binding site from iron to zinc. This modification was part of their broader goal to produce biofuel under aerobic conditions in photosynthetic organisms, using photosynthesis to generate ethanol with ADH2. However, ADH2 is deactivated by the oxygen produced during photosynthesis. To overcome this, they incorporated the ncAA *L*-3,4-dihydroxyphenylalanine **(50, *L*-DOPA)** at position H277 to modify the metal-binding site. This resulted in the mutant ADH2_***L*-DOPA**, which showed a higher affinity for Zn^II^ over Fe^II^ under aerobic conditions. Moreover, ADH2_***L*-DOPA** remained active longer under oxygenic conditions than WT. This demonstrated an improved selectivity for a different metal ion, which is presumably the reason for its extended functionality under oxidative conditions.^152^

#### 3.2 Influencing Stability

Most enzymes are only effective and stable under specific conditions, reducing (losing) their activity or denaturing outside this range. Therefore, the stability of enzymes is a critical factor in many applications such as for industrial processes requiring or withstanding different conditions such as high temperatures, high pressures, pH fluctuations, organic solvents, etc.^143,144^ However, searching for a more stable enzyme is not straightforward: facing multiple challenges as a common tradeoff between stability and activity is known.^149^ This section discusses the use of ncAAs to aid the design of more stable natural enzymes. For a more detailed discussion regarding thermostability, the reader is encouraged to view a previous review.^153^

A popular approach to stabilizing enzymes using ncAAs has focused on the use of fluorinated ncAAs,^153−156^ which allow the formation of stabilizing interactions, such as halogen bonds, within the enzyme. Fluorinated ncAAs were especially used in early strategies employing SPI. Although many of these approaches did not yield a positive effect, examples of remarkable improvements exist. For instance, the half-life of chloramphenicol acetyltransferase (CAT) improved by a factor of 27, and its thermostability increased by 9 °C after globally replacing leucine with 5′,5′,5′-trifluoro-leucine (**TFL, 51**).^157^ Improvements in pH-stability were also achieved – for example the incorporation of ***m*FY** in β-galactosidase resulted in a 2- to 4.5-fold higher activity at an altered pH.^158^ In another example, multiple enzyme characteristics (thermostability, organic solvent stability, and pH-stability) of ω-transaminase (ω-TA) were improved after globally incorporating ***m*FY**.^159^ Another example was reported by Budisa and colleagues, who tested a broad array of ncAAs, including fluorinated ones, in the presence of solvents, surfactants, reducing agents, alkylating agents, denaturing agents, and inhibitors. The global incorporation of ***m*FF** in TTL led to a 0.7-fold increase in activity following treatment with guanidinium chloride, while the WT was inactivated. Additionally, they demonstrated activity in the presence of the protein inhibitor pefabloc with this variant.^160^ While many studies have focused on fluorinated ncAAs, other halogenated amino acids are demonstrating their potential as alternatives for increasing enzymatic stability. These are particularly used in more recent attempts employing SCS. For example, the incorporation of ***m*ClY** in GST resulted in 79% of activity retained after heat treatment, while the WT lost all activity.^153^ The incorporation of the same ncAA in microbial transglutaminase (MTG) showed a residual activity of 46% compared to a nearly total loss of activity in the WT.^154^

Another strategy involves introducing new chemical groups, which allow the formation of different covalent bonds, resulting in functionalities such as hemithioketal, thiourea, and thioether. Incorporating ***p*BzF** into O-succinyltransferase (metA) via SCS resulted in a 21 °C increase in T_m_, presumably due to the formation of a hemithioketal.^161^ An additional example of the benefit of introducing new covalent bonds was the incorporation of an isothiocyanate phenylalanine variant into metA, forming a thiourea that cross-linked monomers, resulting in a 24 °C higher T_m_ than that of the WT.^162^ Furthermore, introducing a bromoethyl tyrosine variant into pullulanase increased the T_m_ by 7 °C, which is attributed to thioether linkages between cysteines and the halogenated ncAA.^163^

ncAAs allow for more precise control when immobilizing enzymes, another strategy for increasing stability.^164−166^ A notable example is the incorporation of ***p*AzF** into aldehyde ketone reductase via SCS. Five-point immobilization on a resin led to approximately 70% activity retention after incubation at 70 °C. Furthermore, the half-life at elevated temperatures was seven times longer than that of the WT.^165^ Thus, using SCS, specific sites can be chosen for linking to a resin, aiming to avoid a negative impact on the active site and allowing for a specific orientation.

Table 1 provides a comprehensive overview of ncAAs that have been successfully incorporated into natural enzymes. The examples in the table are organized by enzyme class, including transferases, hydrolases, and oxidoreductases, as well as by the method of incorporation, namely SPI and SCS. This arrangement facilitates a swift and clear understanding of the different strategies employed in these studies.

**Table 1:** Comprehensive Overview of the Strategies Used to Improve Enzyme Stability through the Use of ncAAs^a^

Enz class & Incorp meth	Enzyme	Incorporated ncAA(s)	AA(s) replaced	Effect on	Outcome	Ref
**Transferase - SPI**	Adenylate kinase (ADK)	**Nle**	M	Oxidant	A higher tolerance toward inactivation in presence of H_2_O_2_ was observed.	(167)
	CAT	**TFL**	L	Thermostability & Half-life	After thermal incubation, the half-life dwindled from 160 to 6 min, but a 2-fold increase in secondary structure stabilization was detected. Structural destabilization at elevated temperatures was observed.	(168)
	CAT_L158I	**TFL**	L	Thermostability	The L158I mutation showed that the incorporation of **TFL** at this position had a negative effect on the enzyme’s thermostability.	(169)
	CAT	**TFL**	L	Thermostability & Half-life	A 27-fold enhancement in stability (t_1/2_) and 9 °C in temperature tolerance was demonstrated.	(157)
	HAT	***o*FF**, ***m*FF**, ***p*FF**	F	Thermostability	HAT_***o*FF**, HAT_***m*FF**, and HAT_***p*FF**, showed a loss of secondary structure. Losses of 5.1, 2.5, 2.1 °C of melting temperature (T_m_), respectively, were observed.	(78)
	HAT PCAF	***o*FF**, ***m*FF**, ***p*FF**	F	Thermostability	A 5 K decrease of T_m_ for PCAF_***p*FF** and 2 K for PCAF_***m*FF** were detected. PCAF_***o*FF** showed a complete structure disruption.	(77)
	*KlenTaq* DNA polymerase	**Trifluoromethionine** (**52**, **TFM**)	M	Thermostability	More than 50% decrease of activity after heat treatment for *KlenTaq*_**TFM**. WT *KlenTaq* retained 90% activity.	(170)
	*KlenTaq* DNA polymerase	**(4*****R*****)fluoroproline** (**53**, **(4(*****R*****)-F)P**)	P	Thermostability	*KlenTaq*_**4(***R***)-F)P** lost 50% of the original activity after heat treatment. Comparison to WT (see above).	(171)
	ω-TA	***m*FY**	P, Y, W	Thermostability & Solvent stability and pH	ω-TA_***m*FY** retained 36% of its initial activity after heat treatment, while WT showed 3.3% activity. ω-TA_***m*FY** variant’s half-life was 2.3 times higher than WT. ω-TA_***m*FY** exhibited increased stability in the presence of DMSO. At all tested pHs, the variant showed slightly higher activity at varied pH.	(159)
	Gaussia luciferase	**Aha**, homopropargyl-glycine **(54**, **HPG**)	M	Fluorescence emission	Prolonged light emission with *t*_1/2_ = 3.8 min, an almost 3-fold improvement compared to WT.	(170)
						
**Transferase - SPI + SCS**	ω-TA	**(4*R*-)FP**, ***L*-DOPA**	P, R	Thermostability & Solvent stability	ω-TA_ DOPA_**(4***R***-)FP** showed no adverse effect on secondary structure. ω-TA_**(4***R***)-FP** and ω-TA_**DOPA**_**(4***R***)-FP** showed T_m_ at 77 and 89 °C. ω-TA_**DOPA** demonstrated similar T_m_ to WT, which were 65 and 74 °C, respectively.	(172)
						
**Hydrolase - SPI**	β-galactosidase	***m*FY**	Y	pH Stability	At pH 7.0, β-galactosidase_***m*FY** showed a V_max_ for various substrates 2 to 4.5 times higher than WT.	(173)
	H31N-H137N lambda lysozyme (λL)	1,2,4-triazole-3-alanine (**55**, **TAA**)	H	pH Stability	At neutral pH, λL_**TAA** was 3.5 kcal/mol less stable than WT. This was attributed to that protonation of the imidazole ring of His48-λL was not favored.	(174)
	*Pvu*II endonuclease	***m*****FF**, ***p*FF**	F	Conformational stability	The conformational stability of *Pvu*II_***m*FF** was indistinguishable from WT. A 1.5 kcal/mol loss in conformational stability was detected for *Pvu*II_***p*FF**.	(104)
	Pancreas phospholipase A2 (PLA2)	**TAA**	H	pH stability	PLA2 demonstrated no reduction in activity at varied pH. No activity was measured for WT at pH 3.	(175)
	Organophosphate hydrolase (OPH)	***m*FY**	Y	pH stability & Thermostability	OPH_***m*FY** exhibited catalytic activity in a larger pH range 5.5–12.0 (WT: 7.0–12.0). Regarding pH, OPH_***m*FY** was almost unaffected in its activity and was still 20% active at 70 °C at pH 8.5, whereas OPH showed only 10%.	(176)
	CalB N74D	**5FW**, ***m*FY**, and ***p*FF**	W, Y, F	Secondary structure & Shelf life	A slight decrease in stability was observed for calB_***p*FF**. Significant reduction in secondary structure was detected for calB_**5FW**. Tyrosine substitution increased coiled-coil content. After several months all fluorinated variants showed increased activity compared to WT.	(177)
	TTL	**Aha**, **Nle**, ***m*FY**, *****o***FY**, ***m*FF**, ***p*FF**, *t***HP**, ***c*****HP**, and **4(*****S*****)FP**, **4(*****R*****)FP**	M, P, F, Y	pH stability & Thermostability	Altered pH optima and thermostability were observed for variants. Despite the broad spectrum of incorporated ncAAs, none exceeded the thermostability of the WT.	(127)
	TTL	4-amino-tryptophan (**56**, **4AmW**), 4-fluoro-tryptophan (**57**, **4FW**), 7-aza-tryptophan (**58**, **7AzW**), **4(*****S*****)FP**, **4(*****R*****)FP**, ***c*****HP**, ***t*****HP**, ***o*FY**, ***m*FY**, **Nle**, **Aha**, ***m*FF** and ***p*FF**	W, P, F, Y, M	Solvent stability, surfactants, reducing-, alkylating- and denaturing agents and inhibitors	Increases and decreases in solvent stability were detected. At Incubation with the surfactant, CHAPS TTL_***t*****4HP** showed 16.3-fold higher activity. At treatment with guanidinium chloride, TTL_**4AmW/*m*FF** variants showed a 0.7-fold increase in activity, while WT was completely inactivated. In the presence of the protein inhibitor Pefabloc, TTL_***t*****4FP/*m*FY** showed 0.4-fold activity.	(160)
	S5 PTE	***p*FF**	F	Thermostability	After heat treatment of PTE_***p*FF** a CD signal of 30–33% was recovered, while the WT́s CD signal was completely lost. The T_m_ of PTE_***p*FF** was approximately 2 °C higher than WT.	(178)
	S5 PTE_F104A	***p*FF**	F	Thermostability & Shelf life	F104A PTE_***p*FF** exhibited 50% of its initial activity after heat treatment, whereas WT exhibited 24% of its initial activity. PTE_***p*FF** showed a longer shelf life, retaining 66% of its initial activity after seven days, whereas WT had less than 50% of its initial activity after three days.	(179)
						
**Transferase& Oxidoreductase - SPI**	ω-transaminases (ω-Tas1 and ω-Tas2) and alanine dehydrogenase	***o*FY**, ***m*FY**, **(2,3-F**_**2**_**)Y**, and **(3,5-F**_**2**_**)Y**	Y	Thermostability	For all three enzymes, the variants containing ***o*FY** showed increased T_m_ of ∼4 °C and higher residual activity upon incubation at high temperatures compared to WT.	(155)
						
**Oxidoreductase - SPI**	P450 BM3 TH-4	**Nle**	M	Thermostability & Solvent stability	Increased stability toward DMSO. Complete loss of activity after heat treatment.	(141)
						
**Transferase -SCS**	GST and Azoreductase	*m*-Iodo-tyrosine (**59**, ***m*IY**), *m*-bromotyrosine (**60**, ***m*BrY**), ***m*ClY**	Multiple positions	Thermostability	GST_***m*ClY** (−1, 22, 32, 57, 73, 141, 163) retained 79% activity after heat treatment, while WT lost activity. For azoreductase, the ***m*BrY** variant (108, 156, 179) showed 13-fold increased half-life compared to WT.	(153)
	MTG	***m*ClY**, ***m*IY**, ***m*BrY**, N^ε^-allyloxycarbonyl-*L*-lysine (**61**, ***AlocKOH***)	Y20, Y62, Y171	Thermostability	MTG_***m*ClY** retained 46% residual activity upon thermal incubation (WT: 1.8%). 5.1-fold longer half-life compared to WT.	(154)
	metA	***p*AcF**, ***p*BzF**, ***ot*BuY**, ***p*AzF**, ***p*MeOF**, ***p*IF**, ***p*BrF**, *p*-boronophenyl-alanine (**62**, ***p*BoF**), **oAllylY**, ***p*AcrF**, among others	randomly	Thermostability	The T_m_ of the variants metA_F21***p*BzF** and metA_N86***ot*BuY** showed an increase of 21 and 6 °C, respectively compared to WT, due to the formation of a hemithioketal.	(161)
	metA	*p*-isothiocyanate phenylalanine (**63**, ***p*NCSF**)	scanning library	Thermostability	metA_F264***p*NCSF** demonstrated 24 °C higher T_m_ than WT. This was attributed to the formation of thiourea cross-linking monomers through ***p*NCSF** and P2 sites.	(162)
	Transketolase (TK)	***p*AmF**, ***p*CNF** and ***p*NF**	S385	Thermostability	TK1_385***p*AMF** revealed an increased T_m_ of 2.4 °C compared to WT and 4.9 °C to the best cAA mutant (S385Y/D469T/R520Q).	(122)
						
**Hydrolase - SCS**	T4 lysozyme (T4L)	Norvaline (**64**, **Nv**), ethylglycine (**65**, **EtG**), O-methyl serine (**66**, **OMetS**), (2*S*)-2-amino-4-methylhexanoic acid (**67**, **iL**), (2*S*)-amino-3-cyclopentyl-propanoic acid (**68**, **CpA**), *tert*-butylleucine (**69**, ***t*****BuL**)	L133	Thermostability	T4L_L133**CpA** and T4L_L133**iL** showed a 4.3 and 1.9 °C increase in T_m_ compared to T4L_L133.	(180)
	T4L	***m*ClY**, ***m*BrY**, or ***m*IY**	Y18	Thermostability	At an elevated temperature, T4L_Y18***m*ClY** showed a 15% increase in activity compared to WT and an increase of ∼1 °C in T_m_.	(156)
	T4L	*p*-propargyloxy-*l*-phenylalanine (**70**, ***p*PAF**)	L91	Freeze–thaw cycles & Denaturation	Immobilized variant T4L_***p*PaF** retained more activity after denaturing conditions (freeze–thaw cycles and urea) outperforming WT (immobilized and free).	(166)
	Keratinase from *Pseudomonas aeruginosa* (KerPA)	***p*BrF**, ***p*ClF**, ***p*IF**, ***p*MeOF**, ***p*MeF**, (*S*)-2-amino-3-(4-(*tert*-butyl)phenyl)propanoic acid (**71**, ***pt*BuF**), ***pt*FMeF**, ***p*AzF**, ***p*AcF** and ***p*BzF**	Tyrosine positions	Thermostability & Reducing conditions	Triple mutant KerPA_Y21***p*BzF**_Y70***p*BzF_**Y114pBzF showed a higher activity than WT in the 35–80 °C temperature range. Thermostability was higher between 70 and 80 °C compared to WT. The variant was more resistant to reducing conditions at 55 °C.	(181)
	N-terminal truncated β-lactamase (ΔTEM-1)	(*S*)-2-amino-3-(4-(2-mercaptoethoxy)phenyl)propanoic acid **(72**, **SetY**), (*S*)-2-amino-3-(4-(3-mercaptopro-poxy)phenyl)propanoic acid **(73**, **SprY**), (*S*)-2-amino-3-(4-(3-mercaptopropoxy)phenyl)propanoic acid (**74**, **SbuY**)	Random	Thermostability	ΔTEM-1_R65C_A184**SbuY** showed ∼9 °C increasement in T_m_ compared to WT and remained active till 45 °C, while WT was inactive at 40 °C.	(182)
	Pullulanase	O-2-bromoethyl tyrosine (**75**, **BetY**)	A72, T73, 171C	Thermostability	Thioether linkages were used to stabilize pullulanase. Pul_**BetY**_T126F_A72R showed improved stability (T_m_ ∼ 7 °C higher and *t*_1/2_ 211% increase) compared to WT.	(163)
	Keratinase from *Bacillus licheniformis* WHU (KerBL)	(*S*)-2-amino-3-(4-(3-bromopropoxy)phenyl)propanoic acid (**76**, **BprY**) or (*S*)-2-amino-3-(4-(4-bromobutoxy)phenyl)propanoic acid (**77**, **BbtY**)	E53, Y102, and Y260	Thermostability	Proximity-triggered cross-linking was used. KerBL_N159C_Y260**BprY** and KerBL_N159C_Y260**BbtY** were more thermostable under normal and reducing conditions than WT. T_m_ was increased by 10.2 and 11.9 °C, respectively.	(183)
	Carboxylesterase P1 from *Sulfolobus solfataricus* (EST1)	***p*PaF**	Y116	Solvent & Shelf life	Immobilized EST1_***p*PaF** maintained most of its initial activity after six months at RT. WT showed decreased activity upon increased THF concentrations, while the contrary was observed for immobilized EST1_***p*PaF**.	(164)
						
**Oxidoreductase - SCS**	Aldehyde ketone reductase	***p*AzF**	Y110, Y114, Y143, Q162, and Q189	Half-life	***p*AzF** was used for selective immobilization with single or multipoint linking sites. The 5-fold immobilized ***p*AzF** variant retained ∼70% of its initial activity, while free WT lost almost all of it. The half-life of the variant at 60 °C was 45 h, ∼7-fold higher than free WT.	(165)

a Table categorized by enzyme class and incorporation method, enzyme used, ncAA(s) incorporated, amino acid(s) replaced, categorization of the effect on stability, result reported in the publication, and citation.

### 3.3 Discussion and Perspectives on Improving Natural Enzymes via ncAA Incorporation

Initially, SPI was the method of choice for improving enzymes. However, the results from this are difficult to predict. This unpredictability primarily stems from the difficulty in forecasting the effects due to the large number of ncAAs incorporated when using SPI and the varying degrees of efficiency, resulting in the formation of (statistical) mixtures of variants with different degrees of ncAA incorporation. SCS has allowed for improved rational design of ncAA incorporation because only one ncAA is incorporated, which can be done in a more informed manner by relying on existing crystal structures or improved computational models.^135,142,147^

Currently, the toolbox of ncAAs contains a variety of functional groups enabling precise tailoring of the enzyme’s active site. This expanded toolbox makes it possible to change or fine-tune electrostatic interactions by introducing new electron donating or withdrawing groups.^122,134,144,147,184^ Furthermore, it allows for modulation of physical properties such as p*K*_a_-value and hydrophobicity.^125,134,144,149^ Additionally, new structural characteristics can be introduced like bulkier side chains than possible with cAAs and the diversification of aromatic side chains.^142,185^

The most frequently used approach to stabilize an enzyme is the use of halogenated amino acids.^153−160^ Incorporating ncAAs with side chains allowing the formation of thiourea-, hemithioketal- or thioether-groups has shown potential in improving enzymatic stability as well.^161−163^ Moreover, introducing side chains containing azides and alkynes unlocks the use of click-chemistry. This enables the biorthogonal, site-specific immobilization on a resin, contributing to improved enzymatic stability.^164−166^ Taken together, the variety of possible interactions of ncAAs supplements what can be achieved with classical enzyme engineering.

## 4 Enzymatic Assemblies Using Noncanonical Amino Acids

Bio-orthogonal chemistry involves specific reactions that occur selectively and efficiently in biological environments.^186^ This section focuses on discussing different studies in which ncAAs are used as connectors for (multi)enzyme assembly with biocatalytic purposes. Other approaches have been reported for obtaining (multi)enzyme complexes, for example, through self-assembly with metal ions using chelating ncAAs ligands.^187−190^ However, these studies are not further discussed in this section, as, in most cases, their application for biocatalysis has yet to be studied.

Achieving selective and precise assembly of multienzyme complexes is crucial, especially for cascade reactions. Cascade reactions have several advantages, like avoiding separating and purifying intermediates, shifting the reaction equilibrium, and improving yields.^191,192^ Furthermore, bringing enzymes’ active sites closer allows, for example, substrate channeling or protection of unstable intermediates.^193^ Various approaches exist for multienzyme assemblies, including fusion enzymes or covalent coupling using cAAs. However, these methods have limitations, such as only allowing C- and N-terminal links in genetic fusion, generating undesired enzyme mixtures and activity loss. Furthermore, spatial control over complex assemblies is generally limited.^194,195^ Therefore, ncAAs present an intriguing alternative for developing more precise (multi)enzyme assembly complexes compared to traditional methods.

### 4.1 Assembly of Single Enzymes

**Figure 6:**
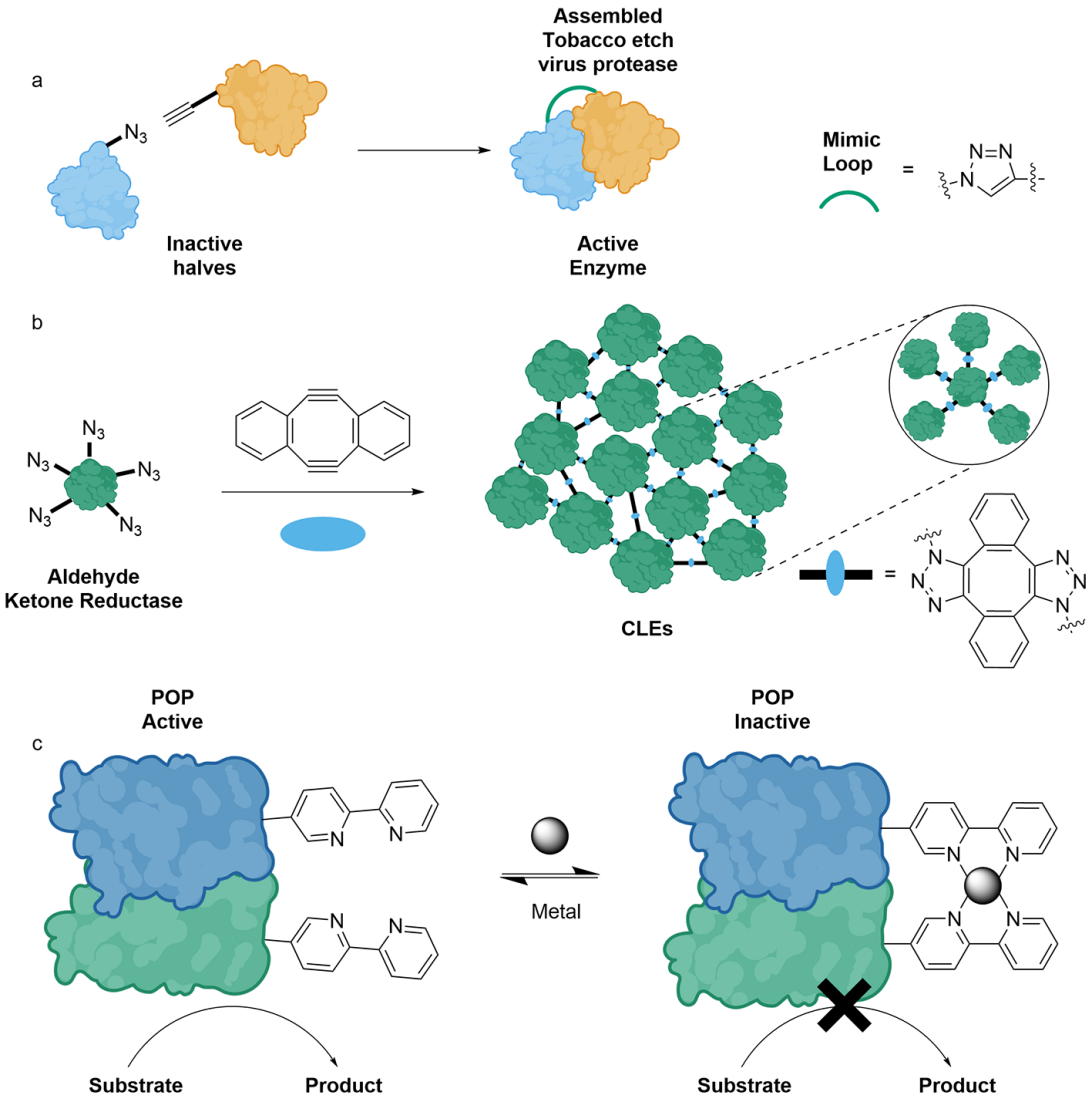
Different strategies for single enzyme assembly aided by ncAAs. (a) Assembly of two inactive protein fragments through bioconjugation to generate an active enzyme with Tobacco etch virus protease as an example. (b) Generation of cross-linked enzyme assembly mediated by linkers using ncAAs as bio-orthogonal handles with aldehyde ketone reductase as an example. (c) Enzyme regulation (switchable enzymes) dependent upon metal binding with prolyl oligopeptidase (POP) as an example. Part of the figure was created with BioRender.com.

Schoffelen’s group used ncAAs to join two inactive peptide fragments to generate a catalytic active enzyme (Figure 6a).^196^ The enzyme under study was tobacco etch virus protease, which was split into two halves at the loop connecting its two domains. One of the halves incorporated an azide-containing ncAA **Aha** or N^ε^-[(2-azidoethoxy)carbonyl]-*L*-lysine (**78**, **AZL**), and the other an alkyne-containing ncAA **HPG** or N^ε^-(propargyloxy)-carbonyl-l-lysine (**79**, **PaL**). The coupling was made through copper-catalyzed azide-alkyne cycloaddition (CuAAC). The most efficient result was observed for the **AZL/PaL** combination with quantitative conversions. The artificial protease linked using ncAAs exhibited similar protease catalysis, *K_M_*, and *k*_cat,_ as the natural tobacco etch virus protease one. Furthermore, the authors explored the use of strain-promoted azide–alkyne cycloaddition (SPAAC). However, replacing **PaL** by N^ε^-[(1*R*,8*S*,9*R*)-bicyclo[6.1.0]non-4-yn-9-ylmethoxy]carbonyl-*L*-lysine (**80**, **BCNK**) rendered almost no conjugate peptides compare to using either **Aha** or **AZL**.^196^

Cross-linked enzyme aggregates (CLEAs) are a promising strategy for enzyme immobilization.^197^ In this sense, Li and colleagues proposed a more precise enzyme cross-linking strategy using ncAAs (Figure 6b). ***p*AzF** was incorporated at multiple sites in aldehyde ketone reductase (AKR), avoiding positions close to the active site.^198^ The enzyme assembly was made using a linker bearing two alkyne moieties. The cross-linked enzyme (CLE) complex could be formed using the cell lysate supernatant, thus avoiding the need for enzyme purification. The most promising CLE (AKR_5_CLE), with five linking sites, presented a catalytic efficiency of 5.26 ± 0.13 mM^–1^·s^–1^, 3.6-fold higher than that of the corresponding free enzyme. Furthermore, AKR_5_CLE was tested for ketone reduction to obtain chiral (*S*)-1-(2,6-dichloro-3-fluorophenyl)ethanol. After 12 h, the product was obtained with a yield of 91% (8.4-fold higher than that of free AKR) and *ee* > 99%. AKR_5_CLE proved to be reusable for six successive cycles of 12 h reaction each, retaining 80% of the initial activity with high *ee* values (>99%) for each cycle.^198^

In another approach, Lewis and co-workers explored a different way to use ncAAs for catalysis by imposing subtle control of enzymes’ conformation.^199^ The authors developed a switchable serine protease prolyl oligopeptidase (POP) that, in the presence of a transition metal (M) ion cannot perform its catalysis while the activity was recovered upon metal removal (Figure 6c). This strategy depended on the enzyme undergoing a dynamic opening/closing of its β-propeller domain to allow entry of the substrate to the active site. Computational analysis was used to find suitable sites for (2,2′-bipyridin-5-yl)alanine (**81**, **BpyA**) incorporation that allows M(BpyA)_2_ arrangement to generate inactive conformations. Four promising variants were found using high throughput screening with almost complete inhibition in presence of a high concentration of Ni^II^ while maintaining WT activity (>75%). Moreover, upon incubation with EDTA, the four POP variants recovered their catalytic activity almost quantitatively. Variant POP_167/517_**BpyA** could undergo 12 on/off cycles in less than 5 min. The same workflow was used for the *Photinus pyralis* luciferase enzyme; variants of the luciferase 202/532_**BpyA** and 108/508_**BpyA** kept WT activity (>25%) while being inhibited by Ni^II^. One challenge for these systems is that metal ions and chelator accumulate after multiple cycles.^199^

Wang’s group developed a catalyst using ADH CLEs (CLEs-TiO_2_-Cp*Rh(bpy)).^200^ This hybrid material can be used to regenerate NADPH, required by ADH for ketone reduction, in a photocatalytic manner. The material was generated in a sequential two-step process. Initially the rhodium complex [Cp*Rh(bpy)H_2_O]^2+^ was mixed with the TiO_2_ nanotubes. Afterward, the TiO_2_-Cp*Rh(bpy) material was combined with ADH CLEs. The CLEs were formed upon incorporating ***p*PaF** at positions Y156 and Y229 in ADH and cross-linked using a bifunctional azide linker through microwave-assisted CuAAC. The hybrid material was used for the asymmetric reduction of 3,5-bis(trifluoromethyl)acetophenone to (*R*)-1-[3,5-bis(trifluoromethyl)phenyl]-ethanol with 41% yield and *ee* > 99%. The material was reused for six cycles, maintaining about 95% of its initial activity.^200^

### 4.2 Multienzyme Assembly

#### 4.2.1 Two Enzymes

The first report of using ncAAs to join two enzymes was by Bundy and Swartz.^201^ For this, ***p*AzF** and ***p*PaF** were incorporated in superfolder GFP (sfGFP) and dihydrofolate reductase (DHFR), respectively. Protein conjugation of DHFR and sfGFP was performed using CuAAC in anaerobic conditions with 43% yield. However, the activity of DHFR was undetectable and significantly reduced for sfGFP, which was most likely related to the presence of copper. The general procedure was optimized to reduce Cu concentration, add a ligand, and remove copper after conjugation to improve the results. This new procedure allowed conjugation of sfGFP_***p*AzF** and sfGFP_***p*PaF** without significant loss of the total sfGFP activity. Afterward, Kim and co-workers proposed a copper free approach for assembling two enzymes.^202^ The enzyme complex was formed by mixing GST incorporating ***p*AzF** and maltose-binding protein containing **BCNK** through SPAAC. ***p*AzF** was introduced at different sites in GST (F46, K87, K113, or H139) and **BCNK** in maltose-binding protein (K29, K83, or Y167), respectively. All possible combinations were tested for bioconjugation. The efficiency differed for each combination, with the highest efficiency obtained when combining GST with ***p*AzF** incorporation at F46 and **BCNK** at K83 of the maltose-binding protein. Using **AZL** instead of ***p*AzF** in GST for two positions gave rise to lower conjugation efficiency under the same conditions.^202^

**Figure 7:**
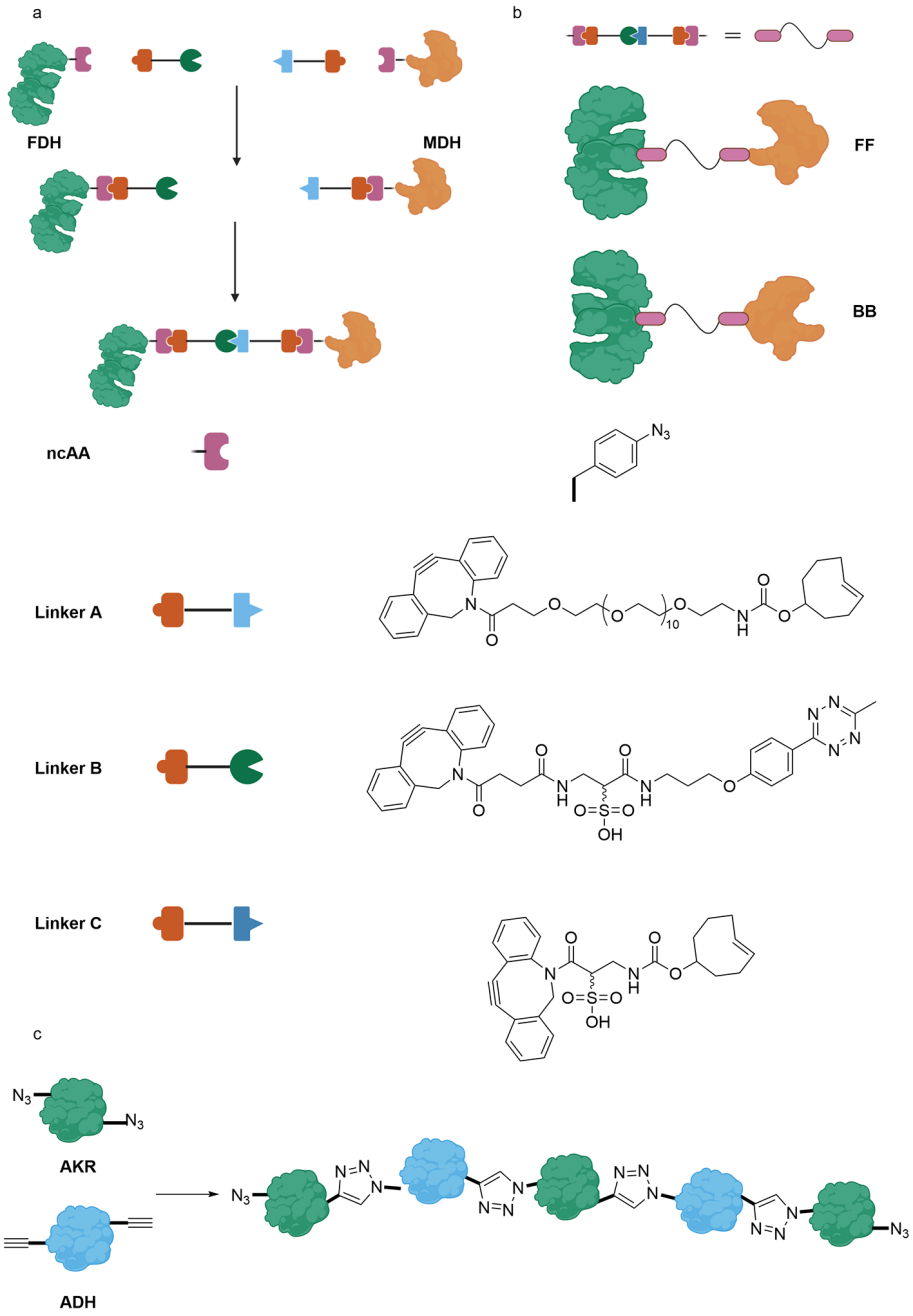
Different strategies for protein–protein coupling using ncAAs. (a) Conjugation of FDH and MDH using linkers A and B without a specific orientation. (b) Conjugation of FDH and MDH using linkers B and C generating a specific orientation. (c) Direct conjugation without linkers of AKR and ADH (ordered dual CLEs O-DCLEs approach). Part of the figure was created with BioRender.com. AKR: aldehyde ketone reductase, ADH: aldehyde ketone reductase, FDH: formate dehydrogenase, MDH: mannitol dehydrogenase, FF: Face to Face conformation, BB: Back to Back conformation.

The previously described studies involved combining two proteins without linkers. Meanwhile, Lim and co-workers^203^ developed a strategy using ncAAs and chemical linkers for site-specific coupling of formate dehydrogenase (FDH) and mannitol dehydrogenase (MDH) as depicted in Figure 7a. FDH catalyzes the oxidation of formate to produce NADH, which MDH can utilize to reduce d-fructose to d-mannitol. Initially, ***p*AzF** was incorporated at V237 in FDH and V417 in MDH. Then, each enzyme was reacted with a heterobifunctional linker through its alkyne moiety using SPAAC. Finally, the respective two enzyme-linker bioconjugates were coupled to each other through an inverse electron-demand Diels–Alder reaction. The multienzyme complex possessed a molar ratio of FDH to MDH of 2:1, with MDH linked to an FDH dimer. The catalytic activity was tested under low enzyme concentration, no stirring, and with excess substrates. Under these conditions, the *D*-mannitol generation rate depends on NADH diffusion between the enzymes. After 6 hours of reaction, the FDH-MDH conjugate produced double the desired product compared to the equivalent mixture of free enzymes. The improved yields are most likely related to the easier transport of NADH between the enzymes due to spatial closeness.^203^

Based on the initial promising results, the study of the assembly of FDH and MDH was continued by Lim and co-workers to underline the effect of the relative orientation of one enzyme to the other.^204^ Two different multienzyme complexes were developed, the first having a face-to-face orientation of the active sites (FF) and the second a back-to-back orientation (BB), as displayed in Figure 7b. For the FF orientation, sites close to the NAD^+^/NADH binding site were chosen for incorporation (V237 in FDH and V417 in MDH), and for the BB orientation sites, W172 for FDH and V271 in MDH. The catalytic efficiency was tested under conditions that allow the transfer of NADH to become the rate-limiting step at different multienzyme complex concentrations (20–80 nM). A more efficient NADH transfer was observed for FF compared to BB at 20 and 40 nM, with the difference being more significant at the lower concentration. Interestingly, at 80 nM, the results were somewhat similar. Overall, FF could produce ∼1.6-fold more *D*-mannitol compared to the BB assembly. Both conformations (FF and BB) showed improved efficiency compared to a mixture of free enzymes. The authors discussed that the FF’s improved efficiency is most likely due to the efficient substrate channeling. Moreover, BB is still more efficient than free enzymes, by keeping both enzymes close.^204^

Another approach using a linker for the assembly of two enzymes was proposed by Wang’s group to form dual enzyme CLEs (DCLEs). For this, ***p*AzF** was incorporated in AKR, ADH, or GDH. The different enzyme combinations to form DCLEs were cross-linked using a cyclooctyne-diyne linker through SPAAC. As a control, traditional CLEAs of AKR and GDH were prepared using glutaraldehyde. The AKR_ADH_DCLEs, AKR_GDH_DCLEs, and AKR_GDH_CLEA_1 were used for the synthesis of (*S*)-1-(2,6-dichloro-3-fluorophenyl) ethanol with NADPH regeneration using isopropanol. It was observed that the desired product was obtained with a higher yield and *ee* for both DCLEs complexes than for the CLEA, with AKR_ADH_DCLEs being the best catalyst. Indeed, AKR_ADH_DCLEs exhibited 76% yield, twelve times higher than that for AKR_ADH_CLEAs_1, and an *ee* of 99% versus 72%, respectively. Furthermore, AKR_ADH_DCLEs could be recycled for nine cycles, although its yield was reduced in the process (77% of its initial yield in the last cycle).^205^

In a follow-up study, the Wang group focused on better spatial control and organization of the enzymes and its effect on catalysis.^194^ For this, different enzyme cross-linked complexes were developed. First, ordered dual CLEs (O-DCLEs) of AKR and ADH were designed by direct cross-linking of AKR containing ***p*AzF** and ADH incorporating ***p*PaF** in each enzyme at two positions (Figure 7c). Second, less precise cross-linking CLEs (S-DCLEs) were prepared, in which just ***p*AzF** was incorporated in ADH and AKR, and the enzymes were assembled using a diyne linker. The apparent kinetic analysis was carried out using dihydro-4,4-dimethyl-2,3-furandione as a surrogate substrate. The catalytic efficiency of the O-DCLEs was ∼4 times higher than that of the corresponding S-DCLEs. Furthermore, the assemblies were tested for the asymmetric synthesis of (*R*)-1-(2-chlorophenyl)ethanol. A yield of 93% was obtained for O-DCLEs compared to 55% for S-DCLEs. The observed improved catalysis may be related to the assembly of the enzymes. In S-DCLEs, there is no selective cross-linking, so structures in which AKR or ADH aggregates with itself could be found, which would not contribute to the NADPH transfer among the enzymes. Meanwhile, for O-DCLEs, more precise control is expected, to avoid self-aggregates due to direct linking. The assemblies generated as depicted in Figure 7c could promote a more efficient substrate channeling.^194^

#### 4.2.2 Three Enzymes

**Figure 8:**
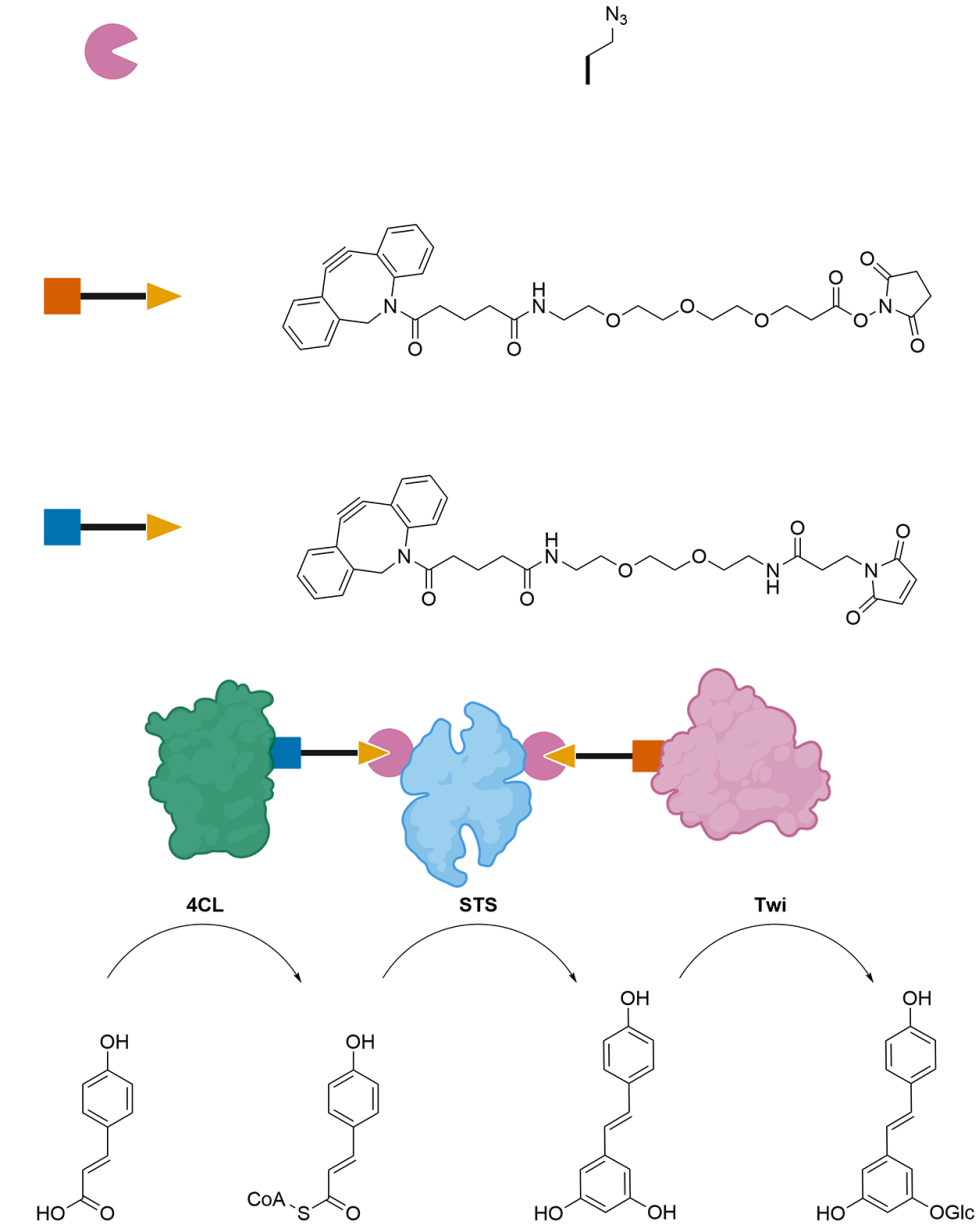
Multienzyme assembly using ncAAs and linkers for the cascade reaction to synthesize glycosylated resveratrol. Part of the figure was created with BioRender.com. 4CL: 4-coumarate:coenzyme A ligase, STS: stilbene synthase, and Twi: UDP-glucosyltransferase. Reproduced with permission from ref (195). Copyright 2013 American Chemical Society.

Assembly of more than two enzymes and their use beyond cofactor recycling cascades would be desirable. In this sense, Schoffelen and co-workers^195^ reported the development of a multienzymatic complex capable of performing a cascade reaction by assembling three different enzymes, partly using ncAAs. The multienzyme complex comprised 4-coumarate:coenzyme A ligase (4CL), stilbene synthase as a dimer (STS), and UDP-glucosyltransferase (Twi). The general approach is depicted in Figure 8. STS’s methionine sites were replaced by **Aha** at eleven positions, 4CL underwent a cysteine-maleimide coupling to a linker, and Twi underwent an *N*-hydroxysuccinimide coupling with a second type of linker. The assembly was completed upon SPAAC between the alkyne moiety of the linkers in 4CL/Twi and the azide moiety of **Aha** in STS. The bioconjugate was tested to obtain glycosylated resveratrol from *p*-coumaric acid (Figure 8). Overall, the multienzymatic complex had a similar kinetic behavior to the equivalent mixture of free enzymes. Besides the desired multienzymatic complex, some side products bearing just two enzymes, such as 4CL-STS-4CL, were found, highlighting the need to use different click reactions for each link.

### 4.3 Discussion and Perspectives on the Use of ncAAs for Enzyme Assemblies

The examples reviewed show that ncAAs are an intriguing alternative for developing more precise (multi)enzyme assembly complexes compared to traditional methods. Using ncAAs facilitates a more rational design of the assemblies, allowing control regarding the exact locations and number of sites for linking. This approach minimizes possible disturbance of the active site of the enzymes with high spatial control regarding the particular orientation of enzymes within the assembly. While using ncAAs to develop assemblies for biocatalysis is still understudied, it holds great potential for future research. Indeed, different examples in this review portray that CLEs are a worthy alternative to conventional CLEAs, showing in some examples better catalytic activity. Furthermore, CLEs can be produced using cell lysate due to the bio-orthogonality of the handles. Besides, different studies showed that multienzyme complexes prepared with ncAAs can be used for cofactor recycling and cascade reactions that, with careful preparation, benefit from substrate channeling effects, among others.

However, several challenges must be addressed to exploit ncAAs’ potential fully. For instance, the success of the bioconjugations and subsequent catalytic activity is heavily influenced by parameters such as the choice of ncAAs (even bearing the same orthogonal moiety), the site(s) of incorporation, and the number of positions for incorporation. Here, computational design, along with the screening of a larger set of variants, could prove beneficial. Furthermore, some studies have reported the formation of undesired assemblies of proteins, lacking the required composition of the final assembly. This can be overcome by direct coupling between the enzymes through the incorporated ncAAs. However, this is challenging for assemblies of more than two enzymes. Therefore, further research to design ncAAs bearing new chemical moieties that allow click chemistry reactions orthogonal to SPAAC, for example, inverse electron-demand Diels–Alder reactions, would significantly advance the field.

## 5 Artificial Enzymes Featuring Noncanonical Amino Acids

The promiscuous activity of many natural enzymes can be exploited to create biocatalysts for new-to-nature transformations.^12,206^ In parallel, computational redesign and generation of *de novo* protein constructs aid in the creation of tailored rudimentary enzymes that can serve as starting points for further evolution.^8,207^ Incorporation of ncAAs into protein hosts through SCS is an alternative strategy which allows for the creation of novel active sites featuring catalytic functionalities not available in nature.^47^ These rudimentary artificial enzymes generally display low catalytic efficiency at first that can be improved upon by directed evolution. This section summarizes the progress on the design of artificial enzymes for organo-, metallo- and photoredox-catalysis in which ncAAs are utilized to introduce new catalytic activity previously unknown for the protein scaffold. This includes examples in which the ncAA is used as a bio-orthogonal handle to introduce new-to-nature catalytic functionalities, as well as examples in which the ncAA itself harbors organocatalytic, metal-binding, or photosensitizing properties.

### 5.1 Enzymes with Organocatalytic ncAAs

**Figure 9:**
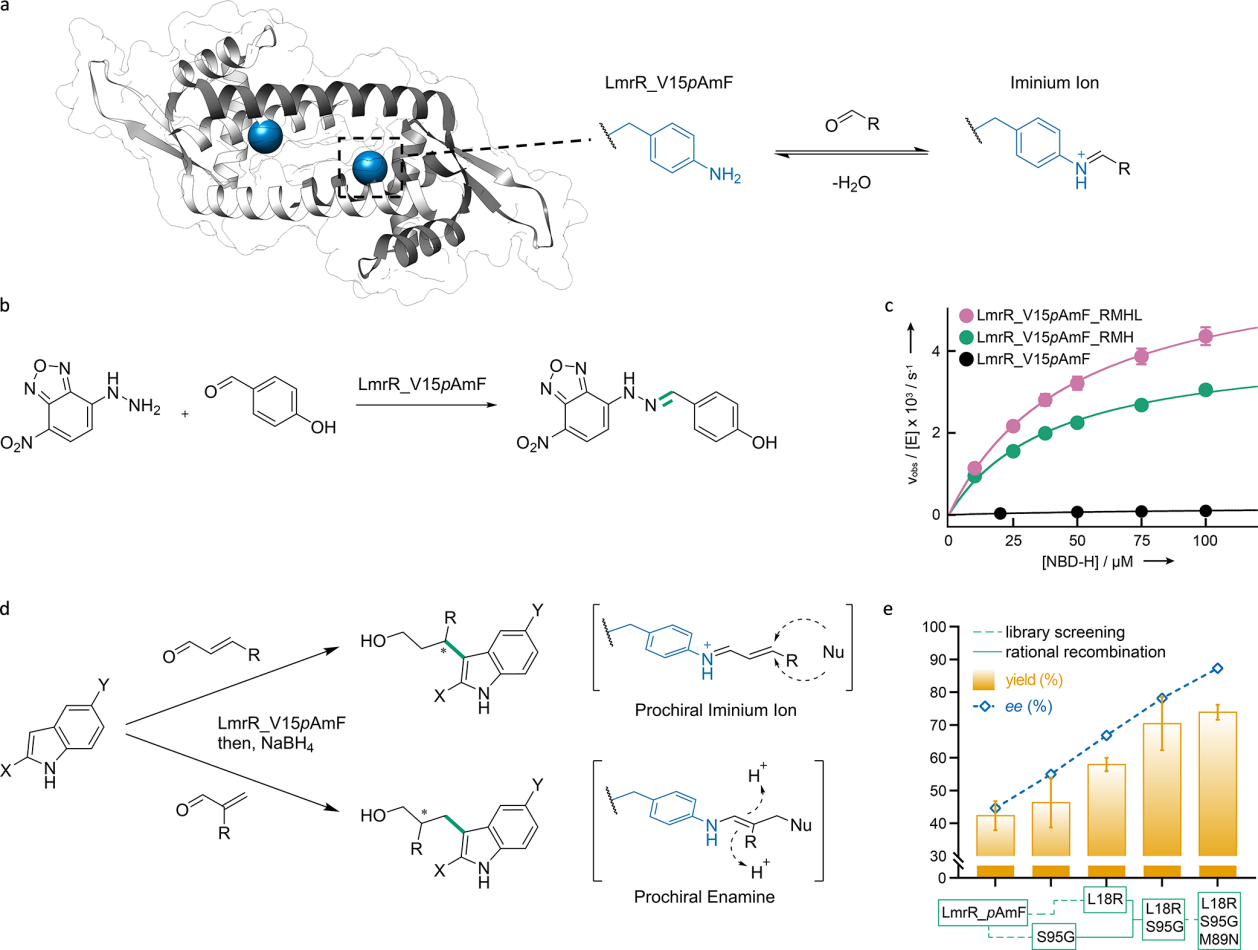
(a) Design of the artificial enzyme LmrR_V15***p*AmF** featuring ***p*AmF** as catalytic residue. The location of the catalytic residues in the dimeric LmrR protein is depicted as blue spheres (PDB 3F8B). (b) Chromogenic hydrazone formation reaction between NBD-H and 4-HBA. (c) Comparison of saturation kinetics of the hydrazone formation at a 4-HBA concentration of 5 mM for LmrR_V15***p*AmF** and the best variants obtained after directed evolution. Reproduced with permission from ref (218). Copyright 2019 Wiley. (d) Friedel–Crafts alkylation of indoles with β-substituted-α,β-unsaturated aldehydes *via* a prochiral iminium-ion intermediate (top). Friedel–Crafts alkylation/enantioselective protonation using α-substituted-α,β-unsaturated aldehydes *via* protonation of a prochiral enamine intermediate (bottom). (e) Evolutionary trajectory of LmrR_V15***p*AmF**_RGN for the Friedel–Crafts alkylation between 2-methylindole and hexenal. Reproduced with permission from ref (221). Copyright 2021 American Chemical Society.

Organocatalysis, acknowledged with the Nobel Prize in chemistry in 2021, comprises a set of powerful methodologies spanning a wide range of synthetically relevant asymmetric transformations.^208^ Although highly versatile, organocatalysts in organic synthesis often display modest turnover numbers and commonly require organic solvents and relatively high catalyst loading.^209^ In recent years, efforts have been made toward translating organocatalysis into aqueous environments and enzymes.^210^ Toward this goal, SCS has been applied for the incorporation of ncAAs with inherent organocatalytic properties into protein scaffolds, leading to the creation of artificial enzymes featuring new-to-nature organocatalytic machinery. ***p*AmF** is a ncAA that features a uniquely reactive aniline moiety in its side chain, which can be introduced using SCS methodologies.^211^ The Roelfes lab has exploited this unique reactivity to create a variety of artificial enzymes featuring ***p*AmF** as organocatalytic residue for iminium-type catalysis (Figure 9a).^212^ Their protein scaffold of choice is the *Lactococcal* multidrug resistance regulator (LmrR), a transcriptional regulator belonging to the PadR family.^213^ It is a relatively small homodimeric protein harboring a hydrophobic pocket exhibiting promiscuous binding capabilities stemming from its natural function in multidrug resistance regulation.^214^ While LmrR has no native catalytic function, it has been found to be a privileged scaffold for the design of artificial enzymes.^24^

The first LmrR-based artificial enzyme featuring an organocatalytic residue was created by incorporation of ***p*AmF** into an LmrR variant with reduced DNA binding capacity^215^ at four different positions lining the promiscuous binding pocket (V15, N19, M89 and F93).^216^ Due to low efficiency of direct incorporation of ***p*AmF**, an engineered *Mj*Tyr OTS^217^ was used to introduce ***p*AzF**, of which the azido group was subsequently reduced post-translationally using a Staudinger reaction with tris(2-carboxyethyl) phosphine. The catalytic properties of the new artificial enzymes were evaluated in a model hydrazone formation reaction between 4-hydrazino-7-nitro-2,1,3-benzoxadiazole (NBD-H) and 4-hydroxybenzaldehyde (4-HBA) (Figure 9b). LmrR_WT exhibited background reactivity, likely due to the increased effective molarity provided by the promiscuous binding pocket, but introduction of ***p*AmF** at position V15 significantly increased this activity. Further investigation, including trapping of the transiently formed Schiff base intermediate, confirmed the catalytic role of V15***p*AmF** and indicated the importance of properly positioning the catalytic residue in the pocket with respect to two central tryptophans (W96 and W96′) involved in the binding of substrates. Subsequent directed evolution using a chromogenic assay based on the hydrazone formation reaction yielded LmrR_V15***p*AmF**_RMHL containing four additional mutations (A92R_N19M_F93H_A11L) showing a 74-fold improvement in apparent catalytic efficiency (1.85 M^–1^ s^–1^ and 137 M^–1^ s^–1^, respectively) (Figure 9c).^218^

In further work, Ofori Atta and colleagues^219^ demonstrated that this artificial enzyme could be applied in *in vivo* biocatalytic cascades in *E. coli* that entail the biosynthesis of the aldehyde substrate using canonical enzymes, followed by hydrazone formation by the artificial enzyme. As the reduction of ***p*AzF** was not feasible *in vivo*, expression conditions were first optimized to improve the efficiency of direct incorporation of ***p*AmF** using the dedicated *Mj*Tyr OTS for this ncAA.^211^*In vivo* biosynthesis of benzaldehyde from exogenously supplied benzyl alcohol was achieved using whole *E. coli* cells expressing 5-hydroxymethylfurfural oxidase (HMFO) from *Methylovorus sp. strain MP688*.^220^ Combined with LmrR_V15***p*AmF**_RMH, a variant found in the previously described evolution, the *in vivo* biocatalytic cascade gave the corresponding hydrazone product with 81% yield after 2 h (vs 6% yield after 2 h using only HMFO).

Leveson-Gower and co-workers^221^ applied LmrR_V15***p*AmF** in a more challenging C-C bond forming Friedel–Crafts (FC) alkylation using β-substituted-α,β-unsaturated aldehydes as substrates. The iminium ion intermediate formed upon condensation with ***p*AmF** activates the β-position for nucleophilic attack by indole substrates, creating a stereogenic center in the process (Figure 9d). This resembles the application of MacMillan’s imidazolidinone organocatalysts^222^ in FC reactions, yet now placed in a chiral protein environment. LmrR_V15***p*AmF** was found to catalyze the FC alkylation between 2-methylindole and hexenal with 42% yield and 45% *ee*. Subsequent directed evolution targeting key residues in the LmrR pocket identified a triple mutant, LmrR_V15***p*AmF**_RGN (L18R_S95G_M89N), with increased activity and enantioselectivity, yielding the FC alkylation product with 74% yield and 87% *ee* (Figure 9e). Moreover, the evolved variant LmrR_V15***p*AmF**_RGN exhibited significantly lower activities for the hydrazone formation reaction, even lower than the activity of the parent LmrR_V15***p*AmF**. This indicated divergent evolutionary paths for the hydrazone formation and FC alkylation reaction. The versatility of this design was further demonstrated by the ability of LmrR_V15***p*AmF** to perform a challenging tandem Friedel–Crafts alkylation/enantioselective protonation (FC-EP) using α-substituted-α,β-unsaturated aldehydes.^223^ The stereogenic center in this transformation is not formed during the C-C bond formation, but by protonation of the prochiral enamine intermediate (Figure 9d). While enantioselective protonation in water is generally challenging,^224^ it was shown that LmrR_V15***p*AmF** could perform the FC-EP in 71% yield and 88% *ee* when using 2-methylindole and methacrolein as substrates. Good activities and enantioselectivities were also obtained for a variety of other α-substituted-α,β-unsaturated aldehydes and indoles.

**Figure 10:**
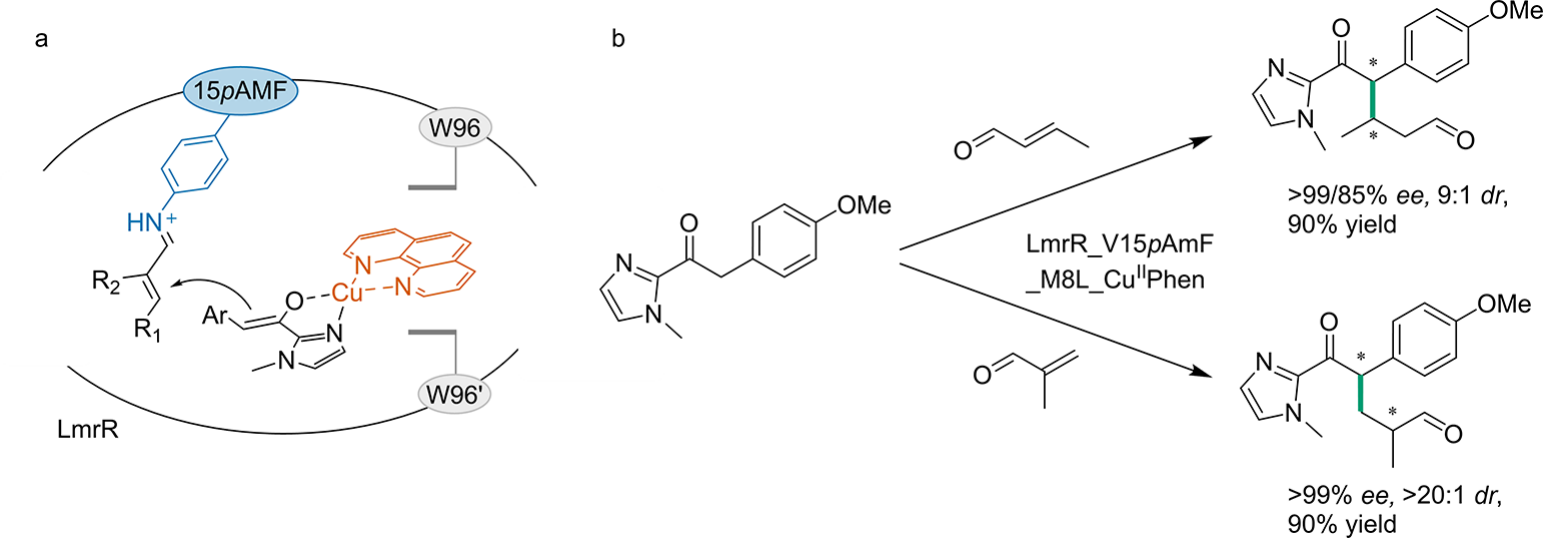
(a) Schematic representation of LmrR_V15***p*AmF** with Cu^II^phen bound between the two central tryptophans (W96 and W96′) for the synergistic catalysis of enantioselective Michael addition reactions. (b) LmrR_V15***p*AmF**_M8L_Cu^II^phen catalyzed Michael addition between 2-(4-methoxyphenyl)-1-(1-methyl-1H-imidazol-2-yl)ethan-1-one and crotonaldehyde (top), or Michael addition/enantioselective protonation with methacrolein (bottom).

Zhou and colleagues^225^ created an LmrR-based artificial enzyme containing two abiological catalytic moieties acting synergistically to catalyze enantioselective Michael addition reactions. Inspired by earlier work in which LmrR was employed as an artificial metalloenzyme (ArM),^226^ a design was made employing supramolecularly bound Cu(1,10-phenanthroline)(NO_3_)_2_ (Cu^II^phen) in between the two central tryptophans as a Lewis acid activator and genetically encoded ***p*AmF** as organocatalytic residue. Cu^II^phen allowed activation of nonreadily enolizable ketone substrates in a reaction with α,β-unsaturated aldehydes, which in turn are activated for conjugate addition through iminium ion formation with ***p*AmF** (Figure 10a). LmrR_V15***p*AmF**_Cu^II^phen showed good initial activities and enantioselectivities, giving the Michael addition product of the reaction between an acyl imidazole and crotonaldehyde in 72% yield, 8:1 *dr*, and 99/85% *ee*. With only one further mutation, the variant LmrR_V15***p*AmF**_ M8L_Cu^II^phen was created, which displayed both improved activity and enantioselectivity compared to the parent (90% yield, 9:1 *dr* and > 99/85% *ee*) (Figure 10b).

In a follow-up study, LmrR_V15***p*AmF**_M8L_Cu^II^phen was employed for the tandem Michael addition/enantioselective protonation (MA-EP).^227^ Similar to the FC-EP described previously, following conjugate addition, the chiral product is obtained by controlled delivery of a proton to the prochiral enamine intermediate. Using methacrolein as the Michael acceptor, the MA-EP product was obtained with 90% yield and excellent >20:1 *dr* and >99% *ee* (Figure 10b). Moreover, excellent stereoselectivities were also observed for a panel of different Michael acceptors and donors. These studies illustrated that synergistic combination of two abiological catalytic moieties poses an attractive way forward to create enzymes for new-to-nature transformations.

**Figure 11:**
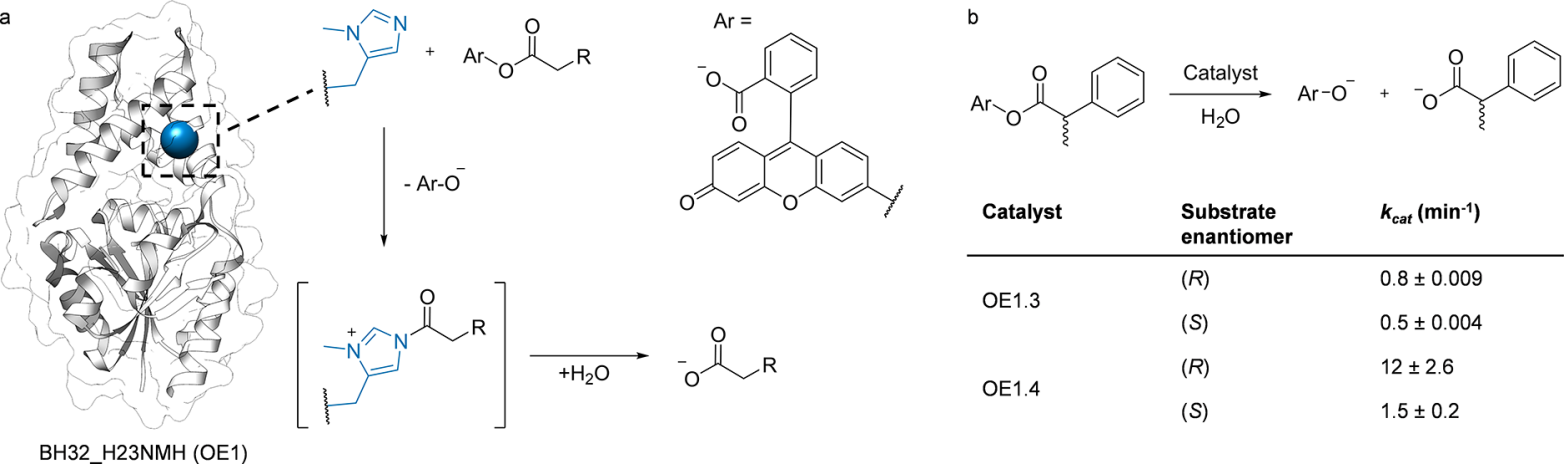
(a) Design of artificial enzyme OE1 (BH32_H23**NMH**) featuring **NMH** as organocatalytic nucleophile in the hydrolysis of fluorescein 2-phenylacetate. Acylation of the **NMH** nucleophile leads to the generation of a reactive acyl-imidazolium intermediate. The location of the catalytic residue in the BH32 protein is depicted as a blue sphere (PDB 6Q7Q). (b) *k*_cat_ of OE1.3 and OE1.4 in the hydrolysis of fluorescein (*R*)-2-phenylpropanoate and fluorescein (*S*)-2-phenylpropanoate.

Burke and co-workers^228^ created an artificial esterase by incorporation of **NMH** as a noncanonical organocatalytic nucleophile into the computationally designed protein scaffold BH32 (Figure 11a).^229^**NMH** is a histidine analog methylated at N_δ_, thus resembling the nucleophilic behavior of dimethylaminopyridine.^230^ Using a theozyme approach, BH32 was originally designed as a *de novo* enzyme for the Morita–Bayliss–Hillman (MBH) reaction harboring a histidine (H23) as catalytic nucleophile.^229^ The BH32 enzyme was also found to exhibit activity toward the hydrolysis of fluorescein 2-phenylacetate, yet at low turnover due to the formation of a His23-acyl intermediate resistant to hydrolysis. An engineered *Mb*Pyl OTS^231^ was used to replace His23 with **NMH** as noncanonical nucleophile, resulting in the creation of organocatalytic esterase (OE)1 with significantly increased hydrolytic activity. The increased activity was attributed to the formation of a more reactive acyl-imidazolium intermediate formed when using **NMH**, facilitating subsequent hydrolysis (Figure 11a). OE1 was then subjected to a directed evolution campaign leading to variant OE1.3, which contained six mutations (L10P_A19H_S22M_E46N_P63G_C125G) and showed a 15-fold increase in catalytic efficiency compared to the parent. Improved esterase activities were also observed for a panel of different fluorescein esters. Moreover, OE1.3 could serve as template for further evolution of a more enantioselective esterase for the hydrolysis of chiral fluorescein 2-phenylpropanoate. This resulted in identification of variant OE1.4 with an additional three mutations (N14Q_S124L_D180F) that showed an 8-fold higher *k*_*cat*_ toward the (*R*)-enantiomer (Figure 11b).

More recently, **NMH** in BH32 was also reported as an organocatalytic nucleophile in a Morita–Baylis–Hillman reaction.^232^ Evolution of this MBHase featuring **NMH** resulted in a significantly altered evolutionary pathway compared to canonical histidine as the catalytic nucleophile in the previously evolved BH32.14.^229^ Moreover, the catalytic activity of the evolved **NMH**-based artificial MBHase surpassed that of BH32.14 by 13-fold.

**Figure 12:**
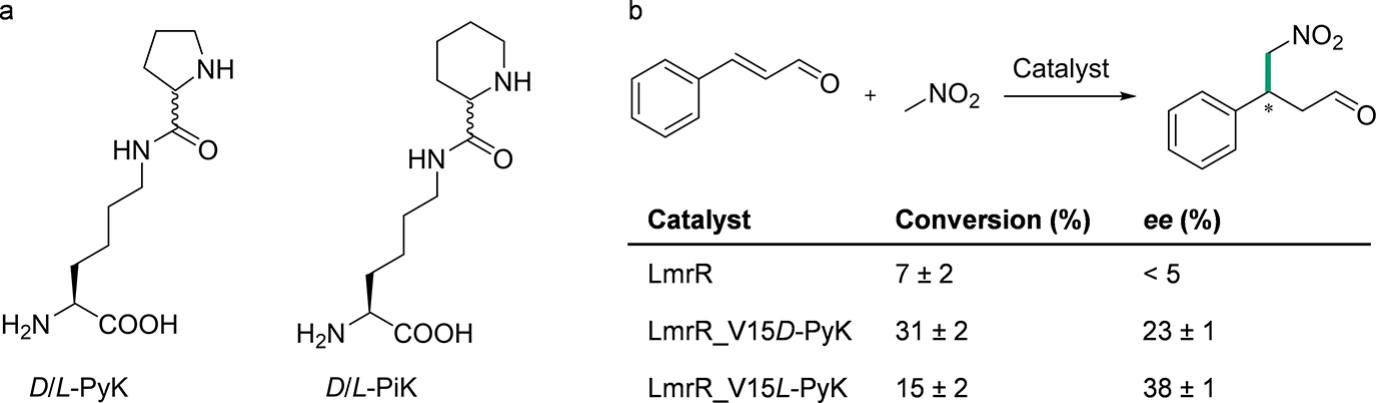
(a) Structures of pyrrolidine- and piperidine-based pyrrolysine mimicking ncAAs in both stereogenic forms connected at the ε-nitrogen of lysine (***D*/*L*-PyK**, ***D*/*L*-PiK**). (b) Michael addition reaction between cinnamaldehyde and nitromethane performed by different LmrR variants.

Inspired by established secondary amine organocatalysts such as *L*-proline and Hayashi–Jørgensen catalysts used in organic synthesis,^233^ Gran-Scheuch and co-workers^234^ set out to genetically incorporate a panel of noncanonical pyrrolysine mimics harboring secondary amines to expand the available toolbox for creating artificial enzymes with organocatalytic residues. Pyrrolidine- and piperidine-based ncAAs were synthesized in both stereoisomeric forms connected at the ε-nitrogen of lysine (*N*^ε^-((*D*)-pyrrolidine-2-carbonyl)-*L*-lysine (**82**, ***D*-PyK**), *N*^ε^-((*L*)-pyrrolidine-2-carbonyl)-*L*-lysine (**83**, ***L*-PyK**) and *N*^ε^-((*D*)-piperidine-2-carbonyl)-*L*-lysine (**84,*D*-PiK**), *N*^ε^-((*L*)-piperidine-2-carbonyl)-*L*-lysine (**85,*L*-PiK**), respectively) (Figure 12a). Upon screening of a library of pyrrolysyl aaRS/tRNA pairs, it was found that the WT *Mb*Pyl OTS could successfully incorporate all of the four secondary amine ncAAs into sfGFP. Next, the functional potential of these ncAAs was demonstrated by incorporation into LmrR at position V15 and subsequent evaluation in a model Michael addition reaction between cinnamaldehyde and nitromethane (Figure 12b). LmrR_V15***D*-PyK** and LmrR_V15***L*-PyK** gave 31% and 15% conversion and 23% and 38% *ee*, respectively. This was an increase in activity and enantioselectivity compared to LmrR_WT (7% conversion, <5% *ee*), indicating the involvement of the pyrrolidine harboring ncAAs as organocatalytic residues in this model iminium reaction. This was further confirmed by trapping of the transiently formed Schiff base intermediate. Despite modest catalytic performance of the newly created artificial enzymes, the authors expanded the available toolbox of organocatalytic residues with novel secondary amine harboring ncAAs.

More recently, Longwitz and colleagues^235^ reported boron catalysis in a designed artificial enzyme by employing genetically encoded ***p*BoF** as organocatalytic residue. LmrR with ***p*BoF** incorporated at position M89 yielded an artificial boronic acid dependent enzyme catalyzing the stereoselective condensation of α-hydroxyketones with hydroxylamine to form oximes in a kinetic resolution. Subsequent directed evolution led to an improved variant with significantly improved rate constant and E-values up to 146.

Overall, these studies show that incorporation of ncAAs into protein scaffolds allows the creation of rudimentary artificial enzymes featuring organocatalytic machinery not generally observed in nature. This allows for the translation of several well-known organocatalytic transformations into enzymes. In contrast to other artificial enzyme designs requiring a posttranslational modification or exogenously supplied cofactor, directed evolution of artificial enzymes featuring a ncAA as organocatalytic residue is facilitated due to the fact that the catalytic moiety can be fully genetically encoded. Fully genetically encodable organocatalytic residues also facilitate the use of ArEs in whole-cell and *in vivo* catalysis, as demonstrated by the use of an ArE featuring directly incorporated ***p*AmF** in an *in vivo* biocatalytic cascade. However, most examples of ArEs employing ncAAs as organocatalytic residues so far are more proof-of-principle, and the scope of ncAAs used as organocatalytic residues is limited to a few examples, with a narrow chemical diversity. Nonetheless, it holds promise for translation of more challenging reactions into enzymes, as exemplified by the more recently demonstrated ArE featuring ***p*BoF**, performing boron catalysis. Moreover, the discovery and evolution of new OTSs can aid in expanding the scope of ncAAs that can be used as organocatalytic residues.

### 5.2 Artificial Metalloenzymes

Nature employs cofactors and metal ions to expand its functional scope and create metalloenzymes with diverse reactivities. While the promiscuity of these enzymes can be exploited to evolve new functions, the possibilities are limited by the defined set of naturally prevalent metals and cofactors.^12^ Introduction of non-native metal-binding sites and transition metals used in organic chemistry into proteins allows for the expansion of the chemical repertoire of enzymes, creating so-called artificial metalloenzymes.^236^ Strategies toward this goal include the exchange of native cofactors with non-natural alternatives, and site-specific conjugation of catalytically active transition metal complexes into proteins. The introduced non-natural component presents the first coordination sphere, while the protein provides a second (chiral) coordination sphere that can be genetically optimized. To this end, ncAAs have also been employed to facilitate the creation of ArMs.^25^ This section summarizes the progress in which ncAAs have been utilized to incorporate non-natural metal cofactors or metal-binding sites into protein scaffolds to afford ArMs with novel catalytic sites. A distinction is made between ArMs in which the ncAA is used as bio-orthogonal handle to introduce non-natural metal cofactors (section 5.2.1) and ArMs in which the ncAA itself is utilized as metal-binding ligand (section 5.2.2). Finally, examples in which ncAAs have been utilized to engineer and develop metallocatalytic activities in myoglobin, a heme-binding protein natively involved in oxygen storage, will be discussed (section 5.2.3).

#### 5.2.1 ncAA as Bio-orthogonal Handle

**Figure 13:**
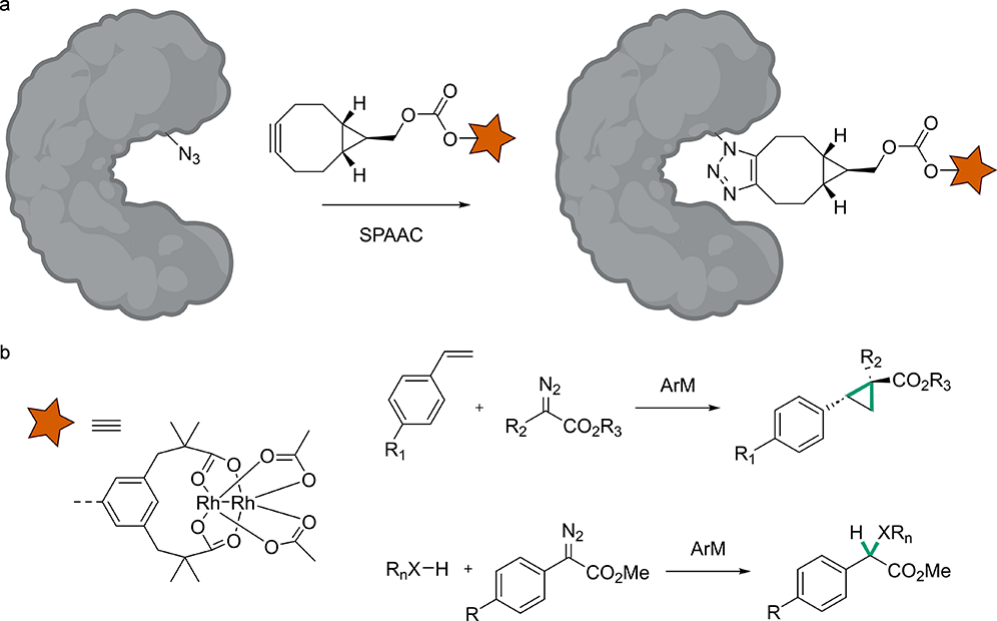
(a) Design of artificial enzymes *via* genetic incorporation of ***p*AzF** as bio-orthogonal handle into a protein scaffold, followed by SPAAC to introduce a BCN-substituted catalytic moiety (represented as orange star). (b) Dirhodium complex covalently anchored *via* SPAAC to create ArMs catalyzing intermolecular cyclopropanation and carbene insertion reactions. Part of the figure was created with BioRender.com

Yang et al.^237^ demonstrated the application of ncAAs in the creation of ArMs by using ***p*AzF** as bio-orthogonal handle to incorporate non-natural metal cofactors into protein scaffolds through SPAAC (Figure 13a). Using SCS, ***p*AzF**(217) was incorporated at distinct positions in the thermostable α,β-barrel protein tHisF^238^ and subsequently covalently linked to a bicyclo[6,1,0]nonyne (BCN)-substituted dirhodium complex (Figure 13b). The resulting constructs exhibited activity toward dirhodium-catalyzed intermolecular cyclopropanation and Si-H insertion reactions, albeit without enantioselectivity and in lower yields than compared to the cofactor alone. Nonetheless, this study demonstrated a general approach for the creation of new ArMs.

In subsequent work,^239^ this method was applied to incorporate the dirhodium complex into a POP from *Pyrococcus furiosus*.^240^ While POP is natively a serine protease, in this case, the catalytic S477 was replaced with ***p*AzF** through SCS. Furthermore, four alanine mutations were introduced to increase the active site access, resulting in a variant called POP-ZA_4_. Upon SPAAC conjugation of the dirhodium complex, the designed ArM exhibited basal catalytic activity and enantioselectivity toward the cyclopropanation of styrene (Figure 13b). In contrast to tHisF, this indicated that the second coordination sphere provided by the POP scaffold could impart enantioselectivity to the dirhodium cofactor. Further optimization was performed by rationally mutating targeted residues in the active site near the dirhodium complex. This gave rise to three additional mutations (H328_F99_F594), yielding POP-ZA_4_-HFF that exhibited significantly improved activity and enantioselectivity (up to 92% *ee*) for a variety of styrenes and donor–acceptor carbene precursors. Translating water-sensitive metallocatalysis such as carbene transfer reactions into aqueous conditions can be challenging due to unwanted deactivation pathways.^241^ Encapsulating the metal catalyst into a protein environment can aid in protection of the reactive carbene intermediates. This was also demonstrated in the case of POP-ZA_4_, which after genetic optimization displayed a reduction in reaction of dirhodium–carbene intermediates with water.

In another study, instead of targeted mutagenesis, random mutagenesis was performed to evolve the basal cyclopropanation activity of the dirhodium conjugated POP-ZA_4_ previously described.^242^ Using an optimized high-throughput directed evolution approach, a total of twelve mutations spanning both active site and distal residues were found, affording variant POP-ZA_4_-3-VRVH with enhanced activity and selectivity. Notably, the evolved ArM surpassed the activity of the previously rationally designed POP-ZA_4_-HFF, demonstrating the importance of distal mutations. Next to that, the obtained ArM also exhibited activity toward other dirhodium-catalyzed reactions, including Si–H, N–H and S–H insertion (Figure 13b). Furthermore, conjugation of the dirhodium complex at alternate position F413***p*AzF**, followed by directed evolution, led to the creation of an ArM with opposite enantioselectivities than obtained with POP-ZA4-3-VRVH. This underlined the versatility of this approach for creating artificial metalloenzymes with synthetically relevant properties.

#### 5.2.2 ncAA as Metal-Binding Ligand

In contrast to the indirect incorporation of metallocatalytic moieties *via* click chemistry, it is also possible to directly incorporate ncAAs with inherent metal-binding properties *via* SCS. In this light, Xie et al.^243^ engineered a *Mj*Tyr OTS for the genetic incorporation of **BpyA**. The bipyridyl moiety can strongly chelate a variety of transition metal ions such as Cu^II^ and Fe^II^, and incorporation of **BpyA** has led to the creation of a variety of metal-binding proteins.^188,244,245^

**Figure 14:**
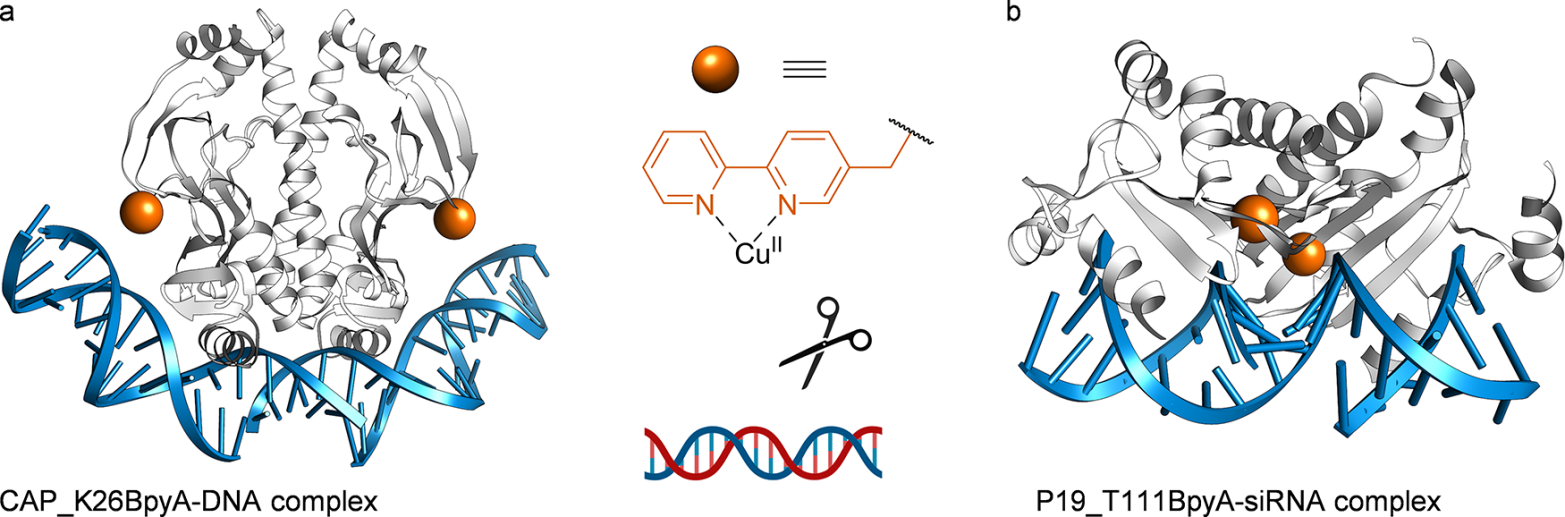
Artificial endonucleases featuring copper-bound **BpyA**. (a) Incorporated at position K26 of CAP for cleavage of DNA (PDB 1J59). (b) Incorporated at position T111 of p19 for cleavage of RNA (PDB 1RPU). Positions of **BpyA** incorporation are depicted as orange spheres, and DNA or RNA molecules are depicted in blue.

Lee and Schultz were among the first to exploit the metal-binding properties of **BpyA** to create an ArM with endonuclease activity.^246^ Previous studies showed that attachment of divalent metal complexes to a DNA binding agent can lead to oxidative cleavage of DNA *via* generation of ROS mediated by the metal complex.^247^ Inspired by these results, Lee and Schultz incorporated **BpyA** into the DNA binding region of catabolite activator protein (CAP).^248^ Upon complexation of **BpyA** with Cu^II^ and in the presence of a reducing agent, the construct could specifically cleave the DNA-fragment bound by CAP (Figure 14a), demonstrating the first example of an ArM employing a genetically incorporated ncAA as metallocatalytic residue.

A similar approach was applied by Ahmed and co-workers,^249^ who used **BpyA** for the creation of an endonuclease able to degrade noncoding RNAs (Figure 14b). The ArM design was based on the Tombusvirus p19 protein, an RNA-binding protein with high selectivity toward small double-stranded RNAs.^250^**BpyA** was incorporated at positions K67 and T111, near the RNA binding pocket of p19. Upon chelation with Cu^II^, the constructs were evaluated for nuclease activity using a model short interfering RNA (siRNA). While p19_WT and p19-K67**BpyA** were not active, p19-T111**BpyA** was able to specifically cleave the siRNA. The utility of p19-T111**BpyA** as artificial endonuclease was subsequently further demonstrated by cleavage of human microRNA miR-122, a critical host factor for the hepatitis C virus. This illustrated the potential of such artificial endonucleases featuring **BpyA** as therapeutic tool targeting microRNAs involved in disease progression.

**Figure 15:**
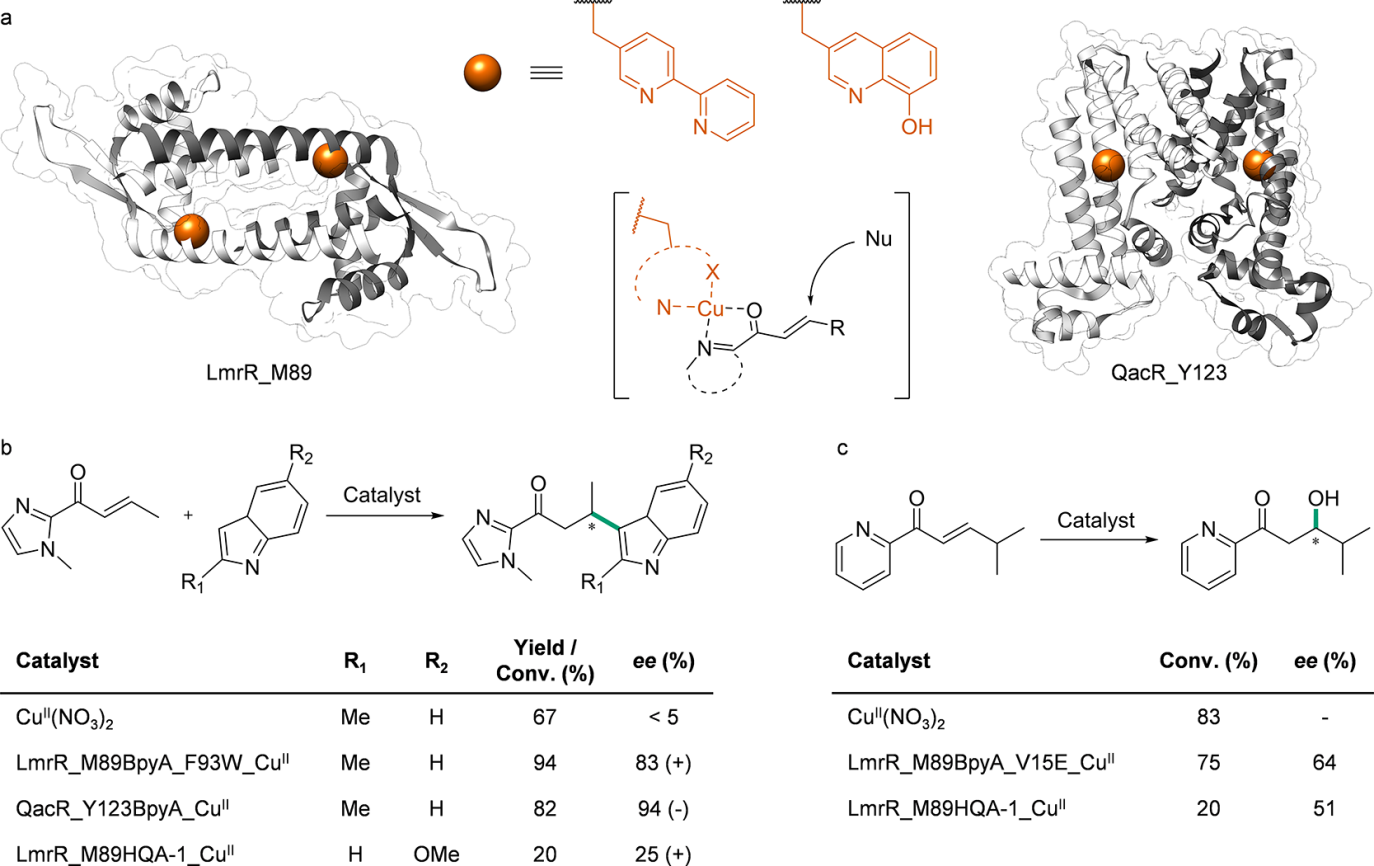
(a) Design of ArMs created by incorporation of metal-binding ncAAs **BpyA** or **HQA-1** at position M89 in LmrR (PDB 3F8B) or position Y123 in QacR (PDB 1JTY). Locations of **BpyA** incorporation are depicted as orange spheres. The formed ncAA-Cu^II^ complex serves as Lewis acid for activation of α-β-unsaturated acyl-imidazole or -pyridine substrates for nucleophilic attack. (b) Summary of enantioselective Friedel–Crafts reactions catalyzed by LmrR and QacR variants featuring different metal-binding ncAAs. (c) Enantioselective enone hydration catalyzed by LmrR variants featuring different metal-binding ncAAs.

The Roelfes group has previously reported the construction of a variety of ArMs *via* cysteine conjugation of Cu^II^-complexes into the LmrR protein scaffold.^215,251^ In subsequent work, genetically incorporated **BpyA** was used as new copper binding site to create ArMs that catalyze enantioselective FC alkylations.^252^**BpyA** was incorporated at three different positions (N19, M89 and F93) and upon chelation with copper, the **BpyA**-Cu^II^ complex could serve as Lewis acid catalyst in the FC reaction by activating an α-β-unsaturated acyl-imidazole substrate for nucleophilic attack of an indole derivative (Figure 15a). In contrast to using LmrR_WT in combination with free Cu^II^(NO_3_)_2_, enantioselectivity was observed when the newly created ArMs were used. Interestingly, the ArM with **BpyA** incorporated at F93 exhibited opposite enantioselectivity than the ArMs with **BpyA** at N19 and M89. The variant with the highest initial selectivity, LmrR_M89**BpyA**_Cu^II^, was subsequently subjected to a mutagenesis study targeting residues in proximity of the ncAA. Two individual mutations, H86A and F93W, were found to improve the conversion and selectivity of LmrR_M89**BpyA**_Cu^II^ toward different substrate derivatives, reaching up to 83% *ee* (Figure 15b).

LmrR_M89**BpyA** was later applied in the enantioselective hydration of enones.^253^ Enantioselective hydration reactions require the challenging activation of water as nucleophile and its controlled addition to one pro-chiral face of the substrate. In this light, Drienovská and co-workers performed a multiscale computational study to redesign LmrR_M89**BpyA** for enone hydration of α,β-unsaturated 2-acyl pyridine (Figure 15c). This resulted in variant LmrR_M89**BpyA**_V15E, in which the introduced glutamic acid was proposed to act as general base, involved in the activation of water and positioning it with respect to one of the pro-chiral faces of the **BpyA**-Cu^II^-enone complex. LmrR_M89**BpyA**_V15E_Cu^II^ indeed exhibited higher enantioselectivities (64% *ee vs* 42% *ee* for LmrR_M89**BpyA**_Cu^II^) and gave rise to a 3-fold improvement in catalytic efficiency.

In another study, it was found that LmrR_M89**BpyA** and mutants thereof could also chelate other first row transition metals and subsequently bind and stabilize a radical semiquinone. Although no catalysis was reported, these findings could facilitate potential future efforts toward harnessing the chemistry of unstable radicals in aqueous media.^254^ Overall, these studies showed that careful design of the second coordination sphere provided by the LmrR scaffold can lead to the optimization of these basal ArMs toward different transformations with varying substrates.

Bersellini and colleagues^255^ created a series of ArMs by incorporating **BpyA** into three multidrug resistance regulator proteins, QacR, CgmR and RamR, belonging to the TetR family.^256^ Similar to LmrR, these proteins contain hydrophobic pockets with promiscuous binding capabilities. For each TetR protein, **BpyA** was incorporated at four different positions lining the hydrophobic pocket. Following **BpyA**-Cu^II^ complex formation, the constructs were evaluated in the FC alkylation reaction previously described. All TetR-based ArMs displayed activity with different enantiopreferences depending on the **BpyA** incorporation site. Interestingly, control experiments using free Cu^II^(NO_3_)_2_ in combination with the WT TetR proteins also gave rise to enantioselective catalysis, suggesting the possibility of copper binding by the scaffolds alone. The best ArM, QacR_Y123**BpyA**_Cu^II^, reached up to 94% *ee* with high conversion (Figure 15b). Moreover, this variant yielded the opposite enantiomer as obtained previously with LmrR_M89**BpyA**_Cu^II^.^252^ This demonstrated that by incorporation of **BpyA** into different protein scaffolds, enantiocomplementary ArMs can be created.

Klemencic and colleagues^257^ recently reported the creation of new copper ArMs by introduction of **BpyA** into the human steroid carrier protein *via* either genetic incorporation or cysteine conjugation. This led to the creation of two different ArMs able to catalyze the previously described enantioselective FC alkylation (Figure 15b). Interestingly, despite using the same protein scaffold, the two designs exhibited opposite enantioselectivities.

Next to **BpyA**, Drienovská et al.^258^ have also employed the ncAA 2-amino-3-(8-hydroxyquinolin-3-yl)propanoic acid **(86, HQA-1)** for the creation of a new metal-binding site in LmrR. 8-Hydroxyquinoline is a versatile metal-binding ligand and has been used in various Cu^II^, Zn^II^ and Rh mediated reactions.^259^ Using an engineered *Mj*Tyr OTS,^260^**HQA-1** was incorporated at positions V15 and M89 in the LmrR protein scaffold. Both mutants showed good affinity for Cu^II^, Zn^II^, and Cp*Rh^III^. The LmrR_V15/M89**HQA-1**_Cu^II^ constructs were subsequently evaluated in the previously described FC alkylation reaction and enone hydration. In both cases, LmrR_V15**HQA-1**_Cu^II^ displayed high conversions but without enantioselectivity. Similar results were obtained for free Cu^II^(NO_3_)_2_, suggesting the **HQA-1**-Cu^II^ complex might be solvent exposed, with minimal interactions provided by the protein scaffold. In contrast, LmrR_M89**HQA-1**_Cu^II^ showed low conversions and moderate enantioselectivities in these reactions (Figure 15b,c), indicating positioning of the catalytic moiety inside the LmrR pocket. Next to that, the variants complexed with Zn^II^ were found to be active in hydrolyzing the amide bond between a model tripeptide and *p*-nitrophenylalanine. Unfortunately, no catalysis could be performed with variants complexed with Cp*Rh^III^, which was attributed to the limited number of free coordination sites when bound to **HQA**-1. Overall, the authors demonstrated for the first time the use of **HQA**-1 as metallocatalytic residue, providing a new platform for future ArM design.

In an effort to create an artificial deallylase, Stein and co-workers^261^ incorporated four different metal-binding ncAAs (**HQA-1**, (*S*)-2-amino-3-(8-hydroxyquinolin-5-yl)propanoic acid **(87, HQA-2)**, **BpyA**, and (*S*)-2-amino-3-(4-hydroxy-3-(1H-pyrazol-1-yl)phenyl)propanoic acid **(88, PyY)**) into the HaloTag protein.^262^ The four ncAAs were introduced in three different positions (F144, A145, and M175) in proximity of the binding cleft of HaloTag. Only the constructs harboring **HQA-2**(263) were evaluated in the allylic deamination of O-allyl carbamate-protected coumarin. A slight increase in turnover compared to HaloTag_WT was reported upon incubation with [(η^5^-C_5_H_5_)Ru(MeCN)_3_]^+^.

Jung and colleagues developed a series of artificial dicopper oxidases by incorporation of **BpyA** into a homohexameric acyltransferase from *Bacillus anthracis*.^264^ The intrinsic protein symmetry facilitated the formation of dicopper sites by placing **BpyA** moieties in close proximity to each other. **BpyA** was incorporated at four distinct positions (F120, K143, S144 and N300), of which variant N300**BpyA** exhibited the highest ascorbate oxidation rate. In general, oxidation activity was found to be inversely correlated to the distance between **BpyA** moieties, likely due to the possible formation of dinuclear copper sites. Furthermore, depending on the location of **BpyA** incorporation, discrete reactivities of the four constructs with dioxygen and hydrogen peroxide were observed. These results highlighted the application of **BpyA** for the creation of dinuclear copper sites and potential use for the development of ArMs for multiproton/electron-mediated redox reactions.

Overall, these studies demonstrate that by incorporation of ncAAs into proteins, it is possible to incorporate metal-binding moieties with first coordination spheres not available in nature. Together with a variable second coordination sphere determined by the protein scaffold and positioning of the metal complex, these characteristics allowed the creation of ArMs exploiting metal reactivity not usually observed in natural systems. ArMs featuring ncAAs directly applied as metallocatalytic residues have been mainly based on **BpyA** in combination with copper. Examples using metal-binding ncAAs in combination with second and third row transition metals are strikingly underrepresented in this category, which is likely related to the limited diversity in the first coordination spheres available in the ncAAs used so far; that is, there is a notable absence of OTSs for incorporation of ncAAs containing ligands typically used for these metals, such as phosphine or N-heterocyclic carbene. Toward this direction, Duan et al.^265^ reported the genetic incorporation of (2*S*)-3-(4-(2-(l4-boraneyl)-2,5-dihydro-1H-phosphol-1-yl)phenyl)-2-aminopropanoic acid **(89, P3BF)**, a ncAA containing a borane-protected phosphine into the LmrR scaffold. However, it could only be incorporated in proteins in protected form and both deprotection and metalation with Pd required long reaction times. No catalysis was reported for this artificial metalloprotein.

The use of ***p*AzF** as bio-orthogonal handle allows for more flexibility in this case, with the possibility to conjugate different transition metal complexes, yet with the disadvantage of an extra post-translational step and the need for a large BCN-linker. Evolution and *in vivo* application of ArMs featuring non-natural metals are, in general, impeded due to the inevitable post-translational binding or modification that is necessary for catalytic activity, which can also be observed from the limited number of evolution campaigns that have been reported for ArMs featuring ncAAs.

#### 5.2.3 Myoglobin-Based ArMs Featuring ncAAs

Myoglobin (Mb) is a single domain heme protein mainly located in vertebrates’ muscles. It is involved in oxygen storage and transport and some promiscuous nitrite reductase and peroxidase activities.^266−268^ Mb can be obtained in large quantities by recombinant expression in *E. coli* and has been extensively studied and characterized. Due to its attractive structural features and promiscuous activities related to the heme cofactor, Mb has been greatly targeted for enzymatic purposes.^269,270^ A relatively large variety of ncAAs have been employed for the modulation and creation of Mb-based metalloenzymes, and these have been classified here as ArMs. A distinction is made between Mb-based ArMs featuring ncAAs that have been constructed as a functional model of existing metalloenzymes and Mb-based ArMs that have been engineered toward new-to-nature reactivity.

##### 5.2.3.1 Myoglobin as Functional Model

**Figure 16:**
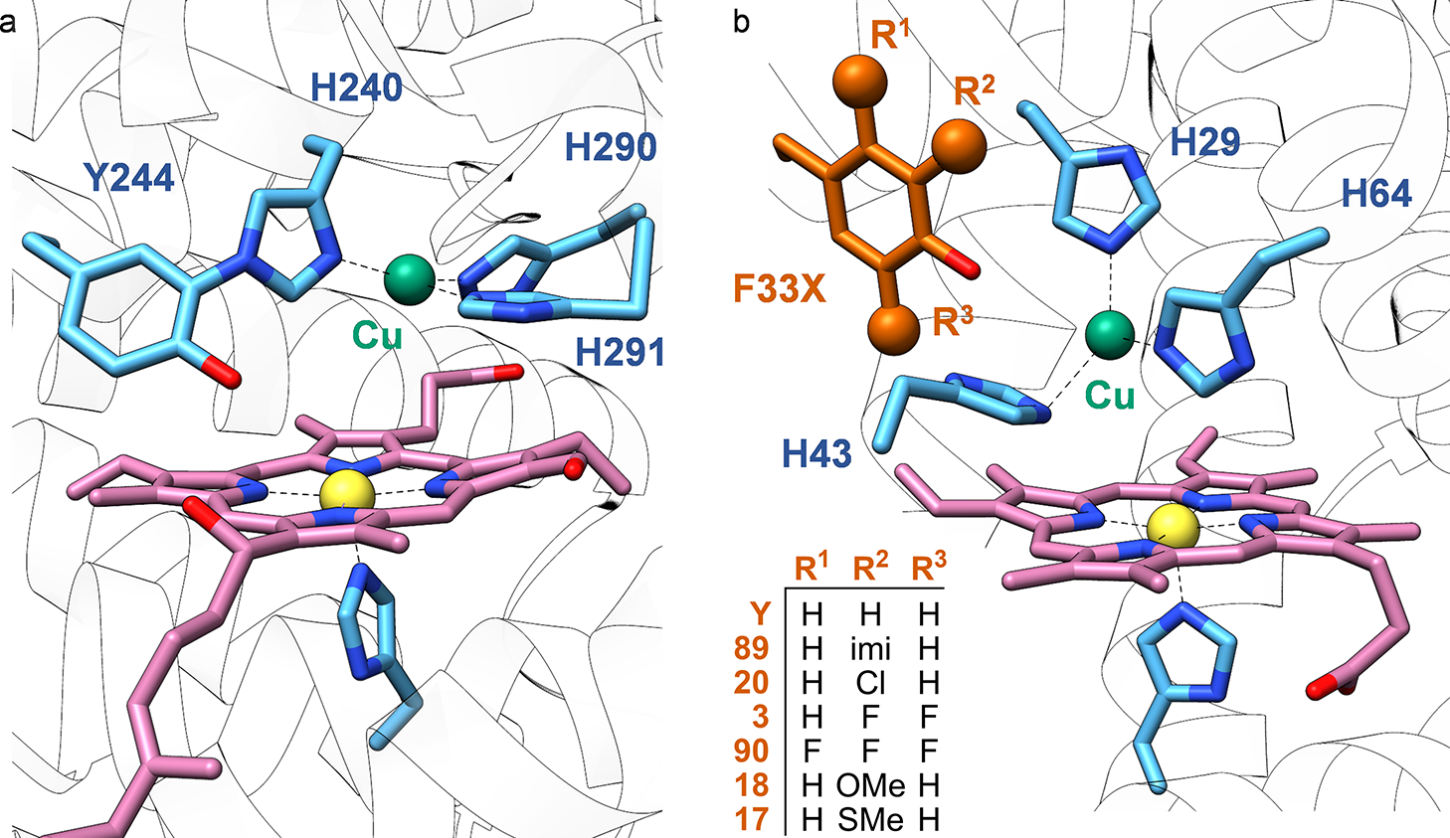
(a) Crystal structure of cytochrome c oxidase (PDB 1OCR) showcasing the post-translational tyrosine-histidine cross-link between Y244 and H240 in the Cu_B_ site. (b) Structural model of Cu_B_Mb_F33X (adapted from the crystal structure of Cu_B_Mb_F33Y, PDB 4FWY), used as functional model for HCOs by genetic incorporation of ncAAs at position F33X (depicted in orange).

The small size and relatively facile recombinant production of myoglobin have made it a suitable scaffold for the construction of functional models mimicking existing heme proteins. Heme-copper oxidases (HCOs) are responsible for creating an efficient proton gradient across the mitochondrial or cytoplasmic membrane at the end of the respiration pathway, by efficiently catalyzing the reduction of O_2_ to H_2_O without the undesired formation of ROS, such as peroxides and superoxide compounds. A unique conserved characteristic of HCOs is a post-translational modification in their reactive site known as the tyrosine-histidine cross-link (Figure 16a). This distinct feature has been suggested to affect the p*K*_a_ values of tyrosine and imidazole side chains, facilitating proton delivery and radical formation.^271,272^ HCOs are multiunit membrane proteins that are difficult to produce on a large scale. To gain a better insight into the role of the tyrosine-histidine cross-link, Lu, Wang and co-workers selected Mb as a host for the construction of a functional model of HCOs by genetically incorporating (*S*)-2-amino-3-(4-hydroxy-3-(1H-imidazol-1-yl)phenyl)propanoic acid (**90**, **imiY**), a ncAA that mimics the cross-linked tyrosine-histidine ligand, at position F33 (Figure 16b).^273^ A mutant *Mj*Tyr OTS was evolved for the specific response of the TAG codon and **imiY** incorporation, obtaining isolated yields 5-fold less than those for the parent protein. Further modification of this variant was the creation of a Cu^II^-binding spot, essential for the HCOs mimic oxidase activity, by the L29H mutation. The artificial mutant, Cu_B_Mb_F33**imiY**, showed selective O_2_-reduction activity of 2 O_2_/min (∼150-fold less than native HCOs) at >1000 turnovers and generating <6% of ROS. When compared to the values obtained from Cu_B_Mb_F33Y, a variant without the cross-link, the functional model outperformed 8- and 3-fold in selectivity (ROS formation) and catalytic turnover, respectively, confirming that the cross-linked scaffold plays an active role in optimal reduction of molecular oxygen to water.

In a following study, Wang, Lu and collaborators analyzed the function of the conserved tyrosine residue cross-linked to histidine in HCOs by genetically incorporating tyrosine derivatives ***m*ClY**, **(3,5-F**_**2**_**)Y**, (*S*)-2-amino-3-(2,3,5-trifluoro-4-hydroxyphenyl)propanoic acid (**91**, **(2,3,5-F**_**3**_**)Y**), and deuterium ***m*ClY** (**92**, ***m*ClDY**) in the active site residue Y33 of an analogous functional oxidase Mb model *via* SCS (Figure 16b).^274^ An inverse linear dependence of oxidase activity with the increasing p*K*_a_ values of the artificial phenol groups was observed. This trend supports the general role of tyrosine as an electron and proton shuttle to facilitate O–O bond cleavage and H_2_O formation. Moreover, the Cu_B_Mb_F33***m*ClY** mutant showed more than 1200 turnovers in O_2_ reduction, a 2.4-fold increase compared to the Cu_B_Mb_F33Y activity and in a similar range as the values obtained from their previous work.^273^

Later, the same research group was able to incorporate ***m*MeOY** in an identical Mb model.^275^ For this, parallel evolutionary campaigns of tyrosine phenol lyase (TPL) and a *Mj*Tyr OTS for the production and successful incorporation of ***m*MeOY**, respectively, were conducted. The new variant, carrying a ncAA with 179 mV lower redox potential but similar p*K*_a_ as tyrosine, was also able to catalyze O_2_ reduction with a 2.2-fold rate increase compared to the parent protein, suggesting that a fine control of the electron donating ability of a tyrosine residue in the active site is important for oxidase activity.

A tyrosine-cysteine cross-link has also been found as a conserved motif in diverse metalloenzymes, including the iron-dependent cysteine dioxygenase and *Thioalkalivibrio nitratireducens* cytochrome c nitrite reductase (TvNiR). Like the tyrosine-histidine-His cross-link, its functional significance has been intensively examined, suggesting that it modulates the proton and electron flow during the enzyme activity.^276,277^ Wang and co-workers developed a new *Mj*Tyr OTS that could incorporate ***m*SMeY**, a ncAA mimic of the tyrosine-cysteine motif, into a functional model of TvNiR in sperm whale Mb.^92^ Furthermore, a TPL mutant that could efficiently catalyze the synthesis of ***m*SMeY** from a phenol precursor was also evolved. The ***m*SMeY**-Mb variant, harboring the mutations F33***m*SMeY** and L29H, showed hydroxylamine to ammonia reduction activity with a *k*_cat_ 4-fold higher than that of the corresponding variant without ***m*SMeY**. This increase in catalytic activity provides evidence that the thioether substitution on the tyrosine residue can enhance enzyme activity as well, in resonance with the previously mentioned cross-linked ncAA mimic in active Mb models.

Chand et al. genetically engineered a Mb mutant incorporating the redox active *m*-aminotyrosine (**93**, ***m*AmY**) at distal position H64 to mimic the conserved distal histidine/arginine/Arg pair observed in horseradish peroxidases.^278^ The resulting variant, Mb_H64***m*AmY**, exhibited a 9- and 81-fold increase in activity toward thioanisole and benzaldehyde oxidation in the presence of H_2_O_2_, respectively, when compared to Mb_WT. This increase in reactivity was in accordance with the observed 92 mV increase in reduction potential of the mutant compared to the parent protein. In a follow-up study, Chand and collaborators incorporated *****L***-DOPA** at H64. The Mb_H64(*****L***-DOPA**) variant, in turn, showed 10- and 54-fold higher turnover rates for the same substrates, respectively.^279^

**Scheme 8:**
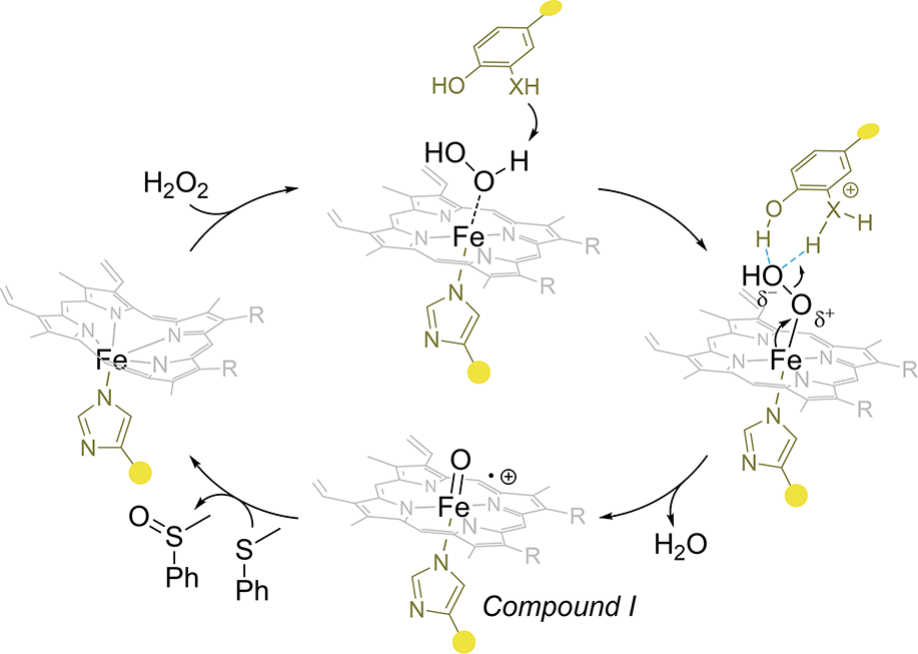
Plausible Toles of ***m*AmY** and ***L*-DOPA** in Compound I Formation and Thioanisole Oxidation by Mb_H64**ncAA** X = O (**49**), NH (**92**).

Within these ArMs, the ncAAs ***m*AmY** and ***L*-DOPA** would be providing a “pull-effect” by acting as a proton shuttle, where the functional groups -OH and -NH_2_ participate in hydrogen bond formation with the incoming molecules of H_2_O_2_, as well as ion stabilizers, to compensate for the charge density built up at the distal H_2_O_2_-bound ferric heme site (Scheme 8). In such a scenario, the incorporated ***m*AmY** and ***L*-DOPA** would promote a heterolytic pathway for the O-O bond scission in H_2_O_2_ toward the formation of the high valent ferryl-oxo species Compound I, thus accelerating the oxidation process. Interestingly, a Soret absorbance at 410 nm in the spectra of Mb_H64[***m*AmY**/(**L-DOPA**)] in the presence of H_2_O_2_ indicated that the ncAAs in the heme pocket had made the host Mb more resistant to oxidative degradation. Nevertheless, their contribution in activity for the oxidation of thioanisole is still greatly below the 200-fold increase obtained with the double mutant Mb_F42H_H64L.^280,281^ A similar approach by employing ***p*AmF** at different distal positions to enhance bacterial P450 (CYP102A1) monooxygenase activity has been reported by Kolev et al. (Section 3.1.3).^142^

##### 5.2.3.2 Myoglobin Engineering toward New-to-Nature Reactivity

**Figure 17:**
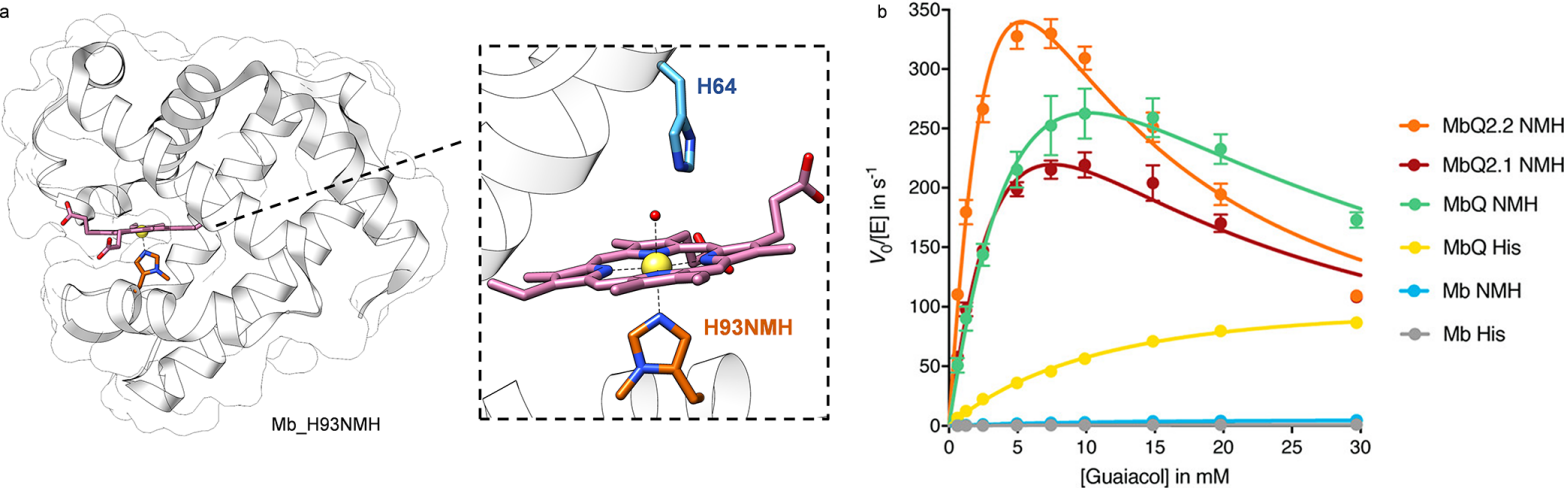
(a) Crystal structure of Mb **NMH** (PDB 5OJ9), depicting the heme-binding site and **NMH** incorporated at position H93. (b) Michaelis–Menten plots with guaiacol as substrate for Mb variants obtained throughout directed evolution. Reproduced with permission from ref (282). Copyright 2018 American Chemical Society.

Groundwork toward engineering new catalytic activities in Mb was laid down by Pott, Hayashi, et al., who augmented the relatively low promiscuous peroxidase activity of sperm whale Mb by recombinant replacement of the proximal ligand H93 (Mb His) with ncAA **NMH** (Mb **NMH**) (Figure 17a).^282^ This subtle substitution of the first coordination sphere ligand caused drastic changes to the protein properties, including a +74 mV increase in the heme redox potential, disruptions of H-bonds in the vicinity of the heme cofactors, and the observation of a direct transition to the neutral ferryl-oxo heme species Compound II with no evidence of Compound I accumulation. In conjunction, these variants exhibited a 3.7-fold increase in *k*_cat_/*K*_M_ oxidation of guaiacol when compared to Mb His, in agreement with their previous study in APX2.^149^ Mb His and Mb **NMH** were then improved by introducing four mutations near the active site (T39I, R45D, F46L, I107F; MbQ variants) on positions previously described as hot-spots for peroxidase activity enhancement.^283^ As a result, MbQ **NMH** showed an ∼140- and ∼7.1-fold boost in activity compared to the parent and MbQ His, respectively. Further optimization of the ArM based on the peroxidase activity on Amplex^TM^ Red was next pursued *via* directed evolution. This afforded MbQ2.1 **NMH** (additional substitutions: I28T, D45G, K63E, V68L, T95A, Y103H, K140T) and MbQ2.2 **NMH** (additional substitutions: V21A, I28T, D45G, K63E, T67A, T95A, K140T), both presenting ∼10-fold increase in activity compared to MbQ **NMH** (Figure 17b). Additionally, MbQ2.2 **NMH** showed an ∼2.2-fold and ∼1140-fold enhancement in guaiacol oxidation activity compared to MbQ **NMH** and Mb His, respectively. Remarkably, this catalytic efficiency is superior to that of wild-type APX (∼13-fold) and slightly lower than that of horseradish peroxidase (∼1.8-fold). All the generated variants were then tested against a small scope of aromatic substrates, demonstrating for most of the cases that the genetically encoded **NMH** variants resulted in increased initial rates.

**Scheme 9:**
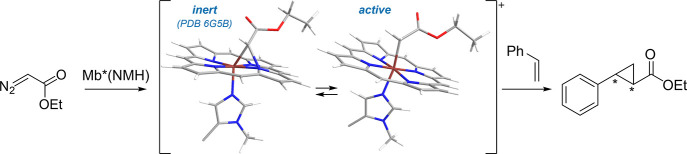
General Reaction Output for the Mb*(**NMH**) Catalyzed Cyclopropanation of Styrene with EDA.

Hayashi, Tinzl, et al. studied the enhancement of promiscuous activity of Mb by once again incorporating **NMH** through the stop codon suppression strategy at the proximal heme ligand in a Mb variant carrying the mutations H64V and V68A (Mb*),^284^ two modifications known to promote carbene transfer activity.^285,286^ The new mutant, Mb*(**NMH**), revealed similar activity and selectivity as Mb*(His) in the cyclopropanation reaction of styrene with ethyl diazoacetate (EDA) under reducing and anaerobic conditions. Yet, Mb*(**NMH**) was surprisingly active in the absence of the reducing agent dithionite, giving rise to full conversion in 1 h of reaction. Moreover, the **NMH** variant was oxygen tolerant, achieving ∼80% conversion after 7 h under air, a reaction condition where the histidine variant is almost inactive. X-ray crystallography of the designer enzyme under EDA excess, continuous flow EPR, and ^13^C NMR spectroscopy exposed a low-spin, stable intermediate with an unusual Fe-C-N(pyrrole) bridging configuration (Scheme 9), a structural feature reminiscent of described Fe^III^-porphyrin carbenoid complexes.^287,288^ Contrary to previous reports,^289,290^ the bridged intermediate proved to be mechanistically passive rather than inactivating. Quantum chemical calculations suggested that the bridged Fe^III^ complex is essentially inert toward styrene at room temperature, but in equilibrium with the corresponding end-on complex, shown to be the active intermediate for a productive cyclopropanation reaction.^291^

**Scheme 10:**
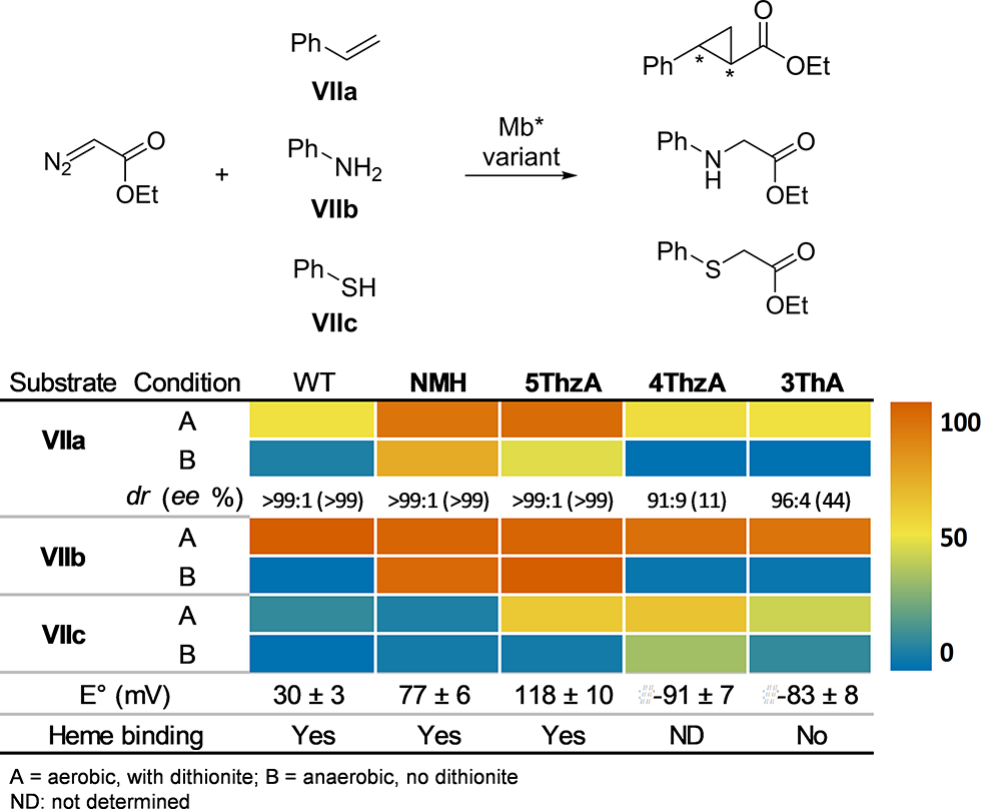
Yield Heatmap of Mb* Variants in Different Carbene Transfer Reactions under Aerobic and Anaerobic Conditions, with or without the Presence of Dithionite

In a subsequent work by Pott, Tinzl and collaborators, variants Mb*(**NMH**), Mb*(**5ThzA**), Mb*(**4ThzA**), and Mb*(**3ThA**) were produced and their performance tested in different carbene transfer processes with EDA.^292^ The incorporation of (*S*)-2-amino-3-(thiazol-5-yl)propanoic acid (**94**, **5ThzA)**, (*S*)-2-amino-3-(3H-1l3-thiazol-4-yl)propanoic acid (**95**, **4ThzA)**, and **3ThA**, three isosteric histidine analogues, was achieved by SCS, where two new *M. barkeri* PylRSs were developed for the effective insertion of the latter two ncAAs. The new mutants were successfully characterized by X-ray (PDBs: 6Z4T, 6Z4R), MS and UV spectra. Standard redox potential (E°) measurements indicated Mb*(NMH) (E° = 77 ± 6 mV)^284^ and Mb*(5ThzA) (118 ± 10 mV) to have more positive reduction potentials than the parent Mb* (30 ± 3mV),^284^ whereas the E_red_ values for Mb*(3ThA) (−83 ± 8 mV) and Mb*(4ThzA) (−91 ± 7 mV) were considerably lower. This property was reflected in the cyclopropanation, N-H, and S-H insertion reactions in the presence or absence of molecular oxygen and dithionite (Scheme 10). Where Mb*(**NMH**) and Mb*(**5ThzA**) excel in the former two reactions, Mb*(**4ThzA**) and Mb*(**3ThA**) variants performed better in the S-H insertion reaction when using thiophenol as the nucleophile. The fact that variants with the most positive reduction potentials were more efficient when using styrene as a nucleophile is consistent with computational studies indicating that more electron deficient iron-carbenoid complexes should speed up the concerted cyclopropanation reaction.^293^ On the other hand, the hydrogen atom transfer in the S–H insertion mechanism is the rate-determining step,^294^ which would correlate with the experimental observation that Mb* variants with lower reduction potentials outperform those with higher ones. In general, the new variants surpass Mb*(His) in carbene transfer under aerobic and/or dithionite-free conditions. The loss of *dr* and *ee* observed for Mb*(**4ThzA**) and Mb*(**3ThA**) was argued to be due to an altered coordination geometry of the heme cofactor, and presumably related to the lack of a proximal ligand-heme iron interaction observed in the resting state for the latter variant. These results suggest that **5ThzA** can also be used as a good mimic of histidine in proteins harboring proximal ligand–heme metal interactions, but a similar extrapolation for **4ThzA** and **3ThA** is not certain.

**Figure 18:**
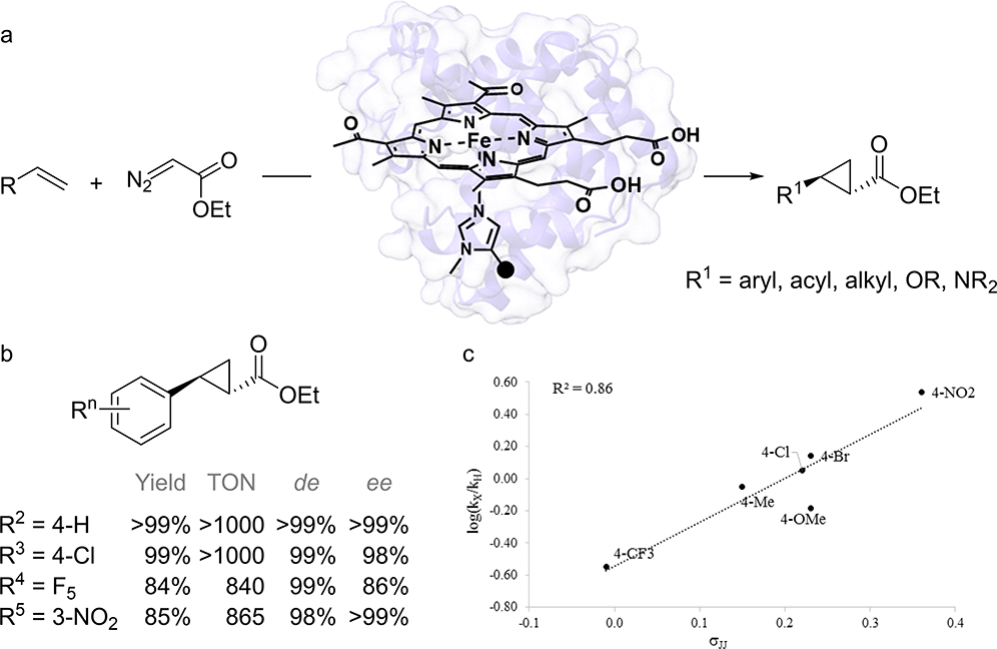
(a) General scheme for the cyclopropanation of a broad range of substituted alkenes by Carminati and Fasan (PDB 1JW8). (b) Selected scope examples. (c) Plot of log (*k*_X_/*k*_H_) vs σ^•^_JJ_ constant for Mb*(NMH)Fe(DADP). Adapted with permission from ref (295). Copyright 2019 American Chemical Society.

Carminati and Fasan employed the sperm whale Mb*(**NMH**) system with the non-native iron-2,4-diacetyl deuteroporphyrin IX [Fe(DADP)] for the design of a new type of carbene transferase.^295^ The incorporation of Fe(DADP) cofactor was effectively achieved by recombinant production, whereas NMH was introduced at the proximal position *via* amber stop codon suppression using a ncAA enriched media. Both substitutions individually resulted in an increase of the protein redox potential around 30 and 12 mV, respectively, but when combined in the same host a nearly 100 mV increase was observed, clearly suggesting a synergistic effect. Using this ArM, a broad range of substituted alkenes were efficiently cyclopropanated with high activity (up to >99% yield and >1000 TON) and selectivities (up to >99% *de* and *ee*) (Figure 18a,b). Notably, this designer ArM excels in reactions with electron-deficient alkenes, which are challenging substrates to functionalize when employing electrophilic Fisher-type metallocarbenes. In agreement with the results obtained by Hayashi, Tinzl, et al.,^284^ Mb*(**NMH**)[Fe(DAPD)] was tolerant to aerobic conditions and was catalytically active in the absence of an external reducing agent. As the ArM activity was inhibited by the presence of CO, it was suggested that the increased redox potential makes the resting state of this variant susceptible to reduction by EDA, producing a catalytically active ferrous species. Plots of the log (*k*_X_/*k*_H_) values with Hammet constants (σ^±^) showed no correlation for Mb*(**NMH**)[Fe(DAPD)], yet a good linear relationship was found with Jiang’s spin-delocalization substituent constants (σ^•^_JJ_), indicating the occurrence of a radical pathway (Figure 18c). Further experiments focused on reaction stereospecificity and involving radical spin trapping agents supported a stepwise radical-based mechanism, resembling the electron-deficient olefin cyclopropanation examples reported by the Zhang,^296^ DeBruin,^297^ and Deng^298^ laboratories. This methodology represents an illustrative example of a dramatic change in a reaction mechanism as a combined result of the introduction of a non-native amino acid and an artificial cofactor into the same protein host.

Moore and Fasan further studied the impact of the proximal ligand identity on the reactivity of Mb* as carbene and nitrene transferase.^299^ A series of proteinogenic (C, S, Y, D) and nonproteinogenic (***p*AmF**, **3ThA**, (*S*)-2-amino-3-(pyridin-3-yl)propanoic acid (**96**, **3PyA**)) amino acids were genetically introduced at position 93, and the resulting variants were evaluated in the intermolecular cyclopropanation of styrene with EDA and in the intramolecular C-H amination of 2,4,6-triisopropylbenzensulfonyl azide. Nearly all the mutants were found to have a cyclopropanation activity comparable to that of the parent protein, with Mb*(H93D) as the only variant capable of performing efficient catalysis under aerobic and nonreducing conditions in good yields and selectivity. This would indicate that D93 increases the redox potential of the heme cofactor sufficiently to be reduced with EDA. Moreover, carbon monoxide acted as an inhibitor, suggesting a mechanism that involves a ferrous ion as the catalytic center. In parallel, all the proximal ligand variants proved to exert the non-natural nitrene transferase activity similarly to the parent Mb*, albeit with no significant improvement under aerobic and/or nonreducing conditions.

The examples discussed in this section prove Mb as a privileged scaffold for reaction development and mechanism studies. The use of ncAAs to study the redox properties of the heme cofactor, at both the near and distant positions, and as new coordinating surrogates or post-transcriptional mimics, has been exploited greatly in the catalysis of carbene transfer reactions and oxidation processes, achieving great advances in the mechanistic understanding as well as in the reaction repertoire in biocatalysis. New ncAAs and their respective OTS development have great promise in bringing new types of transformations due to the relatively easy access of artificial metalloenzymes from Mb.

### 5.3 Photocatalytic Enzymes

Photocatalysis has emerged as a powerful technique to promote a myriad of chemical transformations by the use of light. Application of photosensitizers to harness the energy of photons and access reactive excited-state intermediates allows us to perform catalysis not generally accessible from the ground state.^300^ Photobiocatalysis is an approach that combines the unique reactivity accessible through photochemistry with the high activity and selectivity that biocatalysts offer under mild conditions. Strategies include the use of chemical photosensitizers to supply natural redox enzymes with high-energy electrons,^301^ but also the introduction of new reactivity into enzymes to create photobiocatalysts for abiological transformations.^302^ In this light, SCS has been applied to incorporate abiological photocatalytic moieties into protein scaffolds, leading to the creation of artificial photocatalytic enzymes. Moreover, while controlling enantioselectivity in photochemistry can be challenging,^303^ the protein scaffold has the potential to provide a chiral environment allowing triplet state enantioinduction for enantioselective photobiocatalysis. A distinction is made between photoenzymes in which the ncAA is used as bio-orthogonal handle to introduce non-natural photocatalytic moieties (section 5.3.1) and photoenzymes in which inherent metal-binding or photosensitizing properties of ncAAs are utilized (section 5.3.2).

#### 5.3.1 ncAA as Bio-orthogonal Handle

**Figure 19:**
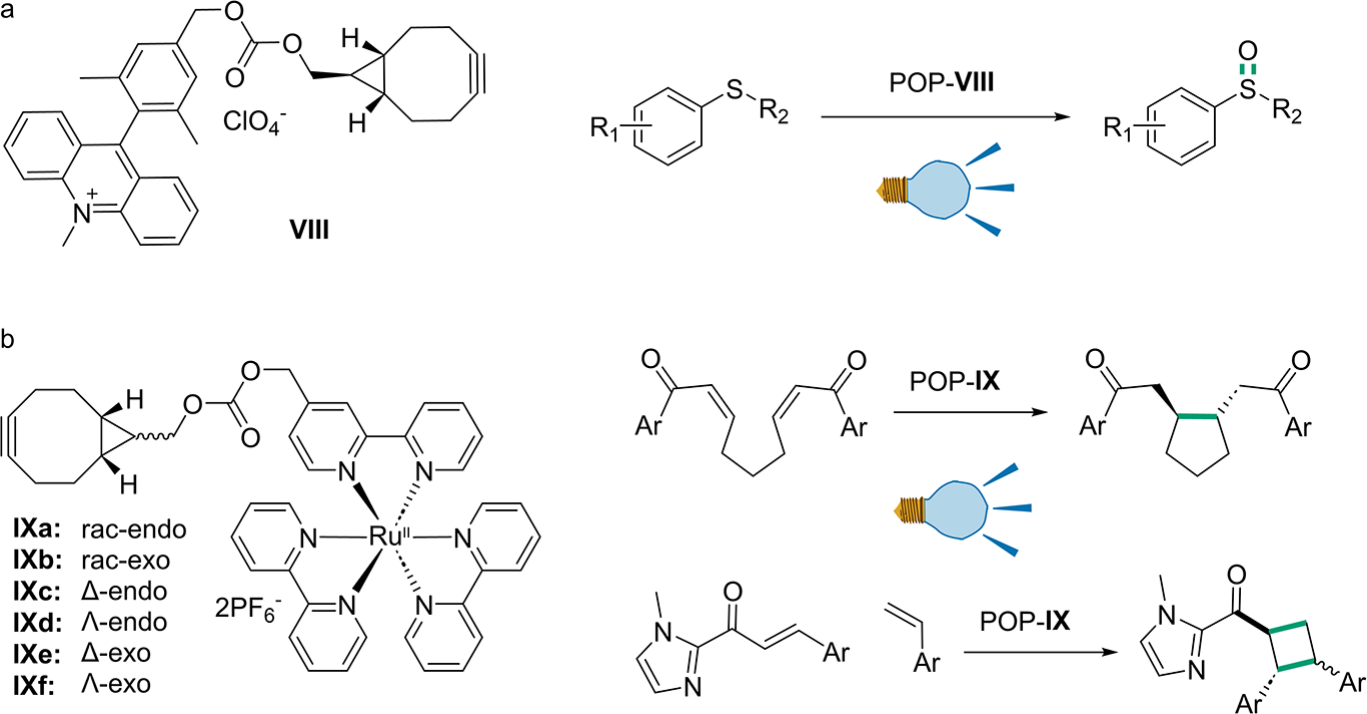
Prolyl oligopeptidase (POP)-based artificial photocatalytic enzymes constructed by genetic incorporation of ***p*AzF**, followed by SPAAC attachment of photocatalysts. (a) BCN-substituted Acr^+^-Mes (**VIII**) and POP-**VIII** catalyzed sulfoxidation of thioanisoles. (b) BCN-substituted Ru^II^(Bpy)_3_ cofactors (**IXa-f**) and POP-**IX** catalyzed reductive cyclization or [2 + 2] cycloaddition reaction.

Gu and co-workers^304^ created an artificial photocatalytic enzyme through incorporation of the ncAA ***p*AzF**(217) into prolyl oligopeptidase^240^ at position S477, followed by SPAAC attachment of the photocatalyst 9-mesityl-10-methylacridinium perchlorate (Acr^+^-Mes, **VIII**) (Figure 19a).^237^ Although coupling efficiency was modest, the construct showed activity toward the aerobic sulfoxidation of thioanisoles (Figure 19a) using visible light. However, racemic products were obtained and yields were lower than those obtained using free Acr^+^-Mes. Nonetheless, this study showcased the first example of a ncAA-based artificial photocatalytic enzyme that could act directly on an organic substrate.

Zubi and colleagues^305^ further explored the use of ***p*AzF** in the development of POP-based artificial photocatalytic enzymes by SPAAC attachment of a variety of BCN-substituted Ru^II^(Bpy)_3_ photocatalysts (Figure 19b, **IXa-f**) at five different active site positions of the POP_WT_ scaffold (53, 99, 326, 338, 477). While all positions could be bioconjugated with the Ru^II^(Bpy)_3_ cofactors, POP_53***p*AzF** showed the most efficient coupling. Covalent anchoring of the cofactors was found to increase the luminescence lifetimes in all variants. In addition to POP_WT_, Ru^II^(Bpy)_3_ cofactors were also anchored at 53***p*AzF** in POP_neg_, a variant where positively charged active site residues were removed and negatively charged residues were introduced. The covalent constructs were evaluated in two mechanistically different reactions, the first one being a reductive cyclization to generate a cyclopentane *via* single electron transfer (SET) and the second a [2 + 2] cycloaddition to give a cyclobutane *via* energy transfer (Figure 19b). Desired products were observed in all cases, generally with modestly increased conversions relative to the free Ru^II^(Bpy)_3_ photocatalysts. It must be noted though that similar yields for the reductive cyclization reaction were obtained for the photoenzyme and free photocatalyst. POP_neg_-53***p*AzF-IXf** and POP_WT_-53***p*AzF-IXd** increased reaction rates for the reductive cyclization and cycloaddition reactions, respectively, albeit without significant enantioselectivity.

#### 5.3.2 ncAA as Metal-Binding Ligand or Photosensitizer

**Figure 20:**
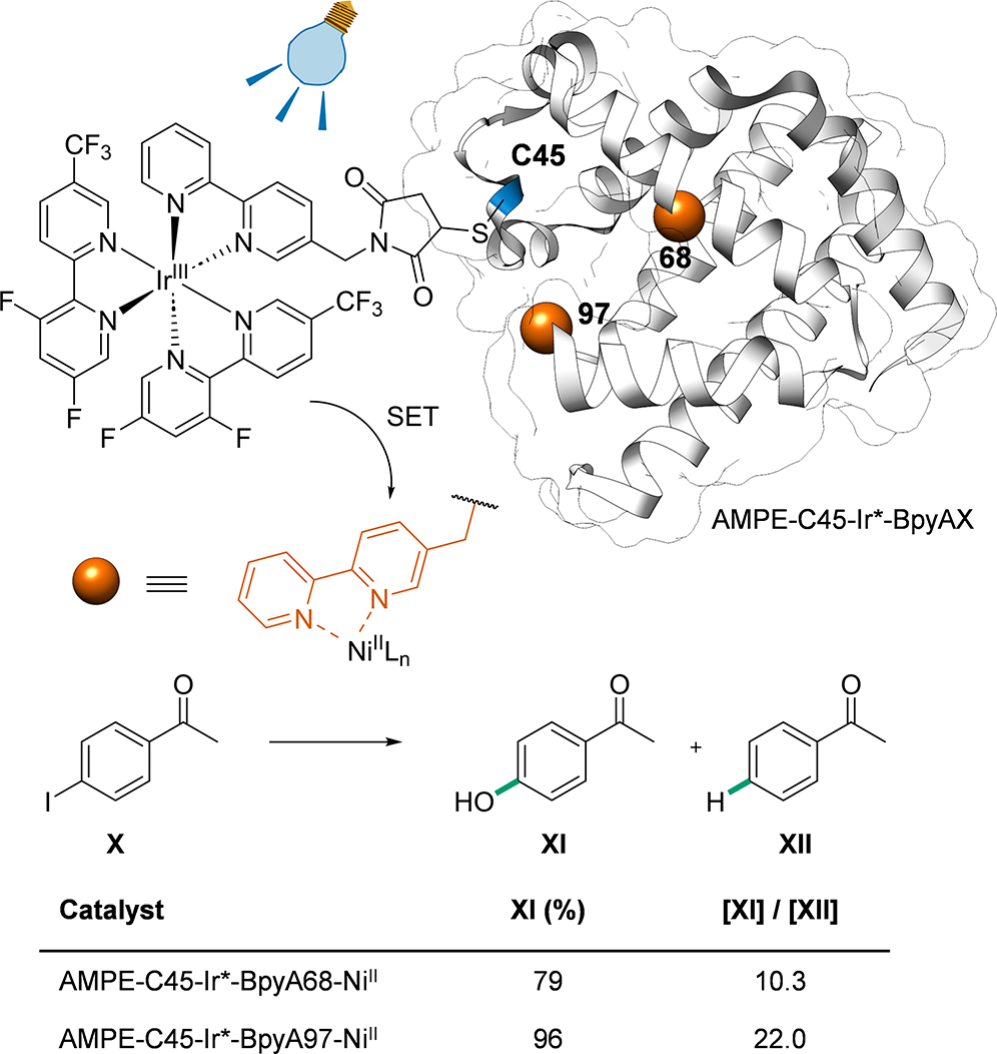
Artificial metallophotoredox enzyme (AMPE) constructed by genetic incorporation of metal binding ncAA **BpyA** into apo-myoglobin at position 68 or 97, followed by cysteine-maleimide conjugation of photosensitizer [Ir(dF(CF_3_)-ppy)_2_(dtbpy)]PF_6_ (Ir*) at C45. **BpyA** incorporation positions are depicted as orange spheres; C45 is depicted as blue ribbon (PDB 7YLK). Upon Ni^II^ complexation, different variants showed activity toward the photocatalytic hydroxylation of **X**, yielding product **XI**, next to dehalogenation product **XII**.

Lee and Song^306^ used genetically incorporated **BpyA** complexed with Ni^II^ in combination with a covalently linked iridium photosensitizer to create a photoenzyme capable of catalyzing the photocatalytic hydroxylation of aryl halides (Figure 20). Photoexcitation of the iridium photosensitizer followed by reductive quenching with a sacrificial reductant leads to the formation of Ir^II^. This strong reducing agent can subsequently reduce the nearby **BpyA**-Ni^II^ to form the **BpyA**-Ni^I^ species *via* SET, which is then able to catalyze the hydroxylation of aryl halide substrates. The design was based on an apo variant of myoglobin featuring three mutations (H64A, H93A, H97A) to remove heme and enlarge the hydrophobic pocket. Following **BpyA** incorporation at position V68, [Ir(dF(CF_3_)-ppy)_2_(dtbpy)]PF_6_ (Ir*) was covalently linked to C45 through cysteine-maleimide conjugation. Upon chelation of **BpyA** with Ni^II^, this resulted in the creation of the artificial metallophotoredox enzyme AMPE-C45-Ir*-**BpyA**68-Ni^II^, which showed activity in the photocatalytic hydroxylation of 4-iodo-acetophenone (Figure 20). However, unproductive dehalogenation of the aryl halide *via* direct SET from the Ir^III^ species was also observed. The catalytic activity was subsequently systematically optimized by probing the effect of covalently linking different iridium photosensitizers at different positions. Next to that, the effect of altered microenvironments around the Ni complex was probed by incorporating **BpyA** at different positions. Improved activities and selectivities were obtained with variant AMPE-C45-Ir*-**BpyA**97-Ni^II^, reaching 96% yield in the hydroxylation of **X**. Furthermore, activity was also observed for a range of differently substituted aryl-halides. This study showed that metal-binding ncAAs can be used in synergy with photosensitizers to create artificial photocatalytic enzymes.

**Figure 21:**
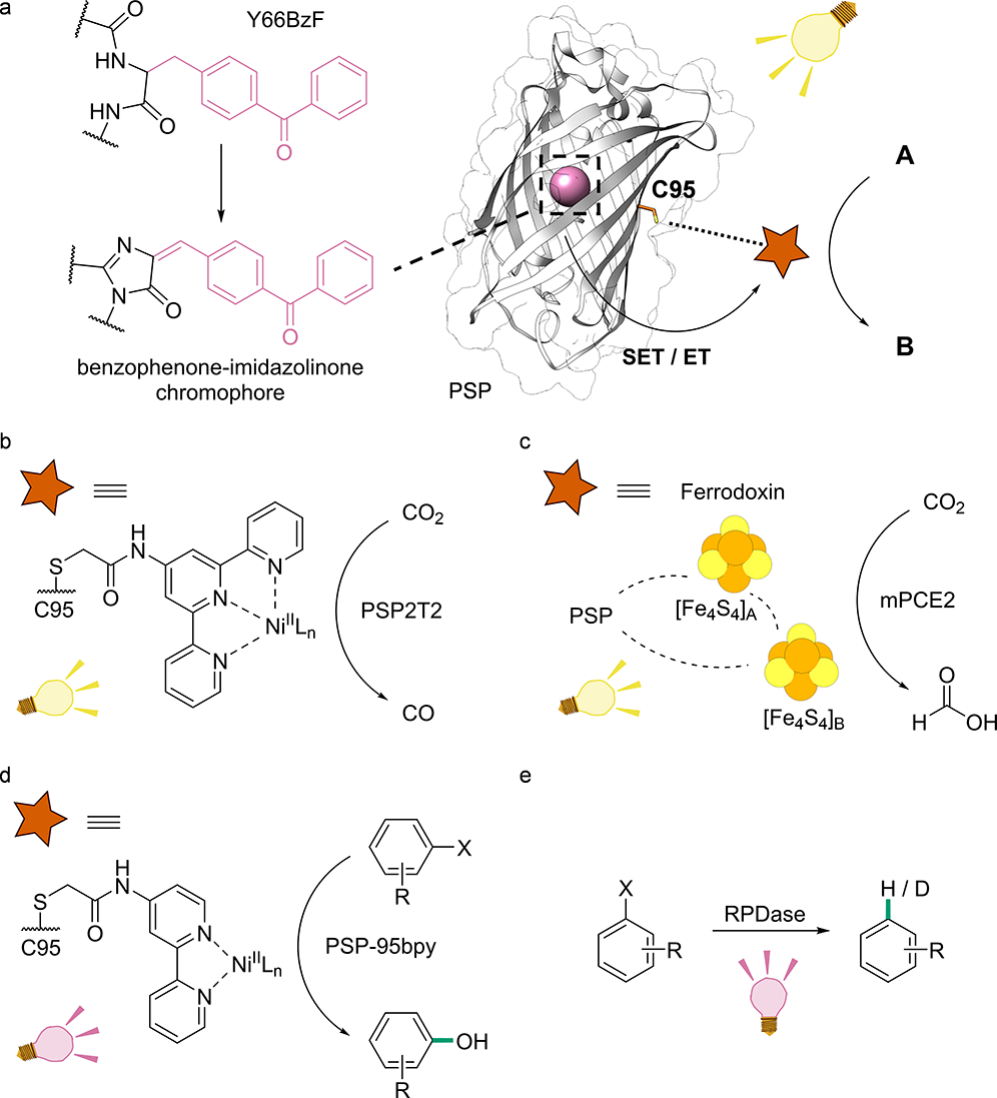
(a) Photosensitizer protein (PSP) constructed by genetic incorporation of ***p*BzF** at position Y66 of sfYFP (depicted as pink sphere, PDB 5YR3). Y66***p*BzF** undergoes an internal autocatalyzed conversion forming a conjugated benzophenone-imidazolinone chromophore. PSP served as the basis for the creation of a variety of artificial photoenzymes by combination with different metallocatalytic moieties (depicted as an orange star) *via* cysteine conjugation or genetic fusion. (b) Cysteine conjugation of a nickel-terpyridine complex to C95, resulting in PSP2T2, catalyzing the reduction of CO_2_ to CO. (c) Genetic fusion of circular permutated PSP to ferredoxin harboring two [Fe_4_S_4_] clusters, resulting in the construction of mPCE2, catalyzing the reduction of CO_2_ to formic acid. (d) Cysteine conjugation of a Ni^II^(bpy) complex to C95, resulting in PSP-95bpy, catalyzing the hydroxylation of aryl halides. (e) Use of PSP without metal-cofactor as photodehalogenase (RPDase) for hydrogenation or deuterodehalogenation of aryl halides.

In contrast to the indirect introduction of photocatalytic moieties, Liu and co-workers^307^ demonstrated the construction of an artificial photocatalytic enzyme by direct incorporation of a ncAA with inherent photosensitizing capabilities, namely ***p*BzF**.^308^ Inspired by the use of benzophenone as photosensitizer in organic photocatalysis,^300^ the authors converted superfolder yellow fluorescent protein (sfYFP)^309^ into a photosensitizer protein (PSP) by replacing Y66 with ***p*BzF** using an engineered *Mj*Tyr OTS.^308^ Similar to native sfYFP, PSP undergoes an internal autocatalyzed conversion forming a conjugated benzophenone-imidazolinone chromophore (Figure 21a). Fine-tuning of the PSP chromophore reduction potential by site-directed mutagenesis of close residues resulted in variant PSP2 containing two mutations (Y203D_H148E). Upon light absorption, PSP2 is converted into a long-lived triplet excited state PSP2*, which after reaction with a sacrificial reductant forms a super-reducing radical PSP2•. Combined with a cysteine conjugated nickel-terpyridine complex to C95 (Figure 21b), an artificial photocatalytic enzyme was created that catalyzed the reduction of CO_2_ to CO with a TON of 14 after 3 h and a quantum yield of 1.5%. Introduction of two tyrosine residues as potential local proton donors to facilitate electron transfer resulted in variant PSP2T2, which showed an increased TON of 25 and a quantum yield of 2.6%. Subsequent work reinstating Y203 in PSP2 resulted in variant PSP3, exhibiting faster photoinduced electron transfer. However, no catalysis was reported for this variant.^310^

Inspired by natural photosynthesis, Kang and colleagues^311^ genetically fused circular permutated PSP2 to the ferredoxin protein from *Clostridium acidurici*,^312^ a small protein harboring two [Fe_4_S_4_] clusters (Figure 21c). The resulting construct showed photocatalytic activity toward the reduction of CO_2_ to formic acid with a total turnover number (TTN) of 12. Fine-tuning of the distal [Fe_4_S_4_] cluster microenvironment and its reduction potential by site-directed mutagenesis gave variant mPCE2, containing two mutations (C8G_Y30N), exhibiting a 3-fold increase in TTN and a quantum yield of 1.43%. Further characterization suggested the directional electron transfer from photochemically reduced PSP2• to the distal [Fe_4_S_4_] cluster, which is subsequently reduced to the all-ferrous [Fe_4_S_4_]^0^ state, the active redox intermediate capable of reducing CO_2_ to formic acid.

Fu and co-workers^313^ coupled PSP to a Ni^II^(bpy) complex through cysteine conjugation at C95 to perform the photocatalytic hydroxylation of a variety of aryl halides (Figure 21d), obtaining the respective phenolic products in modest to good yields. Moreover, C-N cross-coupling using pyrrolidine or imidazole instead of water was also possible, albeit with modest yields. Transient absorption studies suggested that energy transfer facilitates the reductive elimination step to form the cross-coupling products. As probed by covalently linking the Ni^II^(bpy) complex at different positions, the distance between chromophore and metal catalyst was found to be important in order to maximize energy transfer while minimizing triplet excited state deactivation.

In other work, PSP was used as reductive photodehalogenase (RPDase) for hydrogenation of aryl halides without the need for a metal cofactor (Figure 21e).^314^ Furthermore, they showed that incorporation of alternative photosensitizer ncAA 3′-fluoro-BpA **(97, FBzF)** allowed hydrogenation using a light source with a slightly longer wavelength (405 nm vs 380 nm), albeit with modestly decreased yield. Using sodium formate as sacrificial reductant appeared to be a key for the hydrogenation activity. Mechanistic studies suggested a radical chain mechanism, in which reductive quenching of the triplet species by formate yields CO_2_^•–^, which can reduce the aryl halide substrate to form an aryl radical. Subsequent hydrogen abstraction from formate by the aryl radical then yields the arene product. Strikingly, using deuterated sodium formate allowed the deuterodehalogenation of aryl halides (Figure 21e). RPDase was shown to exhibit hydrogenation and deuteration activity for a broad array of aryl halides in good to high yields, including various pharmaceutically relevant molecules. Moreover, the authors showed that whole cells expressing RPDase can be used to perform the hydrogenation reactions with good yields, showcasing the first example of a fully genetically encoded photoenzyme allowing whole-cell photobiocatalysis. Although the oxygen-free conditions and use of UV light somewhat compromise the *in vivo* applications of RPDase, this study demonstrates a step forward in the application of photoenzymes in abiological transformations.

**Figure 22:**
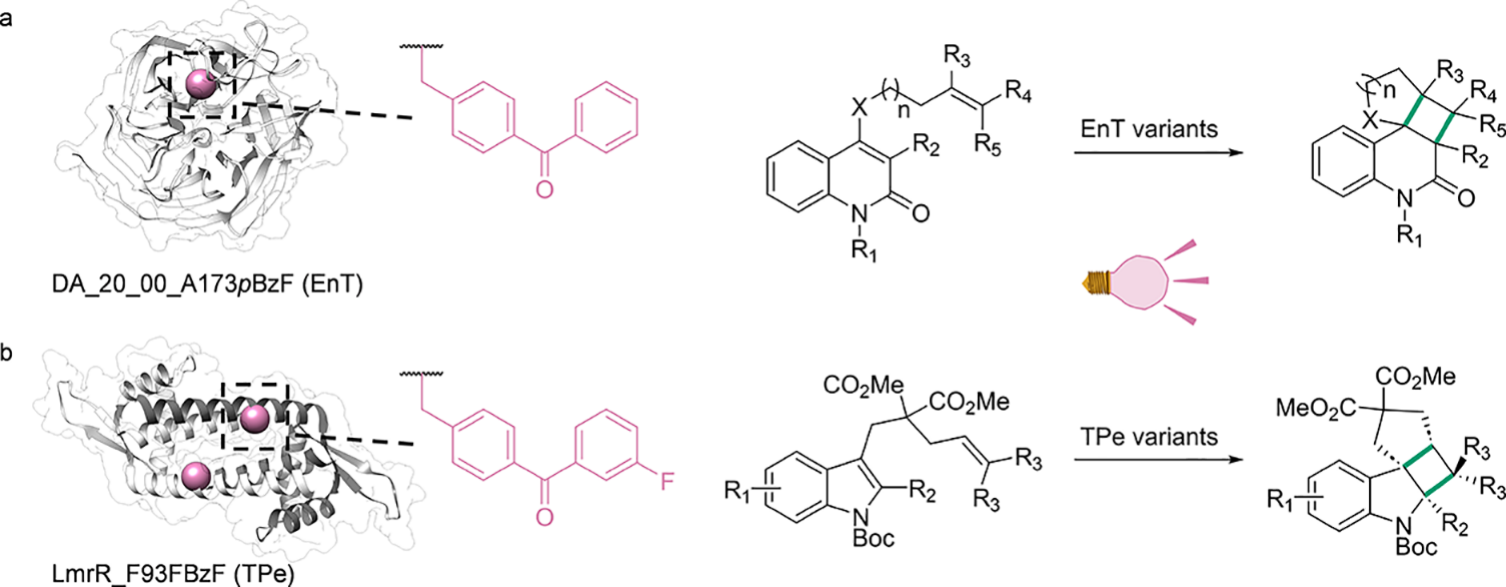
(a) Artificial photoenzyme constructed by genetic incorporation of ***p*BzF** at position A173 of the de novo designed DA_20_00 protein scaffold (depicted as pink sphere, PDB 7ZP5) creating EnT, catalyzing enantioselective intramolecular [2 + 2] cycloadditions of quinolone derivatives. (b) Artificial photoenzyme constructed by genetic incorporation of **FBzF** at position F93 of LmrR (depicted as pink spheres, PDB 3F8B) creating TPe, catalyzing enantioselective intramolecular [2 + 2] cycloadditions of indole derivatives.

Trimble and colleagues^315^ created an artificial photoenzyme catalyzing [2 + 2] cycloadditions by incorporation of ***p*BzF** into the *de novo* designed DA_20_00 protein scaffold.^316^ The starting design (EnT1.0) exhibited modest enantioselectivity in the intramolecular [2 + 2] cycloaddition of a quinolone substrate (Figure 22a), indicating that the protein scaffold is able to facilitate asymmetric induction of the triplet-state under mild conditions and in the presence of oxygen. The construct was then optimized to facilitate a subsequent directed evolution campaign using cleared lysate screening. As a result, variant EnT1.3 was found, which contained five mutations (M90A_Q149D_P196R_K225E_A229S) and exhibited significantly higher activity and enantioselectivity (up to 99% *ee*) toward a range of different quinolone substrates. Moreover, EnT1.3 also promoted intermolecular [2 + 2] cycloadditions and was shown to be amenable for optimization toward other quinolone derivatives.

In a simultaneous report, Sun and co-workers^317^ reported an LmrR-based artificial photoenzyme promoting enantioselective intramolecular [2 + 2] cycloadditions of indole derivatives (Figure 22b). ***p*BzF** was incorporated at position F93 in LmrR to create triplet photoenzyme TPe1.0. The initial construct exhibited modest activity in the model photocycloaddition using cell lysates under aerobic conditions, but no enantioselectivity was observed. Directed evolution led to the identification of TPe3.0, containing two mutations (W96L_M8L) with significantly improved enantioselectivity, yet for a limited set of substrates. Structural studies showed distinct interactions between ***p*BzF** and indole substrates, likely ensuring efficient energy transfer and enantioface differentiation. The design was further optimized by introduction of H-bond donor A11N and incorporation of **FBzF** instead of ***p*BzF** at F93 for potential H···F hydrogen bonds. This resulted in TPe4.0_**FBzF**, which showed superior enantioselectivities (up to 99% *ee*) for a range of *N*-substituted indole derivatives. Moreover, good enantioselectivities for substrates that were obtained with less than 90% *ee* could also be obtained by using other TPe variants and further enzyme optimization.

These studies show that artificial photocatalytic enzymes are able to promote highly enantioselective photochemical transformations through a triplet state energy transfer mechanism. The chiral protein environments of photoenzymes are an attractive feature compared to those of small molecule photocatalysts, facilitating enantioselective catalysis.^300,303^ For the majority of the examples, however, anaerobic conditions are necessary, which in combination with post-translational conjugations, controlled irradiation and potential photodamaging, make high-throughput screening and *in vivo* applications challenging. Nonetheless, it is also shown that, in some cases, artificial photocatalytic enzymes can be used under aerobic conditions, or even in a whole-cell fashion. The use of a ncAA as the photocatalytic residue itself has the most potential in this regard, as it is fully genetically encodable without the need for a post-translational conjugation or the introduction of a metal cofactor. Evolution of such artificial photoenzymes toward other valuable photochemical transformations is within the possibilities. So far, the scope of ncAAs used as photosensitizer in photobiocatalysis has been limited to ***p*BzF** and **FBzF**. Yet, incorporation of other ncAAs with photosensitizing properties such as the xanthone-resembling ncAA (*S*)-2-amino-3-(7-fluoro-9-oxo-9*H*-xanthen-2-yl)propanoic **(98, FXO)** reported by Liu et al.^318^ could expand the functionality and facilitate development of other artificial photocatalytic enzymes.

### 5.4 Discussion and Perspective on the Use of ncAAs in Artificial Enzyme Design

Being through conjugation or by making use of inherent organocatalytic, metal-binding, or photosensitizing characteristics, the incorporation of ncAAs has allowed the creation of artificial enzymes with catalytic functionalities not available to nature, expanding the biocatalytic repertoire of enzymes. The protein scaffold plays an important role in defining the chemical and chiral environment for the reaction, and incorporation of a ncAA at varying positions or proteins can lead to different reactivities and selectivities. Design of a rudimentary artificial enzyme with basal activity toward a desired reaction can be challenging, as it requires the right protein environment and catalytic machinery to be positioned in the right way. Advancements in computational techniques and deep-learning-based protein design tools show significant potential to aid in this process and open up new possibilities in, for example, the exploration of new protein scaffolds or *in silico* screening.^8,207,319^ Although it must be noted that modeling non-natural components such as ncAAs, especially when metallated, is not straightforward.^320^ The ncAA defines the type of chemistry or conjugation that can be performed. While many ncAAs can be incorporated using SCS, not all are relevant for performing catalysis. Next to that, due to the fact that most of the successful SCS methodologies are based on the *Mj*Tyr and *Mm/Mb*Pyl OTSs, the majority of ncAAs are tyrosine- or pyrrolysine-based derivatives. In this regard, the discovery and evolution of new orthogonal translation systems can aid the expansion of incorporable ncAAs with chemical functionalities relevant to the design of new ArEs. Basal activities of rudimentary artificial enzymes can be improved upon using directed evolution techniques. The throughput of such a campaign is partly dependent on how the ArE is assembled. Designs that require post-translational modifications or nonstandard reaction conditions such as an anaerobic environment significantly decrease the possibilities. On the other hand, designs in which the ncAA is used as catalytic residue have the potential to facilitate directed evolution and whole-cell or *in vivo* applications. Moreover, they provide opportunities for the design of biocatalytic cascades and combination with biosynthesis of the ncAA itself. Due to the nontrivial, expensive production, and generally lower activities of ArEs featuring ncAAs compared to natural enzymes so far, they are not yet viable alternatives for conventional chemical approaches. Yet, although there are hurdles to overcome, ArEs featuring ncAAs have great potential in bringing the field of biocatalysis further, especially when it comes to performing transformations that have no equivalent in nature. Progress in the field is fast, which is also demonstrated by the number of new manuscripts that appeared in the literature after submission of this review, describing new examples of ncAAs used in artificial enzymes.^232,235,257,321−326^ With increasing examples of ncAA-based ArEs catalyzing challenging transformations and applications in whole-cells or *in vivo*, it is a promising field that is maturing and steering toward chemical application of designer enzymes.

## 6 Conclusions and Outlook

Biocatalysis is showing great promise for organic synthesis, especially in the context of sustainability and green chemistry, by facilitating chemical reactions under mild conditions with high selectivity.^2,4,327,328^ Incorporating ncAAs makes it possible to introduce new functional groups, allowing us to progress beyond what is accessible in the natural world. This review summarized the state of the art regarding the contribution of ncAAs to biocatalysis. It is shown that ncAAs aid the improvement in the field, moving from a fundamental understanding of enzymes to their modification and paving the way for the design of new enzymes capable of catalytic chemistry that has no equivalent in nature. Initial research regarding ncAAs focused on using SPI as a strategy for global incorporation, which was especially exploited to study the effects on activity and stability. Nonetheless, using SPI limits the diversity of ncAAs that can be incorporated, as their structure should resemble cAAs. However, after the 2000s, SCS unlocked the potential of having higher control regarding choosing specific incorporation sites relevant to more rational designs. Further research on the combined use of both strategies would be of great interest to the scientific community.

A better understanding of the mechanism of enzymes paves the way toward more rational enzyme engineering. This is challenging, but ncAAs can be a suitable ally in studying the underlying mechanism of diverse enzymes. They allow the introduction of specific desired changes within the protein structure to fine-tune steric and electronic effects, p*K*_a_, or redox potential, thus contributing to our understanding of these factors in enzyme catalysis.^71,86,89,91,93,112^ Enzymes function because of a delicate interplay of their components, with even minor changes affecting their catalytic activity. In this sense, it is clear that the incorporation of ncAAs has, in most cases, an effect on the overall activity, selectivity, stability, and/or substrate scope of the enzyme. Some studies have shown the potential of ncAAs for activity improvement, augmenting classic “canonical” protein engineering.^137^ Besides, ncAAs are a promising alternative for developing enzyme assembly complexes with high control of linking sites/number and orientation, avoiding the disturbance of the active site of the enzymes as commonly observed in classical approaches. The biocatalytic application of these assemblies has yet to be explored further. Beyond applications for improving/altering existing enzymes, the design of tailored artificial enzymes capable of tackling challenging or new-to-nature reactions for diversifying the current scope of biocatalysis is desirable.

An important point to consider is the utility of the ncAA. Or, in other words, when is it worth using them? Introducing an abiological component to a natural design (or made from canonical sources) increases the enzyme design complexity to higher levels that require significant effort and care that need to be taken into consideration. Obviously, the use of ncAAs should be justified by the direct benefit delivered by the properties they confer, at least from a rational design point of view. Yet, as discussed for several of the examples throughout this manuscript, the reward of a ncAA is sometimes hard to predict and even makes their use questionable, at least for catalysis purposes. A good reminder is that genetically encoded ncAAs give rapid access to features that are not found in nature or, if they are, make access to them simpler. The advancement of this field, particularly in the last 5 years, has shown us that ncAAs not only bring within our reach the opportunity to translate synthetic chemistry features into a biological scaffold but also pave the way to discover new types of transformations that are not yet conceivable by conventional bio- or chemocatalysis.

The promise of using genetic code expansion for biocatalysis is clear. Nonetheless, many challenges need to be addressed in parallel for this technology to continue to flourish. Indeed, this is only possible with a parallel development of the required biological machinery for genetic code expansion. Currently, engineered versions of tyrosyl and pyrrolysyl OTSs are the most popular. However, despite the engineering, this can limit the structural diversity achievable for new desired ncAAs. Solutions include exploring new orthogonal tRNA/aminoacyl rRNA synthetase pairs,^329^ working toward improving the efficiency of the incorporation and overall yield of the obtained protein,^37,330−333^ developing new strategies for engineering existing OTSs, or discovering new ones more efficiently.^334−336^ Furthermore, it is of great interest to establish OTSs that are mutually orthogonal to each other to unlock possible dual catalysis, for which existing work is undergoing.^333,337−340^

Another challenge that should be addressed to make this technology applicable in the future is the need to reduce the price and complexity of producing the ncAAs. Currently, most of the ncAAs discussed in this review were obtained through chemical synthesis, sometimes involving a substantial number of synthetic steps. One alternative is the production of these amino acids through biosyntheses, some of which were discussed in this report.^155,263,275^ However, many more examples have already been reported for the biosynthesis of ncAAs^341−343^ using either of two strategies: by using enzymes *in vitro*(263,275) or by taking advantage of the cell itself for *in vivo* synthesis.^155^ The latter further increases the potential for *in vivo* synthesis and incorporation with some already existing examples,^211,344−347^ which would be especially promising for industrial applications.

Further, many well-established approaches for the computational study of enzymes cannot be applied for ncAAs containing enzymes due to different reasons, such as lack of parametrization and few preceding studies, being overall a pressing manner.^320,348^ Yet, currently the gap is slowly being filled with different studies contributing to ncAAs parametrization, OTS design, enzyme design (for example, incorporation site), etc.^49,244,349−352^ The development of computational methods will also play a significant role in identifying and designing new suitable scaffolds for ArEs. Similarly, the number of resolved crystal structures containing ncAAs, beyond the ones involving sfGFP, is to date limited to only a few examples published or deposited in the PDB.^112,188,235,244,257^

In conclusion, using ncAAs broadens the array of available tools, operating synergistically with established protein engineering methods, toward unlocking the new potential of biocatalysts. The encouraging results obtained to date suggest the promise of ncAAs in biocatalysis. Further improvement in incorporation methods, exploration of new structure/functional groups for ncAAs, and subsequent development of new orthogonal pairs, along with the advancement in of simpler and more efficient synthesis of ncAAs, ideally through *in vivo* biosynthesis, will further drive the progress in this field. In an ever-expanding field of biocatalysis, ncAAs are ensuring their relevance and utility in the years to come for creating enzymes endowed with unique properties.

## Supporting Information Available

Tables S1–S2: Abbreviations used in the text. Tables S3–S6: Tables with summaries of individual sections. (PDF)
