# Supplementary material for: Noncanonical Amino Acids: Bringing New-to-Nature Functionalities to Biocatalysis
Source: Chem Rev. 2024 Sep 27;124(19):10877–923. doi: 10.1021/acs.chemrev.4c00136 (PMC11467907; doi:10.1021/acs.chemrev.4c00136)
Supplement: Supplementary file 1 — cr4c00136_si_001.pdf [file cr4c00136_si_001.pdf]

Noncanonical Amino Acids: Bringing New-To-Nature Functionalities to Biocatalysis

Bart Brouwer,[†,§] Franco Della-Felice,[†,§] Jan Hendrik Illies,[‡,§] Emilia Iglesias-Moncayo,[‡,§] Gerard Roelfes*[,†] & Ivana Drienovská*[,‡]

† Stratingh Institute for Chemistry, University of Groningen, Nijenborgh 4, 9747 AG, Groningen, The Netherlands

‡ Department of Chemistry and Pharmaceutical Sciences, Vrije Universiteit Amsterdam, De Boelelaan 1105, 1081 HV, Amsterdam, The Netherlands

[§] B. B., F. D-F., J. H. I. and E. I-M. contributed equally

* Corresponding authors

E-mail: i.drienovska@vu.nl

E-mail: j.g.roelfes@rug.nl

Supporting Information

CONTENTS

Table S1: Abbreviations used throughout the review. ..…………………………………… S3

Table S2: Abbreviations for the ncAAs. ………………………………………………………S6

Table S3: Summary of studies discussed in Chapter 2. …………………………………….S8

Table S4: Summary of studies discussed in Chapter 3. ………………………………………S11

Table S5: Summary of studies discussed in Chapter 4. ………………………………………S13

Table S6: Summary of studies discussed in Chapter 5. ………………………………………S15

References. ……………………………………………………………………………………S19

Table S1: Abbreviations used throughout the review.

**Abbreviation**	**Meaning**
[Fe(DADP)]	Iron-2,4-diacetyl deuteroporphyrin IX
2OG	2-oxoglutarate
4CL	4-coumarate:coenzyme A ligase
4-HBA	4-hydroxybenzaldehyde
5-AND	5-androstene-3,17-dione
aaRS	Aminoacyl-tRNA-synthetase
AaSHC	*Alicyclobacillus acidocaldarius* squalene-hopene cyclase
Acr^{+}-Mes	9-mesityl-10-methylacridinium perchlorate
ADH1	Alcohol dehydrogenase I
ADH2	Alcohol dehydrogenase II
AKR	Aldehyde ketone reductase
AMPE	Artificial metallophotoredox enzyme
APX2	Ascorbate peroxidase
ArsC	Arsenate reductase
ArE	Artificial enzyme
ArM	Artificial metalloenzyme
BB	Back-to-back orientation
BCN	Bicyclo[6,1,0]nonyne
cAA	Canonical amino acids
CAP	Catabolite activator protein
CAT	Chloramphenicol Acetryltransferase
CDNB	1-chloro-2,4-dinitrobenzene
CLE	Cross-linked enzyme
CotKSI	KSI from *Comamonas testosteroni*
CuAAC	Copper catalyzed azide-alkyne cycloaddition
Cu^{II}phen	Cu(1,10-phenanthroline)$(NO_3)_2$
de	Diastereoisomeric excess
DHF	Dihydrofolate
DHFR	Dihydrofolate reductase
DKR	Diketoreductase
EDA	Ethyl diazoacetate
EG	Ethylene glycol
EST1	Carboxylesterase P1 *Sulfolobus solfataricus*
FC	Friedel-Crafts
FF	Face-to-face orientation
FC-EP	Friedel-Crafts alkylation/enantioselective protonation
FDH	Formate dehydrogenase
FtmOx1	Verruculogen synthase/fumitremorgin B endoperoxidase

Abbreviation	Meaning
GSH	Glutathione
GST	Glutathione S-transferase
HAT	Histone acetyltransferase
HCOs	Heme-Copper Oxidases
HMFO	5-Hydroxymethylfurfural oxidase
Ir*	$[Ir(dF(CF_3)\text{-}ppy)_2(dtbpy)]PF_6$
KerBL	Keratinase *Bacillus licheniformis*
KerPA	Keratinase *Pseudomonas aeruginosa*
KIE	Kinetic isotope effect
KlenTaq	*N*-terminally truncated version of DNA polymerase I from *Thermus aquaticus*
KSI	Ketostereoid isomerase
LmrR	Lactococcal multidrug resistance Regulator
LPa	*Pseudomonas alcaligenes* lipase
MA-EP	Michael addition/enantioselective protonation
Mb His	Mb proximal ligand H93
MBH	Morita-Baylis-Hillman
*Mb*Pyl OTS	*Methanosarcina barkeri* pyrrolysyl OTS
*Mm*Pyl OTS	*Methanosarcina mazei* pyrrolysyl OTS
MD	Molecular Dynamics
MDH	Mannitol dehydrogenase
mDHFR	Murine dihydrofolate reductase
ME	Methyl ester
metA	O-succinyl-transferase
MIC	Minimum inhibitory concentration
MjTyr OTS	*Methanocaldococcus jannaschi* tyrrosyl OTS
MTG	Microbial transglutaminase
Myoglobin	Mb
NBD-H	4-hydrazino-7-nitro-2,1,3-benzoxadiazole
ncAAs	Noncanonical amino acids
NTR	Nitroreductase
O-DCLE	Ordered dual CLEs
OE	Organocatalytic esterase
OPH	Organophosphate hydrolase
OTSs	Orthogonal translation systems
PCAF	p300/CBP-associated factor
PFE	*Pseudomonas* fluorescence esterase
PLA2	Pancreas phospholipase A2
POP	Prolyl oligopeptidase
PrAS	Aristolochene synthase from *Penicillium roqueforti*
PSP	Photosensitizer protein

Abbreviation	Meaning
PpKSI	KSI from *Pseudomonas putida*
PTE	Phosphotriesterase
R-ATA	R-amine transaminase
ROS	Reactive oxygen species
RPDase	Reductive photodehalogenase
SCS	Stop Codon Suppression
SET	Single electron transfer
sfGFP	Superfolder green fluorescent protein
sfYFP	Superfolder yellow fluorescent protein
siRNA	Short interfering RNA
SPAAC	Strain-promoted azide–alkyne cycloaddition
SPI	Selective Pressure incorporation
STS	Stilbene synthase
T4L	T4 lysozyme
TK	Transketolase
T_m	Melting temperature
TON	Turnover number
TPL	Tyrosine phenol lyase
$t_{1/2}$	Half-life
Trx	Thioredoxin
TrxR	Thioredoxin Reductase
TTL	*Thermoanaerobacter thermohydrosulfuricus lipase*
TTN	Total Turnover number
TvNiR	*Thioalkalivibrio nitratireducens* cytochrome c nitrite reductase
Twi	UDP-glucosyltransferase
VSE	Vibrational Stark effect
WT	Wildtype
ΔTEM-1	N-terminal truncated β-lactamase
λL	Phage lambda lysozyme
$\sigma^{\bullet}_{JJ}$	Jiang’s spin-delocalization substituent constants
ω-TA	ω-transaminase

Table S2: Abbreviations for the ncAAs discussed in this review.

Abbreviation	Name	Number
*m*FY	*m*-fluorotyrosine	1
*o*FY	*o*-fluorotyrosine	2
(3,5-F_2)Y	(*S*)-2-amino-3-(3,5-difluoro-4-hydroxyphenyl)propanoic acid	3
(2,3,5,6-F_4)Y	2,3,5,6-tetrafluorotyrosine	4
5FW	5-fluoro-L-tryptophan	5
*o*FF	*o*-fluorophenylalanine	6
*m*FF	*m*-fluorophenylalanine	7
*p*FF	*p*-fluorophenylalanine	8
*p*MeOF	*p*-methyoxyphenylalanine	9
(3,4-F_2)F	3,4-diflourophenylalanine	10
(3,4,5-F_3)F	3,4,5-triflourophenylalanine	11
*p*ClF	*p*-chlorophenylalanine	12
*p*tFMeF	*p*-trifluoromethyl-phenylalanine	13
*p*NF	*p*-nitrophenylalanine	14
NapA	2-napthylalanine	15
Bta	(*S*)-2-amino-3-(benzo[b]thiophen-3-yl)propanoic acid	16
*m*SMeY	(S)-2-amino-3-(4-hydroxy-3-(methylthio)phenyl)propanoic acid	17
*m*MeOY	(*S*)-2-amino-3-(4-hydroxy-3-methoxyphenyl)propanoic acid	18
(2,3-F_2)Y	(*S*)-2-amino-3-(2,3-difluoro-4-hydroxyphenyl)propanoic acid	19
*m*ClY	3-chloro-*L*-tyrosine	20
*p*AmF	*p*-aminophenylalanine	21
*p*CNF	*p*-cyanophenylalanine	22
*p*BiA	*p*-biphenylalanine	23
*ot*BuY	*o*-*tert*-butyl-tyrosine	24
*o*BrF	o-Bromophenylalanine	25
*o*ClF	ochlorophenylalanine	26
NMH	N-methyl-*L*-histidine	27
3ThA	(*S*)-2-amino-3-(thiophen-3-yl)propanoic acid	28
*p*BzF	*p*-benzoylphenylalanine	29
(2,3,4-F_3)F	2,3,4-triflourophenylalanine	30
*p*MeF	*p*-methylphenylalanine	31
(4(S)-F)P	4(*S*)-fluoroproline	32
(6F)W	6-fluoro tryptophan	33
Aha	azidohomoalanine	34
Nle	norleucine	35
*t*HP	*trans*-4-hydroxyproline	36
*c*HP	*cis*-4-hydroxyproline	37
*p*SeHF	(*S*)-2-amino-3-(4-hydroselenophenyl)propanoic acid	38
Hco	L-(7-hydroxycoumarin-4-yl)ethylglycine	39
Mco	L-(7-methylcoumarin-4-yl)ethylglycine	40
*p*AcF	*p*-acetyl-phenylalanine	41
*p*AcrF	*p*-acrylamido-phenylalanine	42
*p*AzF	*p*-azido-phenylalanine	43
*o*AllylY	*o*-allyl-tyrosine	44

*p*BrF	*p*-bromophenylalanine	45
*p*IF	*p*-iodophenylalanine	46
*p*AzMeF	*p*-azidomethylphenylalanine	47
OBnY	(*S*)-2-amino-3-(4-(benzyloxy)phenyl)propanoic acid	48
*p*AMMeF	(*S*)-2-amino-3-(4-(aminomethyl)phenyl)propanoic acid	49
L-DOPA	*L*-3,4-dihydroxyphenylalanine	50
TFL	5′,5′,5′-trifluoroleucine	51
TFM	trifluoromethionine	52
(4(*R*)-F)P	(4*R*)fluoroproline	53
HPG	homopropargylglycine	54
TAA	1,2,4-triazole-3-alanine	55
4AmW	4-aminotryptophan	56
4FW	4-fluorotryptophan	57
7AzW	7-azatryptophan	58
*m*IY	3-Iodo-tyrosine	59
*m*BrY	3-Bromotyrosine	60
AlocKOH	N^{ε}-allyloxycarbonyl-L-lysine	61
*p*BoF	*p*-boronophenylalanine	62
*p*NCSF	*p*-isothiocyanatephenylalanine	63
Nv	Norvaline	64
EtG	ethylglycine	65
OMetS	O-methyl serine	66
iL	(*S*)-2-amino-4-methylhexanoic acid	67
CpA	(*S*)-2-amino-3-cyclopentylpropanoic acid	68
*t*BuL	*tert*leucine	69
*p*PaF	*p*-propargyloxyphenylalanine	70
*p*tBuF	(*S*)-2-amino-3-(4-(*tert*-butyl)phenyl)propanoic acid	71
SetY	(*S*)-2-amino-3-(4-(2-mercaptoethoxy)phenyl)propanoic acid	72
SprY	(*S*)-2-amino-3-(4-(3-mercaptopropoxy)phenyl)propanoic acid	73
SbuY	(*S*)-2-amino-3-(4-(4-mercaptobutoxy)phenyl)propanoic acid	74
BetY	O-2-bromoethyl tyrosine	75
BprY	(*S*)-2-amino-3-(4-(3-bromopropoxy)phenyl)propanoic acid	76
BbtY	(*S*)-2-amino-3-(4-(4-bromobutoxy)phenyl)propanoic acid	77
AZL	N^{6}-[(2-azido ethoxy)carbonyl]-*L*-lysine	78
PaL	N^{ε}-(propargyloxy)-carbonyl-*L*-lysine	79
BCNK	N^{6}-[(1R,8S,9R)-bicyclo[6.1.0]non-4-yn-9-ylmethoxy]carbonyl-*L*-lysine	80
BpyA	dipyridylalanine	81
D-PyK	N^{6}-((D)-pyrrolidine-2-carbonyl)-*L*-lysine	82
L-PyK	N^{6}-((L)-pyrrolidine-2-carbonyl)-*L*-lysine	83
D-PiK	N^{6}-((S)-piperidine-2-carbonyl)-*L*-lysine	84
L-PiK	N^{6}-((R)-piperidine-2-carbonyl)-*L*-lysine	85
HQA-1	(*S*)-2-amino-3-(8-hydroxyquinolin-3-yl)propanoic acid	86
HQA-2	(*S*)-2-amino-3-(8-hydroxyquinolin-5-yl)propanoic acid	87
pyY	(*S*)-2-amino-3-(4-hydroxy-3-(1H-pyrazol-1-yl)phenyl)propanoic acid	88

P3BF	(2*S*)-3-(4-(2-(l4-boraneyl)-2,5-dihydro-1H-phosphol-1-yl)phenyl)-2-aminopropanoic acid	89
imiY	(*S*)-2-amino-3-(4-hydroxy-3-(1H-imidazol-1-yl)phenyl)propanoic acid	90
(2,3,5-F_3)Y	(*S*)-2-amino-3-(2,3,5-trifluoro-4-hydroxyphenyl)propanoic acid	91
*m*ClDY	deuterium *m*ClY	92
*m*AmY	*m*-aminotyrosine	93
5ThzA	(*S*)-2-amino-3-(thiazol-5-yl)propanoic acid	94
4ThzA	(*S*)-2-amino-3-(3H-1l3-thiazol-4-yl)propanoic acid	95
3PyA	(*S*)-2-amino-3-(pyridin-3-yl)propanoic acid	96
FBzF	3'-fluoro-BpA	97
FXO	(*S*)-2-amino-3-(7-fluoro-9-oxo-9*H*-xanthen-2-yl)propanoic acid	98

Table S3: Summary of studies discussed in Chapter 2 (Exploring Enzymatic Activity with Noncanonical Amino Acids). The table contains the protein host, the method of incorporation, the effect on activity or catalysis performed and the corresponding reference.

ncAA	Protein host	Method of incorporation	Activity/Catalysis	Ref.
*m*FY	GST (A1-1)	SPI	Influence over GSH stabilization	[1,2]
	FtmOx1	SCS	Influence over the oxidized product distribution in verruculogen biosynthesis	[3]
*o*FY	GST A1-1	SCS	Influence over GSH stabilization	[2]
(3,5-F_2)Y	GST A1-1	SCS	Influence over GSH stabilization	[2]
	FtmOx1	SCS	Influence over the oxidized product distribution in verruculogen biosynthesis	[3]
(2,3,5,6-F_4)Y	GST A1-1	SCS	Influence over GSH stabilization	[2]
5FW	3GST	SPI	Influence over product release for CDNB	[4]
*o*FF	PCAF; *Tetrahymena* general control non-derepressor 5	SPI	Influence on the overall packing and stability of the protein	[5]
	*Pvu*II restriction endonuclease from *Proteus vulgaris*	SPI	No change in specific activity	[6]
*m*FF	PCAF; *Tetrahymena* general control non-derepressor 5	SPI	Influence on the overall packing and stability of the protein	[5]
	*Pvu*II restriction endonuclease from *Proteus vulgaris*	SPI	~2-fold increase in specific activity	[6]
	CotKSI*	SPI	4-fold decrease in k_{cat} while maintaining the K_M for 5-AND	[7]

ncAA	Protein host	Method of incorporation	Activity/Catalysis	Ref.
*p*FF	AaSHC	*In vitro*	Influence over cation-π interactions in polycyclizations	8
	*Pvu*II restriction endonuclease from *Proteus vulgaris*	SPI	~0.5-fold decrease in specific activity and ~0.8-fold loss in conformational stability	6
	PCAF; *Tetrahymena* general control non-derepressor 5	SPI	Influence on the overall packing and stability of the protein	5
*p*MeOF	AaSHC	SCS	Influence over cation-π interactions in polycyclizations	8
	DKR from *Acinetobacter baylyi* ATCC 33305	SCS	Influence over the enantioselective reduction of 2-chloro-1-phenylethanone	9
	*Pvu*II restriction endonuclease from *Proteus vulgaris*	SPI	~0.5-fold decrease in specific activity and ~0.8-fold loss in conformational stability	6
	PCAF; *Tetrahymena* general control non-derepressor 5	SPI	Influence on the overall packing and stability of the protein	5
(3,4-F_2)F	AaSHC	*In vitro*	Influence over cation-π interactions in polycyclizations	8
(3,4,5-F_3)F	AaSHC	*In vitro*	Influence over cation-π interactions in polycyclizations	8
*p*ClF	Aristolochene synthase	SCS	Influence over cation-π interaction in polycyclizations	10
*p*tFMeF	Aristolochene synthase	SCS	Influence over cation-π interactions in polycyclizations	10
*p*NF	Aristolochene synthase	SCS	Influence over cation-π interactions in polycyclizations	10
NapA	Aristolochene synthase	SCS	Influence over cation-π interactions in polycyclizations	10
Bta	Trx	SCS	Influence over redox activity	11
*m*SMeY	OvoA	SCS	Influence over redox activity during ovothiol synthesis	12
*m*MeOY	OvoA	SCS	Influence over redox activity during ovothiol synthesis	13
(2,3-F_2)Y	FtmOx1	SCS	Influence over the oxidized product distribution in verruculogen biosynthesis	3
*m*ClY	PsKSI	SCS	Influence over the electric field/catalytic proficiency activity	14
	FtmOx1	SCS	Influence over the oxidized product distribution in verruculogen biosynthesis	3

ncAA	Protein host	Method of incorporation	Activity/Catalysis	Ref.
*p*AmF	L*Pa*	SCS	Influence in diastereo-selective hydrolysis activity of menthyl propionate mixtures	[15]
	FtmOx1	SCS	Influence over the oxidized product distribution in verruculogen biosynthesis	[3]
*p*CNF	L*Pa*	SCS	Influence over diastereo-selective hydrolysis activity of menthyl propionate mixtures	[15]
	Cyt c	SCS	Influence over the electric field/catalytic proficiency activity	[16]
	DKR from *Acinetobacter baylyi* ATCC 33305	SCS	Influence over the enantioselective reduction of 2-chloro-1-phenylethanone	[9]
*p*BiA	DKR from *Acinetobacter baylyi* ATCC 33305	SCS	Influence over the enantioselective reduction of 2-chloro-1-phenylethanone	[9]
*ot*BuY	DKR from *Acinetobacter baylyi* ATCC 33305	SCS	Influence over the enantioselective reduction of 2-chloro-1-phenylethanone	[9]
*o*BrF	L*Pa*	SCS	Influence over diastereo-selective hydrolysis activity of menthyl propionate mixtures	[15]
*o*ClF	L*Pa*	SCS	Influence over diastereo-selective hydrolysis activity of menthyl propionate mixtures	[15]
*p*SeHF	PTE	SCS	Influence over the hydrolysis rate activity	[17]
NMH	Alanine racemase from *Bacillus stearothermophilus*	SCS	Interruption of a hydrogen bond network activation mechanism	[18]
3ThA	Alanine racemase from *Bacillus stearothermophilus*	SCS	Interruption of a hydrogen bond network activation mechanism	[18]

Table S4: Summary of studies from Chapter 3 (Improving Enzymes with Noncanonical Amino Acids). The table contains the enzyme class, the enzyme used, the method(s) of incorporation, the ncAA(s) used in the approach, the amino acid(s) replaced, the overall effect and the corresponding reference. A comprehensive overview of articles influencing the stability of natural enzymes with ncAAs is given within the main manuscript.

**Enzyme class**	**Enzyme**	**Method(s) of incorporation**	**ncAA(s)**	**Amino acid(s) replaced**	**Effect on**	**Ref.**
**Transferases**	TK	SCS	*p*AmF, *p*CNF, and *p*NF	Y385, Y485	Activity	[19]
	R-ATA	SCS	*p*BzF, (2,3,4-F_3)F, *p*tFMeF, and *p*MeF	F86, F88	Activity & Selectivity	[20]
**Hydrolases**	TTL	SPI	(4(S)-F)P), *p*FF, and (6F)W	F, P, and W	Activity	[21]
	TTL	SPI	Aha, Nle, *t*HP, *c*HP, *m*FF, *p*FF, *m*FY, and *o*FY	M, P, F, and Y	Activity & Selectivity	[22]
	TTL	SPI	Nle	M	Activity	[23]
	TTL	SPI & SCS	*p*BzF, and Nle	M, D221	Activity	[24]
	*ar*PTE	SCS	*p*SeHF	Y309	Activity	[17]
	*ar*PTE	SCS	Hco and Mco	Y309	Activity	[25]
	TEM-1 β-lactamase	SCS	*p*AcF, *p*MeOF, *p*AcrF, *p*AzF, *o*AllylY, *p*BrF, *p*IF, *p*AzMeF, *p*BiA, *o*tBuY	D179, V216	Activity	[26]
	PFE	SCS	*p*BzF, *p*CNF, *p*AzF, *p*AmF, and NapA	Various	Activity & Selectivity	[27]
**Oxidoreductases**	P450 BM-3 TH-4	SPI	Nle	M	Activity	[28]
	CYP102A1 ($P450_{BM3}$)	SCS	*p*AmF, pAcF, OBnY, and NapA	A, L, F, V, and T	Activity & Selectivity	[29]
	NTR	SCS	*p*AmF, NapA, *p*BzF, *p*MeOF, *p*AmMeF, *p*MeF, *p*tFMeF and *p*NF	F124	Activity	[30]
	mDHFR	SCS	NapA	F31	Activity & Selectivity	[31]

Enzyme class	Enzyme	Method(s) of incorporation	ncAA(s)	Amino acid(s) replaced	Effect on	Ref.
	mDHFR	SPI & SCS	NapA and *p*BrF	F31	Selectivity	[32]
	APX	SCS	NMH	H163	Activity & Selectivity	[33]
	ADH2 H277	SCS	*L*-DOPA	D194, H198, H263, and H277	Selectivity	[34]

Table S5: Summary of studies from Chapter 4 (Enzymatic Assemblies Using Noncanonical Amino Acids). The table contains the ncAA(s) used, protein host(s), the method(s) of incorporation, the method of coupling, the overall effect on activity or catalysis and the corresponding reference.

ncAAs	Protein host	Method of incorporation	Bio-orthogonal coupling	Activity/Catalysis	Ref.
**One enzyme**					
Aha	TEV	SPI	CuAAC	Join two inactive peptide fragments to generate a catalytic active enzyme.	35
AZL		SCS			
HPG		SPI			
PaL		SCS			
BpyA	POP	SCS	-	Develop switchable enzymes that in the presence of a metal cannot perform its catalytic activity while recovering it upon removal.	36
	Photinus pyralis luciferase enzyme	SCS	-		
*p*AzF	AKR	SCS	SPAAC – linker (alkyne)	Development of cross-linked enzyme (CLE) which can be used for ketone reduction to obtain chiral alcohol with high yield and *ee*.	37
*p*PaF	ADH	SCS	CuAAC – linker (azide)	Generation of a hybrid material composed of the photocatalytic system to regenerate NADPH; this cofactor is then used for the catalysis of CLE_ADH.	38
**Two enzymes**					
*p*AzF	sfGFP	SCS	CuAAC-no linker	Protein conjugation of two enzymes.	39
*p*PaF	DHFR/sfGFP	SCS			
*p*AzF	GST	SCS	SPAAC - no linker	Protein conjugation of two enzymes.	40
BCNK	Maltose-binding protein				
AZL	GST				
*p*AzF	FDH/MDH	SCS	SPAAC – linker (alkyne)	Protein conjugation of two enzymes using linker and double click reactions. Under NADH limiting diffusion conditions, enzymes assembly performs better than a mixture of free enzymes.	41
*p*AzF	FDH/MDH	SCS	SPAAC – linker (alkyne)	Protein conjugation of two enzymes based on the orientation of active sites. More efficient NADH transfer is observed when active sites face each other.	42
*p*AzF	AKR	SCS	SPAAC – linker (alkyne)	Protein assembly to form dual enzymes CLEs (DCles). DCLES have higher yield and *ee* than the corresponding traditional CLEAs or free AKR.	43
*p*AzF	ADH				
*p*AzF	AKR	SCS	CuAAC	Assembly of ordered CLEs (O-DCLEs) for more precise spatial control to avoid no selective crosslinking in which AKR or ADH	44
*p*PaF	ADH				

ncAAs	Protein host	Method of incorporation	Bio-orthogonal coupling	Activity/Catalysis	Ref.
				aggregate with itself for more efficient catalysis.	
**Three enzymes**					
Aha	STS	SPI	SPAAC - linkers	Multi-enzymatic complex capable of performing a cascade reaction by assembling three different enzymes (STS, 4CL and Twi) use for synthesis and glycosylation of resveratrol	[45]

Table S6: Summary of studies from Chapter 5 (Artificial Enzymes Featuring Noncanonical Amino Acids). The table contains the protein host, the method of incorporation, post-translational modification or addition (if any), the effect on activity or catalysis performed and the corresponding reference.

ncAA	Protein host	Method of incorporation	Post-translational modification (includes cofactor addition)	Activity/Catalysis	Ref.
(3,5-F_2)Y	Mb	SCS	-	HCOs Tyr-His cross link mimic in redox activity	46
*m*SMeY	Mb	SCS	-	TvNiR Tyr-Cys cross link mimic in redox activity	47
*m*MeOY	Mb	SCS	-	HCOs Tyr-His cross link mimic in redox activity	48
*m*ClY	Mb	SCS	-	HCOs Tyr-His cross link mimic in redox activity	46
*p*AmF	Mb	SCS	-	Heme proximal ligand exchange in carbene and nitrene transferase activity	49
	LmrR	SCS	-	Catalytic residue in hydrazone formation reaction in an *in vivo* biocatalytic cascade	50
*p*BzF	sfYFP	SCS	Autocatalytic chromophore formation, and a cysteine conjugated Ni^{II}-terpyridine complex	Photosensitizer in an artificial photoenzyme catalyzing reduction of CO2 to CO	51
	sfYFP – ferrodoxin	SCS	Autocatalytic chromophore formation, and genetic fusion with ferrodoxin	Photosensitizer in an artificial photoenzyme catalyzing reduction of CO_2 to formic acid	52
	sfYFP	SCS	Autocatalytic chromophore formation, and a cysteine conjugated Ni^{II}(bpy) complex	Photosensitizer in an artificial photoenzyme catalyzing the hydroxylation of aryl halides	53
	sfYFP	SCS	Autocatalytic chromophore formation	Photosensitizer in an artificial photoenzyme catalyzing hydrogenation and deuteration of aryl halides	54
	DA_20_00	SCS	-	Photosensitizer in an artificial photoenzyme catalyzing enantioselective in the intramolecular [2+2] cycloadditions of quinolones	55
	LmrR	SCS	-	Photosensitizer in an artificial photoenzyme	56

ncAA	Protein host	Method of incorporation	Post-translational modification (includes cofactor addition)	Activity/Catalysis	Ref.
				catalyzing enantioselective in the intramolecular [2+2] cycloadditions of indoles	
*p*AzF	LmrR	SCS	Staudinger reduction to obtain *p*AmF	Organocatalytic residue in hydrazone formation reaction	57,58
	LmrR	SCS	Staudinger reduction to obtain *p*AmF	Organocatalytic residue in vinylogous Friedel-Crafts alkylation	59
	LmrR	SCS	Staudinger reduction to obtain *p*AmF	Organocatalytic residue in tandem Friedel-Crafts alkylation/ enantioselective-protonation	60
	LmrR	SCS	Staudinger reduction to obtain *p*AmF	Organocatalytic residue in synergistic catalysis of Michael addition reactions with supramolecular Cu^{II}Phen	61
	LmrR	SCS	Staudinger reduction to obtain *p*AmF	Organocatalytic residue in synergistic catalysis of Michael addition/enantioselective protonation with supramolecular Cu^{II}Phen	62
	tHisF	SCS	Covalent attachment of dirhodium complex via SPAAC	Bio-orthogonal handle in ArM catalyzing intermolecular cyclopropanation and Si-H insertion reactions	63
	POP	SCS	Covalent attachment of dirhodium complex via SPAAC	Bio-orthogonal handle in ArM catalyzing intermolecular cyclopropanation and carbene insertion reactions.	64,65
	POP	SCS	Covalent attachment of Acr^{+}-MES via SPAAC	Bio-orthogonal handle in artificial photoenzyme catalyzing sulfoxidation of thioanisoles	66
	POP	SCS	Covalent attachment of $Ru^{II}(Bpy)_3$ cofactor via SPAAC	Bio-orthogonal handle in artificial photoenzyme catalyzing reductive cyclization and [2 + 2] cycloaddition reactions	67
NMH	LmrR	SCS	-	Photosensitizer in an artificial photoenzyme catalyzing enantioselective in the	56

ncAA	Protein host	Method of incorporation	Post-translational modification (includes cofactor addition)	Activity/Catalysis	Ref.
				intramolecular [2+2] cycloadditions of indoles	
	Mb	SCS	-	Heme proximal ligand exchange in carbene transferase activity	68,69,70
	BH32	SCS	-	Organocatalytic residue in an artificial esterase	71
	BH32.8	SCS	-	Organocatalytic residue in a Morita-Bayliss-Hillman reaction	72
L-DOPA	Mb	SCS	-	Horseradish peroxidase His/Arg pair mimic in redox activity	73
*p*BoF	LmrR	SCS	-	Organocatalytic residue in kinetic resolution of hydroxyketones by oxime formation	74
BpyA	CAP	SCS	Cu^{II} complexation	Metal-binding residue in artificial endonuclease cleaving DNA	75
	p19	SCS	Cu^{II} complexation	Metal-binding residue in artificial endonuclease cleaving RNA	76
	LmrR	SCS	Cu^{II} complexation	Metal-binding residue in ArM catalyzing enantioselective Friedel-Crafts alkylations	77
	LmrR	SCS	Cu^{II} complexation	Metal-binding residue in ArM catalyzing enantioselective enone hydrations	78
	QaqR, RamR & CgmR	SCS	Cu^{II} complexation	Metal-binding residue in ArM catalyzing enantioselective Friedel-Crafts alkylations	79
	SCP	SCS	Cu^{II} complexation	Metal-binding residue in ArM catalyzing enantioselective Friedel-Crafts alkylations	80
	acetyltransferase from *Bacillus anthracis*	SCS	Cu^{II} complexation	Metal-binding residue in an artificial dicopper oxidase	81
	Apo-Mb	SCS	Ni^{II} complexation and covalent attachment of Iridium photosensitizer via cysteine conjugation	Metal-binding residue in artificial photoenzyme catalyzing the hydroxylation of aryl halides	82

**ncAA**	**Protein host**	**Method of incorporation**	**Post-translational modification (includes cofactor addition)**	**Activity/Catalysis**	**Ref.**
D/L-PyK	LmrR	SCS	-	Organocatalytic residue in vinylogous Henry reaction	[83]
D/L-PiK	LmrR	SCS	-	Organocatalytic residue in vinylogous Henry reaction	[83]
HQA-1	LmrR	SCS	Cu^{II} complexation	Metal-binding residue in ArM catalyzing enantioselective Friedel-Crafts alkylation and enone hydration reactions	[84]
HQA-2	HaloTag	SCS	Complexation with $[(\eta^5\text{-}C_5H_5)Ru(MeCN)_3]+$	Metal-binding residue in an artificial deallylase	[85]
imiY	Mb	SCS	-	HCOs Tyr-His cross link mimic in redox activity	[86]
(2,3,5-F_3)Y	Mb	SCS	-	HCOs Tyr-His cross link mimic in redox activity	[46]
*m*ClDY	Mb	SCS	-	HCOs Tyr-His cross link mimic in redox activity	[46]
*m*AmY	Mb	SCS	-	Horseradish peroxidase His/Arg pair mimic in redox activity	[87]
3ThA	Mb	SCS	-	Heme proximal ligand exchange in carbene and nitrene transferase activity	[49, 70]
4ThzA	Mb	SCS	-	Heme proximal ligand exchange in carbene transferase activity	[70]
5ThzA	Mb	SCS	-	Heme proximal ligand exchange in carbene transferase activity	[70]
3PyA	Mb	SCS	-	Heme proximal ligand exchange in carbene and nitrene transferase activity	[49]
FBzF	LmrR	SCS	-	Photosensitizer in an artificial photoenzyme catalyzing enantioselective in the intramolecular [2+2] cycloadditions of indoles	[56]

REFERENCES

1. Parsons, J. F.; Armstrong, R. N. Proton Configuration in the Ground State and Transition State of a Glutathione Transferase-Catalyzed Reaction Inferred from the Properties of Tetradeca(3-Fluorotyrosyl)Glutathione Transferase. *J. Am. Chem. Soc.* **1996**, *118*, 2295-2296.
2. Thorson, J. S.; Shin, I.; Chapman, E.; Stenberg, G.; Mannervik, B.; Schultz, P. G. Analysis of the Role of the Active Site Tyrosine in Human Glutathione Transferase A1-1 by Unnatural Amino Acid Mutagenesis. *J. Am. Chem. Soc.* **1998**, *120*, 451-452.
3. Lin, C.-Y.; Muñoz Hernández, A. L.; Laremore, T. N.; Silakov, A.; Krebs, C.; Boal, A. K.; Bollinger, J. M. J. Use of Noncanonical Tyrosine Analogues to Probe Control of Radical Intermediates During Endoperoxide Installation by Verruculogen Synthase (Ftmox1). *ACS Catal.* **2022**, *12*, 6968-6979.
4. Parsons, J. F.; Xiao, G.; Gilliland, G. L.; Armstrong, R. N. Enzymes Harboring Unnatural Amino Acids: Mechanistic and Structural Analysis of the Enhanced Catalytic Activity of a Glutathione Transferase Containing 5-Fluorotryptophan, *Biochemistry* **1998**, *37*, 6286-6294.
5. Mehta, K. R.; Yang, C. Y.; Montclare, J. K. Modulating Substrate Specificity of Histone Acetyltransferase with Unnatural Amino Acids. *Molecular BioSystems* **2011**, *7*, 3050-3055.
6. Dominguez, M. A.; Thornton, K. C.; Melendez, M. G.; Dupureur, C. M. Differential Effects of Isomeric Incorporation of Fluorophenylalanines into Pvuii Endonuclease. *Proteins: Struct., Funct., Bioinf.* **2001**, *45*, 55-61.
7. Chen, H.; Wu, J.; Yang, L.; Xu, G. Characterization and Structure Basis of Pseudomonas Alcaligenes Lipase's Enantiopreference Towards D,L-Menthyl Propionate. *J. Mol. Catal. B: Enzym.* **2014**, *102*, 81-87.
8. Morikubo, N.; Fukuda, Y.; Ohtake, K.; Shinya, N.; Kiga, D.; Sakamoto, K.; Asanuma, M.; Hirota, H.; Yokoyama, S.; Hoshino, T. Cation−Π Interaction in the Polyolefin Cyclization Cascade Uncovered by Incorporating Unnatural Amino Acids into the Catalytic Sites of Squalene Cyclase. *J. Am. Chem. Soc.* **2006**, *128*, 13184-13194.
9. Ma, H.; Yang, X.; Lu, Z.; Liu, N.; Chen, Y. The "Gate Keeper" Role of Trp222 Determines the Enantiopreference of Diketoreductase toward 2-Chloro-1-Phenylethanone. *PLoS One* **2014**, *9*, No. e103792.
10. Faraldos, J. A.; Antonczak, A. K.; González, V.; Fullerton, R.; Tippmann, E. M.; Allemann, R. K. Probing Eudesmane Cation−Π Interactions in Catalysis by Aristolochene Synthase with Non-Canonical Amino Acids. *J. Am. Chem. Soc.* **2011**, *133*, 13906-13909.
11. Englert, M.; Nakamura, A.; Wang, Y.-S.; Eiler, D.; Söll, D.; Guo, L.-T. Probing the Active Site Tryptophan of Staphylococcus Aureus Thioredoxin with an Analog. *Nucleic Acids Res.* **2015**, *43*, 11061-11067.
12. Chen, L.; Naowarojna, N.; Song, H.; Wang, S.; Wang, J.; Deng, Z.; Zhao, C.; Liu, P. Use of a Tyrosine Analogue to Modulate the Two Activities of a Nonheme Iron Enzyme Ovoa in Ovothiol Biosynthesis, Cysteine Oxidation Versus Oxidative C–S Bond Formation. *J. Am. Chem. Soc.* **2018**, *140*, 4604-4612.
13. Chen, L.; Naowarojna, N.; Chen, B.; Xu, M.; Quill, M.; Wang, J.; Deng, Z.; Zhao, C.; Liu, P. Mechanistic Studies of a Nonheme Iron Enzyme Ovoa in Ovothiol Biosynthesis Using a Tyrosine Analogue, 2-Amino-3-(4-Hydroxy-3-(Methoxyl) Phenyl) Propanoic Acid (Meotyr). *ACS Catal.* **2019**, *9*, 253-258.
14. Wu, Y.; Boxer, S. G. A Critical Test of the Electrostatic Contribution to Catalysis with Noncanonical Amino Acids in Ketosteroid Isomerase. *J. Am. Chem. Soc.* **2016**, *138*, 11890-11895.
15. Yu, Z.; Yu, H.; Tang, H.; Wang, Z.; Wu, J.; Yang, L.; Xu, G. Site-Specifically Incorporated Non-Canonical Amino Acids into *Pseudomonas Alcaligenes* Lipase to Hydrolyze L-Menthol Propionate among the Eight Isomers. *ChemCatChem* **2021**, *13*, 2691-2701.
16. Voller, J.; Biava, H.; Koksch, B.; Hildebrandt, P.; Budisa, N. Orthogonal Translation Meets Electron Transfer: In Vivo Labeling of Cytochrome C for Probing Local Electric Fields. *ChemBioChem* **2015**, *16*, 742-745.

17. An, X.; Chen, C.; Wang, T.; Huang, A.; Zhang, D.; Han, M.-J.; Wang, J. Genetic Incorporation of Selenotyrosine Significantly Improves Enzymatic Activity of Agrobacterium Radiobacter Phosphotriesterase. *ChemBioChem* **2021**, *22*, 2535-2539.
18. Sharma, V.; Wang, Y.-S.; Liu, W. R. Probing the Catalytic Charge-Relay System in Alanine Racemase with Genetically Encoded Histidine Mimetics. *ACS Chem. Biol.* **2016**, *11*, 3305-3309.
19. Wilkinson, H. C.; Dalby, P. A. Fine-Tuning the Activity and Stability of an Evolved Enzyme Active-Site through Noncanonical Amino-Acids. *FEBS J.* **2021**, *288*, 1935-1955.
20. Pagar, A. D.; Jeon, H.; Khobragade, T. P.; Sarak, S.; Giri, P.; Lim, S.; Yoo, T. H.; Ko, B. J.; Yun, H. Non-Canonical Amino Acid-Based Engineering of (*R*)-Amine Transaminase. *Front. Chem.* **2022**, *10*, 839636.
21. Lars, M.; Melina, S.; Garabed, A.; Nediljko, B. Parallel Incorporation of Different Fluorinated Amino Acids: On the Way to "Teflon" Proteins. *ChemBioChem* **2010**. *11*, 1505-1507.
22. Hoesl, M. G.; Acevedo-Rocha, C. G.; Nehring, S.; Royter, M.; Wolschner, C.; Wiltschi, B.; Budisa, N.; Antranikian, G. Lipase Congeners Designed by Genetic Code Engineering. *ChemCatChem* **2011**, *3*, 213-221.
23. Haernvall, K.; Fladischer, P.; Schoeffmann, H.; Zitzenbacher, S.; Pavkov-Keller, T.; Gruber, K.; Schick, M.; Yamamoto, M.; Kuenkel, A.; Ribitsch, D.; Guebitz, G. M.; Wiltschi, B. Residue-Specific Incorporation of the Non-Canonical Amino Acid Norleucine Improves Lipase Activity on Synthetic Polyesters. *Front. Bioeng. Biotechnol.* **2022**, *10*, 769830.
24. Hoesl, M. G.; Budisa, N. Expanding and Engineering the Genetic Code in a Single Expression Experiment. *ChemBioChem* **2011**, *12*, 552-555.
25. Ugwumba, I. N.; Ozawa, K.; Xu, Z.-Q.; Ely, F.; Foo, J.-L.; Herlt, A. J.; Coppin, C.; Brown, S.; Taylor, M. C.; Ollis, D. L.; Mander, L. N.; Schenk, G.; Dixon, N. E.; Otting, G.; Oakeshott, J. G.; Jackson, C. J. Improving a Natural Enzyme Activity through Incorporation of Unnatural Amino Acids. *J. Am. Chem. Soc.* **2011**, *133*, 326-333.
26. Xiao, H.; Nasertorabi, F.; Choi, S.-H.; Han, G. W.; Reed, S. A.; Stevens, R. C.; Schultz, P. G. Exploring the Potential Impact of an Expanded Genetic Code on Protein Function. *Proc. Natl. Acad. Sci.* **2015**, *112*, 6961-6966.
27. Drienovská, I.; Gajdoš, M.; Kindler, A.; Takhtehchian, M.; Darnhofer, B.; Birner-Gruenberger, R.; Dörr, M.; Bornscheuer, U. T.; Kourist, R. Folding Assessment of Incorporation of Noncanonical Amino Acids Facilitates Expansion of Functional-Group Diversity for Enzyme Engineering. *Chem. Eur. J.* **2020**, *26*, 12338-12342.
28. Cirino, P. C.; Tang, Y.; Takahashi, K.; Tirrell, D. A.; Arnold, F. H. Global Incorporation of Norleucine in Place of Methionine in Cytochrome P450 Bm-3 Heme Domain Increases Peroxygenase Activity. *Biotechnol. Bioeng.* **2003**, *83*, 729-734.
29. Kolev, J. N.; Zaengle, J. M.; Ravikumar, R.; Fasan, R. Enhancing the Efficiency and Regioselectivity of P450 Oxidation Catalysts by Unnatural Amino Acid Mutagenesis. *ChemBioChem* **2014**, *15*, 1001-1010.
30. Jackson, J. C.; Duffy, S. P.; Hess, K. R.; Mehl, R. A. Improving Nature's Enzyme Active Site with Genetically Encoded Unnatural Amino Acids. *J. Am. Chem. Soc.* **2006**, *128*, 11124-11127.
31. Zheng, S.; Lim, S. I.; Kwon, I. Manipulating the Substrate Specificity of Murine Dihydrofolate Reductase Enzyme Using an Expanded Set of Amino Acids. *Biochem. Eng. J.* **2015**, *99*, 85-92.
32. Zheng, S.; Kwon, I. Controlling Enzyme Inhibition Using an Expanded Set of Genetically Encoded Amino Acids. *Biotechnology and Bioengineering* **2013**, *110*, 2361-2370.
33. Green, A. P.; Hayashi, T.; Mittl, P. R. E.; Hilvert, D. A Chemically Programmed Proximal Ligand Enhances the Catalytic Properties of a Heme Enzyme. *J. Am. Chem. Soc.* **2016**, *138*, 11344-11352.
34. Bhagat, A. K.; Buium, H.; Shmul, G.; Alfonta, L. Genetically Expanded Reactive-Oxygen-Tolerant Alcohol Dehydrogenase Ii. *ACS Catal.* **2020**, *10*, 3094-3102.
35. Kofoed, C.; Riesenberg, S.; Šmolíková, J.; Meldal, M.; Schoffelen, S. Semisynthesis of an Active Enzyme by Quantitative Click Ligation. *Bioconjugate Chem.* **2019**, *30*, 1169-1174.
36. Zubi, Y. S.; Seki, K.; Li, Y.; Hunt, A. C.; Liu, B.; Roux, B.; Jewett, M. C.; Lewis, J. C. Metal-Responsive Regulation of Enzyme Catalysis Using Genetically Encoded Chemical Switches. *Nat. Commun.* **2022**, *13*, 1864.

37. Li, H.; Wang, R.; Wang, A.; Zhang, J.; Yin, Y.; Pei, X.; Zhang, P. Rapidly and Precisely Cross-Linked Enzymes Using Bio-Orthogonal Chemistry from Cell Lysate for the Synthesis of (*S*)-1-(2,6-Dichloro-3-Fluorophenyl) Ethanol. *ACS Sustainable Chem. Eng.* **2020**, *8*, 6466-6478.
38. Yin, Y.; Wang, R.; Zhang, J.; Luo, Z.; Xiao, Q.; Xie, T.; Pei, X.; Gao, P.; Wang, A. Efficiently Enantioselective Hydrogenation Photosynthesis of (*R*)-1-[3,5-Bis(Trifluoromethyl)Phenyl] Ethanol over a Cles-Tio2 Bioinorganic Hybrid Materials. *ACS Appl. Mater. Interfaces* **2021**, *13*, 41454-41463.
39. Bundy, B. C.; Swartz, J. R. Site-Specific Incorporation of *P*-Propargyloxyphenylalanine in a Cell-Free Environment for Direct Protein−Protein Click Conjugation. *Bioconjugate Chem.* **2010**, *21*, 255-263.
40. Kim, S.; Ko, W.; Sung, B. H.; Kim, S. C.; Lee, H. S. Direct Protein–Protein Conjugation by Genetically Introducing Bioorthogonal Functional Groups into Proteins. *Bioorg. Med. Chem.* **2016**, *24*, 5816-5822.
41. Lim, S. I.; Cho, J.; Kwon, I. Double Clicking for Site-Specific Coupling of Multiple Enzymes. *Chem. Commun.* **2015**, *51*, 13607-13610.
42. Lim, S. I.; Yang, B.; Jung, Y.; Cha, J.; Cho, J.; Choi, E.-S.; Kim, Y. H.; Kwon, I. Controlled Orientation of Active Sites in a Nanostructured Multienzyme Complex. *Sci. Rep.* **2016**, *6*, 39587.
43. Wang, R.; Zhang, J.; Luo, Z.; Xie, T.; Xiao, Q.; Pei, X.; Wang, A. Controllably Crosslinked Dual Enzymes Enabled by Genetic-Encoded Non-Standard Amino Acid for Efficiently Enantioselective Hydrogenation. *Int. J. Biol. Macromol.* **2022**, *205*, 682-691.
44. Qiao, L.; Luo, Z.; Wang, R.; Pei, X.; Wu, S.; Chen, H.; Xie, T.; Sheldon, R. A.; Wang, A. Designing an Enzyme Assembly Line for Green Cascade Processes Using Bio-Orthogonal Chemistry. *Green Chem.* **2023**, *25*, 7547-7555.
45. Schoffelen, S.; Beekwilder, J.; Debets, M. F.; Bosch, D.; Hest, J. C. M. v. Construction of a Multifunctional Enzyme Complex Via the Strain-Promoted Azide–Alkyne Cycloaddition. *Bioconjugate Chem.* **2013**, *24*, 987-996.
46. Yu, Y.; Lv, X.; Li, J.; Zhou, Q.; Cui, C.; Hosseinzadeh, P.; Mukherjee, A.; Nilges, M. J.; Wang, J.; Lu, Y. Defining the Role of Tyrosine and Rational Tuning of Oxidase Activity by Genetic Incorporation of Unnatural Tyrosine Analogs. *J. Am. Chem. Soc.* **2015**, *137*, 4594-4597.
47. Zhou, Q.; Hu, M.; Zhang, W.; Jiang, L.; Perrett, S.; Zhou, J.; Wang, J. Probing the Function of the Tyr-Cys Cross-Link in Metalloenzymes by the Genetic Incorporation of 3-Methylthiotyrosine. *Angew. Chem., Int. Ed.* **2013**, *52*, 1203-1207.
48. Yu, Y.; Zhou, Q.; Wang, L.; Liu, X.; Zhang, W.; Hu, M.; Dong, J.; Li, J.; Lv, X.; Ouyang, H.; Li, H.; Gao, F.; Gong, W.; Lu, Y.; Wang, J. Significant Improvement of Oxidase Activity through the Genetic Incorporation of a Redox-Active Unnatural Amino Acid. *Chem. Sci.* **2015**, *6*, 3881-3885.
49. Moore, E. J.; Fasan, R. Effect of Proximal Ligand Substitutions on the Carbene and Nitrene Transferase Activity of Myoglobin. *Tetrahedron* **2019**, *75*, 2357-2363.
50. Ofori Atta, L.; Zhou, Z.; Roelfes, G. In Vivo Biocatalytic Cascades Featuring an Artificial-Enzyme-Catalysed New-to-Nature Reaction. *Angew. Chem., Int. Ed.* **2023**, *62*, No. e202214191.
51. Liu, X.; Kang, F.; Hu, C.; Wang, L.; Xu, Z.; Zheng, D.; Gong, W.; Lu, Y.; Ma, Y.; Wang, J. A Genetically Encoded Photosensitizer Protein Facilitates the Rational Design of a Miniature Photocatalytic Co_2-Reducing Enzyme. *Nat. Chem.* **2018**, *10*, 1201-1206.
52. Kang, F.; Yu, L.; Xia, Y.; Yu, M.; Xia, L.; Wang, Y.; Yang, L.; Wang, T.; Gong, W.; Tian, C.; Liu, X.; Wang, J. Rational Design of a Miniature Photocatalytic Co_2-Reducing Enzyme. *ACS Catal.* **2021**, *11*, 5628-5635.
53. Fu, Y.; Huang, J.; Wu, Y.; Liu, X.; Zhong, F.; Wang, J. Biocatalytic Cross-Coupling of Aryl Halides with a Genetically Engineered Photosensitizer Artificial Dehalogenase. *J. Am. Chem. Soc.* **2021**, *143*, 617-622.
54. Fu, Y.; Liu, X.; Xia, Y.; Guo, X.; Guo, J.; Zhang, J.; Zhao, W.; Wu, Y.; Wang, J.; Zhong, F. Whole-Cell-Catalyzed Hydrogenation/Deuteration of Aryl Halides with a Genetically Repurposed Photodehalogenase. *Chem.* **2023**, *9*, 1897-1909.
55. Trimble, J. S.; Crawshaw, R.; Hardy, F. J.; Levy, C. W.; Brown, M. J. B.; Fuerst, D. E.; Heyes, D. J.; Obexer, R.; Green, A. P. A Designed Photoenzyme for Enantioselective [2+2] Cycloadditions. *Nature* **2022**, *611*, 709-714.

56. Sun, N.; Huang, J.; Qian, J.; Zhou, T. P.; Guo, J.; Tang, L.; Zhang, W.; Deng, Y.; Zhao, W.; Wu, G.; Liao, R. Z.; Chen, X.; Zhong, F.; Wu, Y. Enantioselective [2+2]-Cycloadditions with Triplet Photoenzymes. *Nature* **2022**, *611*, 715-720.
57. Drienovska, I.; Mayer, C.; Dulson, C.; Roelfes, G. A Designer Enzyme for Hydrazone and Oxime Formation Featuring an Unnatural Catalytic Aniline Residue. *Nat. Chem.* **2018**, *10*, 946-952.
58. Mayer, C.; Dulson, C.; Reddem, E.; Thunnissen, A. W. H.; Roelfes, G. Directed Evolution of a Designer Enzyme Featuring an Unnatural Catalytic Amino Acid. *Angew. Chem., Int. Ed.* **2019**, *58*, 2083-2087.
59. Leveson-Gower, R. B.; Zhou, Z.; Drienovska, I.; Roelfes, G. Unlocking Iminium Catalysis in Artificial Enzymes to Create a Friedel-Crafts Alkylase. *ACS Catal.* **2021**, *11*, 6763-6770.
60. Leveson-Gower, R. B.; de Boer, R. M.; Roelfes, G. Tandem Friedel-Crafts-Alkylation-Enantioselective-Protonation by Artificial Enzyme Iminium Catalysis. *ChemCatChem* **2022**, *14*, No. e202101875.
61. Zhou, Z.; Roelfes, G. Synergistic Catalysis in an Artificial Enzyme by Simultaneous Action of Two Abiological Catalytic Sites. *Nat. Catal.* **2020**, *3*, 289-294.
62. Zhou, Z.; Roelfes, G. Synergistic Catalysis of Tandem Michael Addition/Enantioselective Protonation Reactions by an Artificial Enzyme. *ACS Catal.* **2021**, *11*, 9366-9369.
63. Yang, H.; Srivastava, P.; Zhang, C.; Lewis, J. C. A General Method for Artificial Metalloenzyme Formation through Strain-Promoted Azide-Alkyne Cycloaddition. *ChemBioChem* **2014**, *15*, 223-227.
64. Srivastava, P.; Yang, H.; Ellis-Guardiola, K.; Lewis, J. C. Engineering a Dirhodium Artificial Metalloenzyme for Selective Olefin Cyclopropanation. *Nat. Commun.* **2015**, *6*, 7789.
65. Yang, H.; Swartz, A. M.; Park, H. J.; Srivastava, P.; Ellis-Guardiola, K.; Upp, D. M.; Lee, G.; Belsare, K.; Gu, Y.; Zhang, C.; Moellering, R. E.; Lewis, J. C. Evolving Artificial Metalloenzymes Via Random Mutagenesis. *Nat. Chem.* **2018**, *10*, 318-324.
66. Gu, Y.; Ellis-Guardiola, K.; Srivastava, P.; Lewis, J. C. Preparation, Characterization, and Oxygenase Activity of a Photocatalytic Artificial Enzyme. *ChemBioChem* **2015**, *16*, 1880-1883.
67. Zubi, Y. S.; Liu, B.; Gu, Y.; Sahoo, D.; Lewis, J. C. Controlling the Optical and Catalytic Properties of Artificial Metalloenzyme Photocatalysts Using Chemogenetic Engineering. *Chem. Sci.* **2022**, *13*, 1459-1468.
68. Hayashi, T.; Tinzl, M.; Mori, T.; Krengel, U.; Proppe, J.; Soetbeer, J.; Klose, D.; Jeschke, G.; Reiher, M.; Hilvert, D. Capture and Characterization of a Reactive Haem–Carbenoid Complex in an Artificial Metalloenzyme. *Nat. Catal.* **2018**, *1*, 578-584.
69. Carminati, D. M.; Fasan, R. Stereoselective Cyclopropanation of Electron-Deficient Olefins with a Cofactor Redesigned Carbene Transferase Featuring Radical Reactivity. *ACS Catal.* **2019**, *9*, 9683-9697.
70. Pott, M.; Tinzl, M.; Hayashi, T.; Ota, Y.; Dunkelmann, D.; Mittl, P. R. E.; Hilvert, D. Noncanonical Heme Ligands Steer Carbene Transfer Reactivity in an Artificial Metalloenzyme. *Angew. Chem., Int. Ed.* **2021**, *60*, 15063-15068.
71. Burke, A. J.; Lovelock, S. L.; Frese, A.; Crawshaw, R.; Ortmayer, M.; Dunstan, M.; Levy, C.; Green, A. P. Design and Evolution of an Enzyme with a Non-Canonical Organocatalytic Mechanism. *Nature* **2019**, *570*, 219-223.
72. Hutton, A. E.; Foster, J.; Crawshaw, R.; Hardy, F. J.; Johannissen, L. O.; Lister, T. M.; Gerard, E. F.; Birch-Price, Z.; Obexer, R.; Hay, S.; Green, A. P. A Non-Canonical Nucleophile Unlocks a New Mechanistic Pathway in a Designed Enzyme. *Nat. Commun*. 2024, 15, 1956.
73. Chand, S.; Ray, S.; Yadav, P.; Samanta, S.; Pierce, B. S.; Perera, R. Abiological Catalysis by Myoglobin Mutant with a Genetically Incorporated Unnatural Amino Acid. *Biochem. J.* **2021**, *478*, 1795-1808.
74. Longwitz, L.; Leveson-Gower, R. B.; Rozeboom, H. J.; Thunnissen, A. W. H.; Roelfes, G. Boron Catalysis in a Designer Enzyme. *Nature* **2024**. *629*, 824–829.
75. Lee, H. S.; Schultz, P. G. Biosynthesis of a Site-Specific DNA Cleaving Protein. *J. Am. Chem. Soc.* **2008**, *130*, 13194-13195.
76. Ahmed, N.; Ahmed, N.; Bilodeau, D. A.; Pezacki, J. P. An Unnatural Enzyme with Endonuclease Activity Towards Small Non-Coding Rnas. *Nat. Commun.* **2023**, *14*, 3777.

77. Drienovska, I.; Rioz-Martinez, A.; Draksharapu, A.; Roelfes, G. Novel Artificial Metalloenzymes by in Vivo Incorporation of Metal-Binding Unnatural Amino Acids. *Chem. Sci.* **2015**, *6*, 770-776.
78. Drienovska, I.; Alonso-Cotchico, L.; Vidossich, P.; Lledos, A.; Marechal, J. D.; Roelfes, G. Design of an Enantioselective Artificial Metallo-Hydratase Enzyme Containing an Unnatural Metal-Binding Amino Acid. *Chem. Sci.* **2017**, *8*, 7228-7235.
79. Bersellini, M.; Roelfes, G. Multidrug Resistance Regulators (Mdrs) as Scaffolds for the Design of Artificial Metalloenzymes. *Org. Biomol. Chem.* **2017**, *15*, 3069-3073.
80. Klemencic, E.; Brewster, R. C.; Ali, H. S.; Richardson, J. M.; Jarvis, A. G. Using Bpyala to Generate Copper Artificial Metalloenzymes: A Catalytic and Structural Study. *Catal. Sci. Technol.* **2024**, *14*, 1622-1632.
81. Jung, S. M.; Yang, M.; Song, W. J. Symmetry-Adapted Synthesis of Dicopper Oxidases with Divergent Dioxygen Reactivity. *Inorg. Chem.* **2022**, *61*, 12433-12441.
82. Lee, J.; Song, W. J. Photocatalytic C-O Coupling Enzymes That Operate Via Intramolecular Electron Transfer. *J. Am. Chem. Soc.* **2023**, *145*, 5211-5221.
83. Gran-Scheuch, A.; Bonandi, E.; Drienovská, I. Expanding the Genetic Code: Incorporation of Functional Secondary Amines Via Stop Codon Suppression. *ChemCatChem* **2023**, *16*, No. e202301004.
84. Drienovská, I.; Scheele, R. A.; de Souza, C. G.; Roelfes, G. A Hydroxyquinoline-Based Unnatural Amino Acid for the Design of Novel Artificial Metalloenzymes. *ChemBioChem* **2020**, *21*, 3077-3081.
85. Stein, A.; Liang, A. D.; Sahin, R.; Ward, T. R. Incorporation of Metal-Chelating Unnatural Amino Acids into Halotag for Allylic Deamination. *J. Organomet. Chem.* **2022**, *962*, 122272.
86. Liu, X.; Yu, Y.; Hu, C.; Zhang, W.; Lu, Y.; Wang, J. Significant Increase of Oxidase Activity through the Genetic Incorporation of a Tyrosine–Histidine Cross-Link in a Myoglobin Model of Heme–Copper Oxidase. *Angew. Chem., Int. Ed.* **2012**, *51*, 4312-4316.
87. Chand, S.; Ray, S.; Wanigasekara, E.; Yadav, P.; Crawford, J. A.; Armstrong, D. W.; Rajeshwar, K.; Pierce, B. S. Improved Rate of Substrate Oxidation Catalyzed by Genetically-Engineered Myoglobin. *Arch. Biochem. Biophys.* **2018**, *639*, 44-51.
